# Supplementary material for: Supporting antidepressant discontinuation: the development and optimisation of a digital intervention for patients in UK primary care using a theory, evidence and person-based approach
Source: BMJ Open. 2020 Mar 8;10(3):e032312. doi: 10.1136/bmjopen-2019-032312 (PMC7064123; doi:10.1136/bmjopen-2019-032312)
Supplement: Supplementary data [file bmjopen-2019-032312supp001.pdf]

Appendix A – Interview Schedule

REDUCE Study Workstream (WS) 3: REviewing long-term anti-Depressant treatment Use by Careful monitoring in Everyday practice

THINK-ALOUD INTERVIEW SCHEDULE WITH PATIENTS

Below is a list of topics/questions to be discussed in this study. The qualitative work will remain flexible with respect to participants' agendas but we will cover the broad topics/questions noted. It is common in qualitative work to iteratively develop topics and questions as new ideas emerge from early data collection. Therefore, we may add new topics as the interviews progress and data collection continues. However, the key topics of exploring participants' views of the prototype intervention will remain the same.

Introduction

1. Re-introduce self and purpose of interview

2. Check with participant:

- That they are still willing to be interviewed, and to be audio recorded
- Remind them it will take approximately 60 to 90 minutes
- That they are comfortable in a quiet place where they will not be disturbed

3. Remind participant that:

- Their responses will be kept confidential, and quotes used in the results will not identify them as an individual;
- They can change their mind about taking part in the study and stop the interview at any point.

4. Remind the participant that you will start by asking them some questions about their experiences with antidepressants. Remind the participant that you want them to look at the website and use it as they normally would, but say everything that they are thinking out loud. Tell them that you will remind them to do this so that they don't forget as it is very easy to forget and that there are no right or wrong answers as it is their views that are important to us.

5. Ask if the participant has any questions.

6. Start recording.

Bowers HM, *et al. BMJ Open* 2020; 10:e032312. doi: 10.1136/bmjopen-2019-032312

Section 1: Demographic Data

We would like to collect some personal information to help us describe the range of people / experiences we have collected, so could you please let me know your

Age	
Gender	M / F
Do you live alone or with someone (friends / partner / family)?	
Single / in a relationship / married?	
Employed / retired / full time carer / stay at home parent?	
Job title	
Currently on ADs?	Y / N
Successfully stopped ADs before?<br>NB. 'Success' = been off ADs & experienced symptom free episode(s).	Y / N
Same GP for review or different GPs within practice?	
Current Medical Diagnosis for ADs (if known)	
Do you pay for your prescriptions?	
Have you ever taken any sick leave from work due to depression / anxiety / stress? If yes, how much?	
Have you ever needed a carer/ or to be cared for due to depression? If yes, by whom?	
Any other medical conditions?	
Have you ever taken St John's Wort?	
Any other relevant information?	
Participant ID	
Date screened by researcher / confirm eligible	
Urban or rural location? (*researcher observation*)	
Deprivation level of area? (*researcher observation*)	

Section 2: Background history of use of antidepressants.

1. <u>Can you tell me a little bit about when you were first prescribed antidepressants?</u>

Prompt: Feelings about how decision to go on antidepressants was made/managed. Experience of taking ADs.

2. <u>Could you describe your experience of taking antidepressants for me now?</u>

Prompt: Any intent to stop? Have you found antidepressants have helped to improve your condition? Side effects/benefits? Expectations of ADs vs. lived experience.

3. <u>Can you tell me about your current depression treatment?</u>

Prompt:
- Regular repeat prescriptions?
- Any self-help or counselling / therapy?
- How often are you reviewed by a GP, nurse or counsellor/therapist? Feelings around frequency?
- Continuity of care?

Bowers HM, *et al. BMJ Open* 2020; 10:e032312. doi: 10.1136/bmjopen-2019-032312

- What treatment would you say has helped you most / least?

Section 3: Previous attempts to discontinue / successful withdrawal. Barriers and enablers to discontinuation (including individual / social factors).

1. Can you tell me about a time when you stopped or thought about stopping your antidepressants?

Prompt: What were your reasons for wanting to stop? How long did you stop for? What was it that made you stay on your antidepressants? Withdrawal experiences / effects. How would you feel if you had to restart your antidepressants or increase the dose (if stopped/stopping)? Explore expectations around withdrawal.

Section 4: Think-aloud and researcher prompts

Explain to them that you want them to look at the website and use it as they normally would, but say everything that they are thinking out loud. Tell them that you will remind them to do this so that they don't forget as it is very easy to forget. If you think it would help then get them to try counting the windows in their house whilst saying everything that they are thinking out loud.

- [only on first page] What are your first impressions of this page?
- What are you thinking now?
- What made you choose that option?
- What do you think about [this activity, this information]?
- Can you tell me a bit more about that?
- What is it you like about that?
- That's really interesting……

Section 5: Post-think-aloud questions

- Overall, what do you think about this website?
- Can you tell me about anything that you liked about the website?
- Was there anything that you found surprising in the website?
- Can you tell me anything about the website that you were less keen on?
- Can you tell me about anything that you think should be changed?
- What would you think if your GP or practice nurse asked you to use the website?
- If you were withdrawing from your antidepressants, which parts of AD-visor do you think you would like to look at and why? (E.g. dealing with withdrawal symptoms, information about how antidepressants work, relapse prevention, mindfulness etc.).
- When people use this website for real, they will be offered some support over the telephone. If you were using the programme for real, what would you think of this option to get support over the phone?
- What are your thoughts about telephone support throughout the trial in general? [Researcher to explain trial design].
- If you did have opportunity to have support over the telephone, which of the topics in AD-visor do you think would be most useful to discuss over the phone?

ANY OTHER TOPICS YOU WOULD LIKE TO DISCUSS?

ANY QUESTIONS?

Interview schedule v2.1 06.10.2017 REDUCE WS3 PATIENTS IRAS REF: 231064

Debrief

- Tell participant that the digital recorder is now being switched off.
- Thank participant for taking part in the interview.
- Revisit consent
- Ask if the participant has any questions about the study.
- Let them know that you will be sending all participants a summary of study findings.
- Check happy for data to be used for teaching / secondary analysis.
- Thank participant again for taking part in the interview.

Bowers HM, *et al. BMJ Open* 2020; 10:e032312. doi: 10.1136/bmjopen-2019-032312
