# Supplementary material for: Supporting antidepressant discontinuation: the development and optimisation of a digital intervention for patients in UK primary care using a theory, evidence and person-based approach
Source: BMJ Open. 2020 Mar 8;10(3):e032312. doi: 10.1136/bmjopen-2019-032312 (PMC7064123; doi:10.1136/bmjopen-2019-032312)
Supplement: Supplementary data [file bmjopen-2019-032312supp002.pdf]

Appendix B – Behavioural Diagnosis

Target behaviour: Reducing and stopping antidepressant medication		
**BCW/COM-B Components**	**What needs to happen for the target behaviour to occur?**	**Proposed intervention element**
**Physical capability**<br>*Physical skill, strength or stamina*	• Understanding how to reduce doses physically: e.g. how to take tapered medication appropriately, in order to reduce the occurrence of side effects.	• **GP**<br>• **Internet intervention modules**<br>• **Telephone support**
**Psychological capability**<br>*Knowledge or psychological skills, strength or stamina to engage in necessary mental processes*	• Detailed, accessible guidance on the withdrawal process in general (setting up appropriate expectations)<br>• Improving knowledge on how to withdraw (practicalities)<br>• Developing psychological skills to manage the process:<br>o Managing psychological side effects of withdrawal<br>o Understanding helpful appraisals of symptoms<br>o Learning about the prevention of relapse, managing fear of recurrence<br>o Developing skills to manage life-stressors cognitively and behaviourally<br>*Social Cognitive Theory (SCT) and research will be broadly drawn on to ensure information/techniques are described and applied to align with evidence-based principles for increasing self-efficacy*	• **Internet intervention modules**<br>• (Telephone support)

**Physical opportunity**<br>*Opportunity afforded by the environment involving time recourses, locations, cues, physical affordance*	• Ability to access and get to GP appointments/pharmacy to collect reduced dose antidepressants	• **General practitioner (as a function of usual care)**<br>• **Telephone support/advice**
**Social opportunity**<br>*Opportunity afforded by interpersonal influences, social cues and cultural norms that influence the way we think about things*	• Close social network (family/friends) of patient may need to be supportive of the withdrawal process/attempt	• **Brief overview material developed for family members/friends**
**Reflective motivation**<br>*Reflective processes involving evaluations/beliefs about what is good and bad, and plans (self-conscious intentions)*	• Modification of beliefs about depression:<br>o Exploring the nature of depression in a way that aligns with behavioural/cognitive management<br>o Discussing impact of beliefs and expectations about chronicity<br>o Exploring effect of analogies with physical conditions (diabetes/asthma)<br>o Acknowledging complexity re our understanding of depression in an accessible manner<br>• Modification of beliefs about antidepressant medication:<br>o Addressing beliefs about addiction/dependency<br>o Exploring the serotonin hypothesis; evidence, balanced implications, rationale for behaviour/cognition to substitute medication	• **Internet intervention modules**<br>• **Internet intervention modules**

	• Foster motivation to withdraw through discussion of benefits, reduction of side effects, potential for increase in agency, potential for effective use of alternatives to pharmacological management<br><br>• Facilitate clear planning for the withdrawal process e.g. human contacts, management strategies, access to rapid/emergency support<br><br>*Inductive qualitative work (meta-synthesis and primary qualitative research) and theory will be used to inform this material*	• **General practitioner**<br><br>• **Telephone support/advice**
**Automatic motivation**<br>*Automatic processes involving emotional reactions, desires (wants and needs) impulses, inhibitions, drive states and reflex responses*	• Encourage awareness of automatic disruptive modes/thought process that may trigger or be triggered by symptoms<br><br>• Work on developing habitual healthier responses to symptom occurrences	• **Internet intervention modules**
**Behavourial diagnosis of the relevant COM-B components**	Although all areas of the COM-B model will need to be addressed to some extent, **psychological capability** and **reflective motivation** are likely to be the key targets for a supported digital intervention to help patients withdraw from antidepressant medication	

References:

1. Michie S, van Stralen MM, West R. The behaviour change wheel: a new method for characterising and designing behaviour change interventions. Implement Sci. 2011;6:42.
2. Michie SF, Atkins L, West R. The behaviour change wheel: a guide to designing interventions. London: Silverback Publishing; 2015
