# Supplementary material for: Supporting antidepressant discontinuation: the development and optimisation of a digital intervention for patients in UK primary care using a theory, evidence and person-based approach
Source: BMJ Open. 2020 Mar 8;10(3):e032312. doi: 10.1136/bmjopen-2019-032312 (PMC7064123; doi:10.1136/bmjopen-2019-032312)
Supplement: Supplementary data [file bmjopen-2019-032312supp003.pdf]

Appendix C – Theoretical Modelling

**Intervention module**	**Page**	**Content**	**Evidence:** Importance of barrier/facilitator content targets OR evidence for effectiveness of content	**BCW construct**	**BCW function**	**BCTs (Taxonomy V1)** Techniques broadly applied across content sections	**SCT construct** Constructs applied across content sections	**NPT construct** Constructs applied across content sections
**Reducing and stopping antidepressants**	Welcome							
	Why should I reduce and stop?	Foster a motivation to withdraw through discussion of benefits, reduction of side effects, potential for increase in agency, potential for effective use of alternatives to medication	*Bosman et al. (2016); Dickinson et al. (2010); Verbeek-Heida and Mathot (2006); Iden et al. (2011); Karp (1993); Knudsen et al. (2002); Eveleigh (2015); Gibson (2016); Schofield (2011).*	Reflexive motivation	Enablement; training; education	9.1 Credible source<br><br>9.2 Pros and cons<br><br>15.2. Persuasion about capability<br><br>13.2 Framing-reframing	Knowledge; social outcome expectations; physical outcome expectations; Self-efficacy (Somatic and emotional states)	Coherence: Individual specification<br><br>Cognitive participation: Initiation
	The downsides	Reflection on the side effects of antidepressants as a means to foster motivation to withdraw		Reflexive motivation	Enablement; training; education			

	When should I reduce and stop?	Highlighting that it is best to start withdrawal at a stable time in life		Psychological capability	Enablement; training; education			
	What to expect	Outline the discontinuation process: that the GP will provide a schedule, that this is flexible and that there may be side effects but there are ways to manage these and they are often short-lived.		Psychological capability	Enablement; training; education			
	Addressing concerns	Briefly acknowledges that many people have concerns about withdrawal but that there are techniques for dealing with this in AD-visor		Psychological capability	Enablement; training; education			
	How can my GP help?	Outline the role of the GP in discontinuation,	*Bosman et al. (2016); Dickenson et al.*	Physical capability	Enablement; training; education			

		when to go to the GP for support.	*2010; Grime & Pollock (2003); Verbeek-Heida and Mathot (2006); Eveleigh (2015); Gibson (2016); Leydon et al. (2007) ; Cartwright (2016)*					
	Planning ahead	Overview of the process: GP will give schedule and as one tapers, there is support in AD-visor that can be used		Reflexive motivation	Enablement; training; education			
	Support from family and friends	Highlight how friends and family members can play and important role	*Bosman et al. (2016); Cromartry (2011); Verbeek-Heida and Mathot (2006); Eveleigh (2015*)	Social opportunity	Enablement; training; education	3.1 Social support 3.3 Social support (emotional)		
**How to reduce antidepressants**	How to reduce	Practical information about tapering schedules		Physical capability	Enablement; training; education	4.1 Instructions on how to perform behaviour	Self-efficacy (Mastery experiences/vic	Coherence: Individual specification

	How to reduce (2)	Highlight that there is unlikely to be a need for liquid formulations or pill cutters but if needed, the GP can offer some guidance (perhaps via community pharmacist)		Physical capability	Environmental restructuring; Enablement; training; education	6.1 Demonstration of behaviour (modelling)	arious experiences).	
	When to reduce	Reiterate that there are ideal times to begin tapering, such as when no major life events are expected		Psychological capability	Enablement; training; education			
**Thinking about antidepressants**	What are antidepress ants?	Briefly explains what antidepressants are used for. Highlights that while it was believed they work through increasing serotonin, we now know it is more complex than that.	*Bosman et al. (2016); Dickenson et al. 2010; Grime & Pollock (2003); Verbeek-Heida and Mathot (2006); Karp (1993); Knudsen et al. (2002); Eveleigh (2015); Gibson (2016); Cartwright*	Reflexive motivation	Enablement; training; education	13.2 Framing/reframin g<br><br>15.2. Persuasion about capability	Social outcome expectations; Knowledge; physical outcome expectations	Coherence: Internalisation

	Can I stop taking them?	Key point: even though we don't know exactly *how* they work, we do know that many people can successfully discontinue	*(2016); Leydon et al. (2007).*	Reflexive motivation	Enablement; training; education			
	Other forms of 'antidepres sant'	There are things other than medication which can improve mood. The relationship between brain and behaviour is highlighted through a study which shows that CBT can result in changes in the brain		Reflexive motivation	Enablement; training; education			
	How to antidepress ants work	Highlights again that we don't know exactly how they work but we do know: ADs help some people and not others and many		Reflexive motivation	Enablement; training; education			

		people can successfully stop.						
**I'm worried about stopping**	I'm worried about stopping	Highlight that many people have concerns about stopping and this is understandable and does not mean you won't be able to discontinue	Bosman et al. (2016); Dickinson et al. (2010); Verbeek-Heida and Mathot (2006); Iden et al. (2011); Karp (1993); Knudsen et al. (2002); Eveleigh (2015); Gibson (2016); Schofield (2011); Leydon et al. (2007).	Psychological capability	Enablement; training; education	13.2 Framing/reframin g<br><br>15.2. Persuasion about capability	Knowledge, Self-efficacy (Mastery experiences vicarious experiences).<br><br>Social outcome expectations; Knowledge; physical outcome expectations	Cognitive participation: Initiation<br><br>Cognitive participation: Activation
	Successful stopping	Indicate that many people stop SD without problems, and those who are worried can overcome their concerns		Psychological capability	Enablement; training; education			
	Concerns about stopping	Patients will be given a selection of options to click on to read more about specific concerns		Psychological capability	Enablement; training; education			
	How will I cope if something big happens?	Reassure that AD-visor has guidance on managing stress in difficult		Psychological capability	Enablement; training; education			

		situations. Signpost to Moving Forward module.						
	What if I go back to how I was before?	Reassure that AD-visor has guidance on preventing relapse and signpost to Keeping Well module.		Psychological capability	Enablement; training; education			
	What if I have to start taking antidepressants again?	Reassure that hopefully this won't be necessary because they will learn how to prevent relapse, but if it is, they can try withdrawing again in future		Psychological capability	Enablement; training; education			
	How will I manage my responsibilities?	Guidance on planning activities and highlight the importance family support as well as the timing of the tapering process		Psychological capability	Enablement; training; education			

	Dealing with worries	Reflecting on the motivations to discontinue and weighing these up against concerns.		Reflexive motivation	Enablement; training; education			
**Keeping well**	Keeping well	Introduce to the idea of relapse prevention	Kuyken (2008); Allen (2009); Kuyken (2010); Fava (1998); Cromarty (2011); Otto (2010);	Psychological capability	Enablement; training; education	11.2 Reduce negative emotions<br><br>13.2 Framing/reframing<br><br>6.1 Demonstration of behaviour<br><br>4.3 Reattribution	Knowledge, Goals Self-efficacy (Mastery experiences vicarious experiences).<br><br>Social outcome expectations; Knowledge; physical outcome expectation	Cognitive participation: Activation
	Automatic pilot	Define running on autopilot and explain negative automatic thoughts		Psychological capability	Enablement; training; education			
	The power of thoughts	Explain how the way we think impacts mood and teach cognitive defusion (thoughts are not facts)		Psychological capability	Enablement; training; education			
	Let it be	Defining the term ‘acceptance’ and why it is useful in dealing		Psychological capability	Enablement; training; education			

		with difficult thoughts and feelings						
	Recognising warning signs	Explaining and reflecting on what thoughts and physical sensations might be indicators of relapse		Psychological capability	Enablement; training; education			
	Recognising triggers	Reflecting on situations that might trigger a relapse		Psychological capability	Enablement; training; education			
	Recognising relapse	Writing down warning signs and triggers and saving these to view later		Psychological capability	Enablement; training; education			
	Responding differently	Highlight that you cannot change thoughts or the things that happen in life, but you have a choice how to respond to these. Responding in more helpful		Psychological capability	Enablement; training; education			

		ways can prevent relapse.						
	Preventing relapse	1. Take a breath<br>2. Make a decision on how to act<br>3. Take action		Psychological capability	Enablement; training; education			
**Living life with values and goals***	What are values	Defines values as like a compass point providing direction for our lives.	Swain et al. 2013; Powers et al. 2009.	Psychological capability	Enablement; training; education	11.2 Reduce negative emotions<br><br>13.2 Framing/reframin g<br><br>6.1 Demonstration of behaviour	Knowledge, Goals	Coherence: Internalisation
	What do I value?	Provides a space to write down what they value		Psychological capability	Enablement; training; education			
	Goals	Explaining the need to set goals in order to		Psychological capability	Enablement; training; education			

		act in line with our values				4.3 Reattribution		
	Setting goals	Guidance and space to write goals		Psychological capability	Enablement; training; education			
	Meeting goals	Reminds users to revisit this section to review their goals and see if they have met them		Psychological capability	Enablement; training; education			
**Dealing with withdrawal symptoms**	What are withdrawal symptoms?	Describes what they are and that they are a consequence of the brain and body adapting to the change in medication	*Bosman et al. (2016); Dickinson et al. (2010); Verbeek-Heida and Mathot (2006); Iden et al. (2011); Karp (1993); Knudsen et al. (2002); Eveleigh (2015); Gibson (2016); Schofield (2011); Leydon et al. (2007)*	Psychological capability Physical capability	Enablement; training; education	13.2 Framing/reframin g<br><br>6.1 Demonstration of behaviour<br><br>4.3 Reattribution	Social outcome expectations; Knowledge; physical outcome expectations	Cognitive participation: Activation
	Recognisin g withdrawal symptoms	This page highlights that there are different symptoms that might be physical or mental. Specific details of what symptoms may occur are not given.		Psychological capability Physical capability	Enablement; training; education			

	Thinking about withdrawal symptoms	Explains that the way we think about symptoms can change how much impact they have (e.g. if you mistake a withdrawal symptom for relapse, it may be harder for the symptom to pass).		Psychological capability Physical capability	Enablement; training; education			
	Knowing the difference	Details about the differences between withdrawal symptoms and relapse.		Psychological capability Physical capability	Enablement; training; education			
	Dealing with withdrawal symptoms	Mild symptoms can be tolerated and will pass, moderate symptoms can be treated by a doctor, and severe symptoms may indicate a slower taper is needed.		Psychological capability Physical capability	Enablement; training; education			

	Accepting withdrawal symptoms	Guidance on accepting/tolerating symptoms based on acceptance and commitment exercises used with chronic physical symptoms		Psychological capability Physical capability	Enablement; training; education			
**Moving forward**	Healthy Paths Through Stress intervention (Healthy Paths). See Geraghty et al. 2017 for full description	This module is based on an intervention aimed at managing life stresses. The modules have been developed as part of a separate project and their content will be incorporated into AD-visor. This section will include guidance on mindfulness practices and behavioural activation.	Muñoz et al. 2005; Geraghty et al. 2016.	Psychological capability	Enablement; training; education	11.2 Reduce negative emotions<br><br>13.2 Framing/reframing<br><br>6.1 Demonstration of behaviour<br><br>4.3 Reattribution	Knowledge, Goals Self-efficacy (Mastery experiences vicarious experiences).<br><br>Social outcome expectations; Knowledge; physical outcome expectations	Coherence: Individual specification<br><br>Coherence: Internalisation<br><br>Cognitive participation: Initiation<br><br>Cognitive participation: Activation

New references added:

Bosman, R. C., Huijbregts, K. M., Verhaak, P. F. M., Ruhé, H. G., Van Marwijk, H. W. J., Van Balkom, A. J. L. M., & Batelaan, N. M. (2016). Long-term antidepressant use: A qualitative study on perspectives of patients and GPs in primary care. *British Journal of General Practice*, *66*(651), e708–e719. https://doi.org/10.3399/bjgp16X686641

Cartwright, C., Gibson, K., & Read, J. (2016). Long-term antidepressant use : patient perspectives of benefits and adverse effects, 1401–1407. https://doi.org/10.2147/PPA.S110632

Cromarty, P., Jonsson, J., Moorhead, S., & Freeston, M. H. (2011). Cognitive Behaviour Therapy for Withdrawal from Antidepressant Medication: A Single Case Series. *Behavioural and Cognitive Psychotherapy*, *39*(01), 77–97. https://doi.org/10.1017/S1352465810000512

Dickinson, R., Knapp, P., House, A. O., Dimri, V., Zermansky, A., Petty, D., … Raynor, D. K. (2010). Long-term prescribing of antidepressants in the older population: A qualitative study. *British Journal of General Practice*, *60*(573), 257–259. https://doi.org/10.3399/bjgp10X483913

Eveleigh, R. H. (2015). *Inappropriate long-term antidepressant use in primary care: A challenge to change*. Radboud University Nijmegen. https://doi.org/10.1063/1.3033202

Fava, G. A., Rafanelli, C., Grandi, S., Conti, S., & Belluardo, P. (1998). Prevention of Recurrent Depression With Cognitive Behavioral Therapy. *Arch Gen Psychiatry*, *55*, 816–820.

Geraghty, A. W. A., Muñoz, R. F., Yardley, L., Mc Sharry, J., Little, P., & Moore, M. (2016). Developing an Unguided Internet-Delivered Intervention for Emotional Distress in Primary Care Patients: Applying Common Factor and Person-Based Approaches. *JMIR Ment Health*, *3*(4), e53.

Gibson, K., Cartwright, C., & Read, J. (2016). "In my life antidepressants have been..": A qualitative analysis of users' diverse experiences with antidepressants. *BMC Psychiatry*, *16*(1), 1–7. https://doi.org/10.1186/s12888-016-0844-3

Grime, K. P. J. (2002). Understanding Depression and its Treatment: GP, Patient and Pharmacist Perspectives, (September), 52–60. Retrieved from http://www.pharmacyresearchuk.org/waterway/wp-content/uploads/2012/11/Understanding_depression_and_its_treatment1.pdf

Iden, K. R., Hjørleifsson, S., & Ruths, S. (2011). Treatment decisions on antidepressants in nursing homes: A qualitative study. *Scandinavian Journal of Primary Health Care*, *29*(4), 252–256. https://doi.org/10.3109/02813432.2011.628240

Karp, D. A. (1993). Taking anti-depressant medications: Resistance, trial commitment, conversion, disenchantment. *Qualitative Sociology*, *16*(4), 337–359. https://doi.org/10.1007/BF00989969

Knudsen, P., Hansen, E. H., Traulsen, J. M., & Eskildsen, K. (2002). Changes in Self-Concept While Using SSRI Antidepressants. *Qualitative Health Research*, *12*(7), 932–944. https://doi.org/10.1177/104973202129120368

Kuyken, W., Byford, S., Watkins, E., Holden, E., White, K., King 's, B. B., … Teasdale, J. D. (2008). Mindfulness-Based Cognitive Therapy to Prevent Relapse in Recurrent Depression. *Journal of Consulting and Clinical Psychology*, *76*(6), 966–978. https://doi.org/10.1037/a0013786

Kuyken, W., Watkins, E., Holden, E., White, K., Taylor, R. S., Byford, S., … Dalgleish, T. (2010). How does mindfulness-based cognitive therapy work? *Behaviour Research and Therapy*, *48*, 1105–1112. https://doi.org/10.1016/j.brat.2010.08.003

Leydon, G. M., Rodgers, L., & Kendrick, T. (2007). A qualitative study of patient views on discontinuing long-term selective serotonin reuptake inhibitors. *Family Practice*, *24*(6), 570–575. https://doi.org/10.1093/fampra/cmm069

Muñoz, R. F., & Mendelson, T. (2005). Toward evidence-based interventions for diverse populations: The San Francisco General Hospital prevention and treatment manuals. *J Consult Clin Psychol*, *73*(5), 790-799.

Otto, M. W., McHugh, R. K., Simon, N. M., Farach, F. J., Worthington, J. J., & Pollack, M. H. (2010). Efficacy of CBT for benzodiazepine discontinuation in patients with panic disorder: Further evaluation. *Behaviour Research and Therapy*, *48*(8), 720–727. https://doi.org/10.1016/J.BRAT.2010.04.002

Powers, M. B., Zum Vorde Sive Vording, M. B., & Emmelkamp, P. M. (2009). Acceptance and commitment therapy: a meta-analytic review. *Psychother Psychosom*, *78*(2), 73-80.

Schofield, P., Crosland, A., Waheed, W., Aseem, S., Gask, L., Wallace, A., … Tylee, A. (2011). Patients' views of antidepressants: From first experiences to becoming expert. *British Journal of General Practice*, *61*(585), 142–148. https://doi.org/10.3399/bjgp11X567045

Swain, J., Hancock, K., Hainsworth, C., & Bowman, J. (2013). Acceptance and commitment therapy in the treatment of anxiety: a systematic review. *Clin Psychol Rev*, *33*(8), 965-978.

Verbeek-Heida, P. M., & Mathot, E. F. (2006). Better safe than sorry — why patients prefer to stop using selective serotonin reuptake inhibitor (SSRI) antidepressants but are afraid to do so: results of a qualitative study. *Chronic Illness*, *2*(2), 133–142. https://doi.org/10.1179/174592006X111003
