# Supplementary material for: Supporting antidepressant discontinuation: the development and optimisation of a digital intervention for patients in UK primary care using a theory, evidence and person-based approach
Source: BMJ Open. 2020 Mar 8;10(3):e032312. doi: 10.1136/bmjopen-2019-032312 (PMC7064123; doi:10.1136/bmjopen-2019-032312)
Supplement: Author's manuscript [file bmjopen-2019-032312.draft_revisions.pdf]

BMJ Open is committed to open peer review. As part of this commitment we make the peer review history of every article we publish publicly available.

When an article is published we post the peer reviewers' comments and the authors' responses online. We also post the versions of the paper that were used during peer review. These are the versions that the peer review comments apply to.

The versions of the paper that follow are the versions that were submitted during the peer review process. They are not the versions of record or the final published versions. They should not be cited or distributed as the published version of this manuscript.

BMJ Open is an open access journal and the full, final, typeset and author-corrected version of record of the manuscript is available on our site with no access controls, subscription charges or pay-per-view fees (http://bmjopen.bmj.com).

If you have any questions on BMJ Open's open peer review process please email info.bmjopen@bmj.com

BMJ Open

Supporting antidepressant discontinuation: The development and optimisation of a digital intervention for patients

Journal:	*BMJ Open*
Manuscript ID	bmjopen-2019-032312
Article Type:	Research
Date Submitted by the Author:	12-Jun-2019
Complete List of Authors:	Bowers, Hannah; University of Southampton Faculty of Medicine, Primary Care and Population Sciences<br>Kendrick, Tony; University of Southampton, Primary Care and Population Sciences<br>Glowacka, Marta; Bournemouth University, Faculty of Health and Social Sciences<br>Williams, Samantha; University of Southampton, Primary Care & Population Sciences<br>Leydon, Geraldine; University of Southampton, Primary Care and Population Sciences<br>May, Carl; London School of Hygiene and Tropical Medicine Faculty of Epidemiology and Population Health<br>Dowrick, CF; University of Liverpool,<br>Moncrieff, Joanna; University College London and North East London mental health trust, Mental Health Sciences<br>Laine, Rebecca; University of Southampton, Primary Care and Population Sciences<br>Nestoriuc, Yvonne; Helmet Schmidt University, Department of Psychology<br>Andersson, Gerhard; Linkoping University; Karolinska Institutet, Department of Clinical Neuroscience and Psychiatry<br>Geraghty, Adam; University of Southampton, Primary Care and Population Sciences
Keywords:	Depression & mood disorders < PSYCHIATRY, Intervention Development, Digital Intervention, Antidepressants, PRIMARY CARE

SCHOLARONE™
Manuscripts

I, the Submitting Author has the right to grant and does grant on behalf of all authors of the Work (as defined in the below author licence), an exclusive licence and/or a non-exclusive licence for contributions from authors who are: i) UK Crown employees; ii) where BMJ has agreed a CC-BY licence shall apply, and/or iii) in accordance with the terms applicable for US Federal Government officers or employees acting as part of their official duties; on a worldwide, perpetual, irrevocable, royalty-free basis to BMJ Publishing Group Ltd ("BMJ") its licensees and where the relevant Journal is co-owned by BMJ to the co-owners of the Journal, to publish the Work in this journal and any other BMJ products and to exploit all rights, as set out in our licence.

The Submitting Author accepts and understands that any supply made under these terms is made by BMJ to the Submitting Author unless you are acting as an employee on behalf of your employer or a postgraduate student of an affiliated institution which is paying any applicable article publishing charge ("APC") for Open Access articles. Where the Submitting Author wishes to make the Work available on an Open Access basis (and intends to pay the relevant APC), the terms of reuse of such Open Access shall be governed by a Creative Commons licence – details of these licences and which Creative Commons licence will apply to this Work are set out in our licence referred to above.

Other than as permitted in any relevant BMJ Author's Self Archiving Policies, I confirm this Work has not been accepted for publication elsewhere, is not being considered for publication elsewhere and does not duplicate material already published. I confirm all authors consent to publication of this Work and authorise the granting of this licence.

For peer review only

Digital intervention for antidepressant discontinuation

Supporting antidepressant discontinuation: The development and optimisation of a digital intervention for patients

Hannah M Bowers*[1], Tony Kendrick[1], Marta Glowacka[2], Samantha Williams[1], Geraldine Leydon[1], Carl May[3], Chris Dowrick[4], Joanna Moncrieff[5], Rebecca Laine[1], Yvonne Nestoriuc[6], Gerhard Andersson[7,8], Adam W A Geraghty[1].

[1] Primary Care and Population Sciences, University of Southampton, UK.

[2] Faculty of Health and Social Sciences, Bournemouth University, UK.

[3] Department of Health Services Research and Policy, London School of Hygiene and Tropical Medicine, UK

[4] Institute of Population Health Sciences, University of Liverpool, UK.

[5] Division of Psychiatry, University College London, UK.

[6] Department of Psychology, Helmut Schmidt University, Germany.

[7] Department of Behavioural Sciences and Learning, Linkoping University, Sweden.

[8] Department of Clinical Neuroscience and Psychiatry, Karolinska Institutet, Sweden.

…

For peer review only

Digital intervention for antidepressant discontinuation

*Corresponding Author: H.M.Bowers@soton.ac.uk. Primary Care and Population Sciences, University of Southampton, Aldermoor Health Centre, Aldermoor Close, Southampton, SO15 5HT. Tel: 02380 591743

…

For peer review only

Abstract

Objectives: We aimed to develop a digital intervention to support antidepressant discontinuation in UK primary care. In this paper we describe the development using a theory- evidence- and person-based approach.

Design: Intervention development using a theory-, evidence-, and person-based approach

Setting: Primary Care in the South of England

Participants: Fifteen participants with a range of antidepressant experience took part in 'think aloud' interviews for intervention optimisation

Intervention: Our digital intervention prototype (called 'ADvisor') was developed on the basis of a planning phase consisting of qualitative and quantitative reviews, an in-depth qualitative study, the development of guiding principles and a theory-based behavioural analysis. Our optimisation phase consisted of 'think aloud' interviews where the intervention was iteratively refined.

Results: The qualitative systematic review and in-depth qualitative study highlighted the centrality of fear of depression relapse as a key barrier to discontinuation. The quantitative systematic review showed that psychologically informed approaches such as cognitive behaviour therapy (CBT) were associated with greater rates of discontinuation than simple advice to reduce. Following a behavioural diagnosis based on the Behaviour Change Wheel,

Digital intervention for antidepressant discontinuation

Social Cognitive Theory provided a theoretical basis for the intervention. The intervention was optimised on the basis of think aloud interviews, where participants suggested they like the flexibility of the system and found it reassuring. Changes were made to the tone of the material and the structure was adjusted based on this qualitative feedback.

Conclusions: 'ADvisor' is an evidence-, theory- and person-based digital intervention designed to support antidepressant discontinuation. The intervention was perceived as helpful and reassuring in optimisation interviews. Trials are now needed to determine the feasibility, clinical and cost effectiveness of this approach.

271 word (BMJOpen limit 300).

For peer review only

Digital intervention for antidepressant discontinuation

Strengths and Limitations of the study

- A systematic review and qualitative meta-synthesis were conducted alongside primary qualitative work to guide the content of the intervention.
- A theory-based behavioural analysis and the development of guiding principles further informed the planning phase of intervention development.
- Think aloud interviews provided in-depth understanding of patients' views of the intervention in terms of usability and content.
- The intervention was iteratively refined throughout the think aloud interviews to produce an intervention that aligns with patient preference.
- Think aloud participants were predominantly White British and from more affluent regions in the South of England and may not represent the views of all antidepressant users.

For peer review only

Digital intervention for antidepressant discontinuation

Introduction

The number of antidepressant prescriptions in the UK has continued to rise over the past four decades [1], a trend which has also been seen in the United States and across Europe [2,3]. Approximately 10% of adults in the UK are currently prescribed antidepressant medication [4]. Though antidepressants can prevent relapse, there is evidence that 30-50% of patients on long-term antidepressants have no indication based on guidelines for long-term use [5–7]. Research suggests this increase in prescribing is primarily due to general practitioners (GPs) prescribing antidepressants for longer and longer durations over time [8]. Long-term antidepressant use is both costly to the UK National Health Service (NHS) (in terms of prescription and appointment costs) and is associated with increased side effects [9]. Attempting to discontinue antidepressants in the 30-50% with no indication for long-term use may therefore be beneficial to patients and positively impact on use of health-care resources.

There are many factors that may contribute to long-term antidepressant use, including the occurrence of a physiological withdrawal syndrome following reduction or cessation and psychological factors such as beliefs about the necessity of long-term use and fear of relapse [10]. Infrequent reviews of patients taking antidepressants may also contribute to sustained use [11]. However, simply prompting for patient reviews has resulted in discontinuation rates of 6-8%, not

For peer review only

significantly differing from usual care [12,13]. This highlights the potential importance of psychologically informed interventions to support withdrawal.

Trials have shown that Cognitive Behavioural Therapy (CBT) and Mindfulness-Based Cognitive Therapy (MBCT) can effectively support discontinuation of antidepressants, with cessation rates ranging from between 55%-95% [14–18]. Although producing positive outcomes, these interventions involve intensive group/face-to-face courses, thus access and ability to scale up within resource-strapped health services may be severely limited. There is a need for accessible, scalable psychologically-informed interventions that can effectively support individuals where discontinuation is appropriate.

In the UK, 89% of the general population in 2018 used the internet weekly, up from 55% in 2006 [19]. Internet-based digital interventions supported with human contact have been shown to effectively reduce depression and anxiety [20]. Digital intervention may have potential to provide a scalable, accessible way of supporting appropriate antidepressant discontinuation. We aimed to develop such a supported digital intervention as part of the UK-based REDUCE (REviewing long term antiDepressant Use by Careful monitoring in Everyday practice) programme to develop and trial safe, feasible and effective ways to support patients withdrawing from antidepressants where appropriate. In this paper we describe the planning and

optimisation of our patient-facing digital intervention to support discontinuation, named 'ADvisor'.

Phase 1: Intervention planning and development

Methods

There is a range of systematic protocols for intervention development that can be drawn on at the outset of a development project (e.g. Intervention Mapping [21]). We chose to implement a theory-, evidence- and person-based approach [22]. This comprehensive strategy integrates the person-based approach (PBA) [23,24] with more commonly used theory and evidenced-based methods. The PBA provides guidance for integrating systematic in-depth qualitative research into the development process. Drawing on the PBA ensures evidence and theory-based techniques are applied with a full understanding of the target users' perspectives and psychosocial context [23]. We will outline the components of our comprehensive approach including systematic reviewing, primary qualitative research, development of guiding principles, behavioural analysis and logic modelling.

Systematic reviewing

Two systematic reviews were conducted: a quantitative review with meta-analysis, and a qualitative thematic synthesis, described in detail elsewhere [10,25]. For

For peer review only

intervention planning, from the quantitative review we drew out interventions that had successfully supported discontinuation and considered their intervention components, seeking full manuals where possible. We aimed to determine which components could be best translated into a digital format. In the qualitative review we identified barriers and facilitators to antidepressant discontinuation. Barriers and facilitators were tabulated and used to inform the 'Guiding Principles' (see below) as well as content for the intervention.

Primary qualitative research

Individual semi-structured interviews were conducted by SW with primary care patients with varying experiences of antidepressants, and varying levels of motivation to stop. These interviews explored patients' views on barriers and facilitators to withdrawal, the role of health care professionals in supporting withdrawal attempts, and elements of a proposed intervention to support withdrawal. Interviews were conducted at the patients' homes or their GP practices and were audio recorded and transcribed verbatim. Analysis was conducted following thematic analytic principles suggested by Braun and Clarke [26], and Joffe and Yardley [27]. Analysis was conducted by SW (a qualitative researcher). The coding manual and developed themes were discussed and agreed by the wider development group.

Development of guiding principles

For peer review only

Digital intervention for antidepressant discontinuation

Guiding principles are a fundamental part of the PBA [23]. They represent broad design objectives that guide the application/implementation of the core intervention strategies, aiming to increase engagement [24]. Guiding principles were developed based on the qualitative synthesis [10] and primary qualitative findings. Through this qualitative work we aimed to identify key behavioural needs, challenges or issues the intervention needed to address.

Behavioural analysis

Behavioural and implementation theory was drawn on as we triangulated between the qualitative and quantitative evidence, and the expert views of our team (including patient representatives, GPs, psychiatrists, psychologists, sociologists and health services researchers) to determine important intervention components. Using the Behaviour Change Wheel and COM-B model of behavior (Capability, Opportunity, Motivation – Behaviour) [28], informed by our qualitative research, we conducted a 'behavioural diagnosis' [29]. In behavioural diagnosis, factors that are likely to affect the central target behaviour are considered in terms of capability, opportunity, and motivation [28,29]. Once we had proposed initial intervention content/components, these were mapped theoretically using the Behaviour Change Wheel, Social Cognitive Theory (SCT) [30] and Normalisation Process Theory [31]. As well as providing a mapped full description of the proposed intervention, this process ensured we did not miss areas of theory that may have improved the intervention.

For peer review only

Results

Systematic reviewing

Our qualitative thematic synthesis (see [10] for full results) across 22 studies highlighted key barriers and facilitators to discontinuation. Patients' concerns regarding their ability to cope and psychological dependence were common barriers, as were difficulties experienced in previous stopping attempts. Confidence in abilities to stop, effective coping strategies and stable life circumstances facilitated discontinuation. Additional important themes included fear of relapse – this was the central fear that prohibited stopping attempts – and beliefs about depression. The belief that depression was a long-term condition caused by biochemical changes in the brain was a key barrier to discontinuation. Where patients reported a very different belief, that depression was due to changing life circumstances, this seemed to facilitate discontinuation. Patients' self-identity and goals were an important factor: Having self-identifying as "old" or "disabled" acted as a barrier to discontinuation, and having goals to function independently functioned as facilitator to discontinuation.

In the quantitative systematic review (see [25] for full results) a variety of therapeutic techniques were implemented including a patient-specific letter to the GP with a recommendation to discontinue plus tapering advice; GP review of the patient's

For peer review only

condition and medication; CBT plus tapering; MBCT with tapering support gradual discontinuation and one-week tapering. The results indicated that CBT or MBCT plus tapering are helpful for patients discontinuing antidepressants, with cessation rates of 40-95% [23], compared to only 6-8% cessation where health professionals are simply prompted to review patients. CBT plus tapering resulted in lower relapse rates compared with clinical management plus taper (15-25% vs 35-80%) [23]. The content of the interventions were extracted and feasibility of delivery in a digital format was considered. We developed a module based closely on MBCT protocols on the basis of this review.

The findings from both reviews' findings informed the guiding principles, behavioural analysis and logic model, which formed the basis for intervention content selection and development.

Primary qualitative research

Five themes were developed through the thematic analysis of 19 patient interviews (full details will be published elsewhere). A summary is presented here. Participants spoke of the centrality of personal medication and health care factors, for example some patients described the need for a personalised tapering regime to support them discontinuing. Beliefs about depression and its treatment were key in shaping participants' stance towards discontinuing. For example, ideas around the necessity of anti-depressant medication due to 'chemical imbalance' were common. Holding

For peer review only

these beliefs made patients less likely to consider stopping. Fear of stopping, driven by fear of relapse were discussed as central barriers to withdrawal. The impact of others also appeared to be important. For example, the perception of stigma and the feeling of letting people down, made participants less willing to discontinue, while having a good support network was considered beneficial to stopping. Participants were also asked to consider digital methods of intervention delivery. Elements participants wanted to see in the intervention included explanation around how antidepressants work, support for anxiety/fear of discontinuing, coping strategies and information on withdrawal symptoms. There was some concern around privacy and around preference for greater face-to-face interaction to support them during the discontinuation phase. Patients expressed a need to have accessible, interactive and information presented in an aesthetically pleasing way.

The full findings in our primary qualitative research mirrored and expanded the findings of our qualitative thematic synthesis. They fed into the guiding principles, behavioural analysis logic model and content for the intervention.

Guiding principles

On the basis of the qualitative work guiding principles were developed (comprised of design objectives and design features), see Table 1. We developed two broad design objectives: The first, regarding building confidence that discontinuing antidepressant medication is safe and achievable, was developed from prominent

For peer review only

themes around fear of stopping, the need for confidence, and beliefs that antidepressant medications are needed long-term. The second objective, that the intervention should be an accessible, motivating resource that supports patients in managing their withdrawal in a manner that aligns with their preferences, was developed in response to the range of views and beliefs held about the nature of depression and why antidepressants were necessary. Design features that support both these objectives are listed in Table 1.

[Insert table 1 about here]

Behavioural analysis

Our behavioural diagnosis following the COM-B model can be found in Appendix A. Our target behaviour was reducing and stopping the taking of antidepressant medication. Based on our reviews, qualitative work and discussion amongst our broader team, psychological capability and reflective motivation were considered key constructs for changing the target behaviour. Psychological capability involves having the necessary knowledge and psychological skills to engage with the target behaviour. For antidepressant discontinuation, increasing psychological capacity would involve improving knowledge about the withdrawal process including expectations and practicalities; and developing important psychological skills including: helpful appraisals of symptoms; relapse prevention; and stress management. Reflective motivation includes reflective processes involving

For peer review only

evaluations and beliefs along with self-conscious intentions. For antidepressant discontinuation this would mean working to modify beliefs about depression, for example, to challenge the belief that depression is a life-long brain condition for which antidepressants will always be required. We would also aim to modify beliefs about treatment that may work as barriers to withdrawal, for example, that withdrawal is always challenging and unachievable.

Following the drafting of module content and structure, we mapped content against 1) studies suggesting content would be important, 2) Behaviour Change Wheel (BCW) constructs, 3) Social Cognitive Theory (SCT), and 4) Normalisation Process Theory (NPT). See Appendix B. In the introductory module, for example, the key BCW functions that were used were enablement, training, education and persuasion; SCT constructs included outcome expectations (social and physical) and self-efficacy; NPT constructs included 'coherence: individual specification' (sense making work that individuals do when beginning to operationalise a set of proposed practices) and 'cognitive participation: initiation' (willingness to engage in new processes).

Fundamentally, SCT [32] underlies the approach taken in the intervention to facilitate behaviour change. The intervention is designed to increase self-efficacy for stopping and to modify outcome expectations e.g. increase positive expectation that the recommended strategies are likely to support effective discontinuation. At a later

For peer review only

stage in development, the Necessity Concerns Framework (NCF) [33] was considered. NCF was developed to explain the role of treatment beliefs on adherence behaviours. According to NCF, adherence to treatment is a function of patients' beliefs about the necessity of their medication and the concerns they have about it; high necessity beliefs and low concerns are likely to predict medication adherence [34]. In the context of antidepressant withdrawal, accordingly, we would need to reduce patients' beliefs about the necessity of the medication, highlight likely benefits of stopping, and reduce concern regarding the stopping process. All of these factors will ultimately impact on self-efficacy, hence the centrality of SCT in our theoretical modelling.

Logic modelling

Logic models represent proposed or hypothesised 'theories of change' outlining the problem/issue and barriers, ingredients mechanism, and how these may affect target outcomes [35]. We developed a draft logic model for the REDUCE patient intervention, drawing on theory, evidence and our person-based qualitative work, see Figure 1.

[Insert Figure 1 about here]

Outline intervention content

For peer review only

Digital intervention for antidepressant discontinuation

On the basis of our planning process, a prototype digital intervention was developed for patients taking antidepressants long-term (defined as more than one year for a first episode or more than two years following two or more episodes). The contents of the online intervention are described in Table 2. A digital intervention for health professionals (providing information and guidance on antidepressant reduction) was also developed as part of the REDUCE programme and is reported separately.

[Insert Table 2 about here]

Content was developed using findings from the reviews of the literature, primary qualitative work, behavioural analysis and logic modelling. In addition to online content, scheduled telephone support contacts with specialists trained in providing psychological support and email reminders were developed as part of the patient intervention.

When accessing the ADvisor intervention for the first time, users view a core module with the central rationale for stopping antidepressants; they can then access a menu with a range of further modules based on our planning work. Aligning with our guiding principles, users are advised that they can use ADvisor how and when they would like. It is their tool, to be used to support them in a way that is consistent with their needs, preferences and experience. Through this approach we aimed to maximise autonomous motivation [36].

For peer review only

Digital intervention for antidepressant discontinuation

Content for the online intervention was initially drafted by a member of the content development team (HB) before AG and MG and then wider team members offered their expertise and informed further development of the content. This iterative process continued until all team members were satisfied that the prototype intervention addressed key experiences, barriers and facilitators identified by the work from phase one and were in line with the guiding principles, theoretical modelling and logic model. The content was transferred into online pages in LifeGuide (www.lifeguideonline.org) and further amendments to the presentation were made by the team before moving forward to the optimisation phase.

For peer review only

Phase 2: Intervention optimisation

Methods

Design

Within the PBA, 'think aloud' qualitative studies are employed to optimise the prototype intervention. Think aloud studies are designed to elicit in-depth perspectives about the nature of the content, rather than solely focusing on functionality and usability.

Digital intervention for antidepressant discontinuation

Participants

Participants were recruited from eight primary care practices in the South of England. Eligibility criteria were as follows: Inclusion criteria: Taking antidepressants for more than one year for a first episode or two years for a subsequent episode; discontinued antidepressants, or were in the process of tapering. Exclusion criteria: PHQ-9 scores greater than 10 (suggesting persisting symptoms of depression) and those who reported any suicide ideation; history of suicide attempts; ongoing social difficulties or recent life events likely to provoke relapse; more than three previous significant episodes of depression; comorbid psychosis, bipolar disorder, obsessive-compulsive disorder, or substance use (or past history of these conditions); or currently receiving psychiatric treatment.

Procedure

Eligible participants met with a researcher (HB, SW or TK) either in their own home or at their primary care practice to take part in a think-aloud interview. Interviews invited participants to engage with the prototype intervention and say what they were thinking, aloud in real time. The interviewer prompted participants when necessary (for example asking patients 'How do you feel about the information on this page?'). Interviews ranged from 38 to 93 minutes in length and were audio recorded, and transcribed verbatim. The interview schedule can be found in Appendix C. There were three primary iterations of interviews based on three key modified prototype interventions. Patients at the start of the study therefore saw different versions of the

For peer review only

intervention to those who were recruited later rounds. This allowed the changes made as a result of patient feedback to continue to be tested. Interviews with patients continued until data saturation was reached, defined here as when comments about the intervention reflected that no further changes were necessary and there were therefore no new codes identified.

Analysis

Transcribed interviews were analysed using two primary analytic methods. The first analytic method was a more rapid coding than thematic analysis, which involves using coding tables designed for the PBA, where positive and negative comments were tabulated. Core problematic issues likely to affect participant engagement or intervention effectiveness identified using this coding method were brought to the broader group, and amendments to the intervention agreed. Alongside this method, a more in-depth thematic analysis [26,27] was developed to capture patient views of the intervention and ideas about how they might engage with it, beyond comments on what might be amended. For this latter analysis, HB independently coded the transcripts and discussed a preliminary coding frame with a second researcher (AG). Theme labelling and interpretation were discussed and agreed by the team. The thematic analysis is presented here.

Results

For peer review only

Of the 42 patients who returned a postal reply slip expressing interest, 11 were ineligible, nine could not subsequently be contacted, two later declined, and five expressed an interest only after data saturation had been reached. This resulted in a final sample of 15 patients (see Table 3 for sample characteristics).

[Insert Table 3 about here]

For peer review only

Findings

Six themes were developed, namely: flexible use; familiarity with content; reassurance; utility of information; teaching of useful skills; and feeling supported. Patient identifiers and demographic information are presented below each quote, where round number refers to the iteration of the intervention that the patient saw.

Flexible use

Participants discussed how ADvisor could be used in different ways to suit the individual. When viewing the main menu page in ADvisor participants talked about how different sections would be more useful for them, and that some sections were not relevant for them at that particular time.

> *Dealing with withdrawal symptoms, I don't have any, so it's fine. That [keeping well and moving forward modules] I'm more interested in about because I think*

that's - for me, keeping well and moving forward is where I am and where I want to be.

[14/03/0001] [round 1] [female] [36]

Initial versions of the intervention included an introduction module within which participants could choose which of two options they would like to view first, though they would need to view both sections before moving onto the main menu. Some participants felt that this was in contradiction to the aim of choice and flexibility. We therefore modified the intervention so that the introduction was shorter and these two choices were moved to optional buttons in the main menu.

It's kind of saying you've really got to look at that one; otherwise, you will have flicked back through or I would have thought it might have been, if it's really flexible, user friendly, you might be allowed to skip that page because you could always revisit it again.

[01/01/0026] [round 1] [male] [64]

Participants not only varied in the topics they wanted to look at, but also in terms of the different exercises they would choose to engage with in ADvisor. Some participants liked the idea of writing down their responses in ADvisor while others did not.

For peer review only

Digital intervention for antidepressant discontinuation

No. That's me. No, I'm very stoic and – just – I don't need to write it down, it's fine; I know what I'm doing, I'm fine, very much, I think.

[01/01/0005] [round 2] [male] [35]

I'd like to say that I would [write things down]; I think I probably would if I was – you know – really serious about it, because I like to write things down and if I haven't written it down, it can just go out of my brain. So I think, for me, it would be important to write that down.

[05/01/0022] [round 2] [female] [59]

Participants also discussed how ADvisor could be used in different ways. For example, it can be something used regularly, something one can pick up as and when necessary or it can be read through all in one go.

So it looks like you can use it when you want to but if you feel you're coping without, so it's not something you have to do all the time.

[05/01/0022] [round 2] [female] [59]

Yes, I would use it for future reference, as well, because you can always go backwards, can't you? With anything, I mean. If I ever came to a time where I was feeling down, I think, to go back on to something is to remind you. Because it's easy to forget.

For peer review only

[13/01/0058] [round 3] [female] [62]

Familiarity with content

Many of the participants referred to previous experience with psychological therapies or tools they have used in the past for their symptoms of depression. When reading cognitive-behavioural, acceptance and commitment, or mindfulness-based information in ADvisor, participants expressed a sense of familiarity with the terminology or messages they were presented.

Clicking on Breathing Space; that's very much mindfulness, isn't it? Yes, I like that, that's nice.

[14/03/0001] [round 1] [female] [36]

Some of the information about depression and antidepressants seemed to be obvious to a small number of participants who had pre-existing knowledge, but they understood that not all patients would have the same prior knowledge. One participant in particular who worked in healthcare found that much of the information was not new to her.

I'm obviously interested in reducing still further or coming off the antidepressants. ... See I don't think I can – I do know an awful lot about it and

For peer review only

read a lot about it and very – sorry – but, you know, being in the business myself, it's all a bit Noddy to Big Ears.

[13/01/0033] [round 3] [female] [64] [works in healthcare]

Reassurance

Participants described a sense of fear around stopping antidepressants. This has been reported in previous qualitative studies of patient and health professional perspectives on stopping [10]. Participants in this study often reported feeling reassured by information in ADvisor. While participants differed in terms of which particular piece of information they found reassuring, some participants noted feeling reassured knowing that they could go back on their antidepressant if they felt necessary. Other participants found that knowing that withdrawal symptoms are often short-lived offered reassurance.

Well that's a good section because that is quite a worry, I think, for anybody wanting to come off them; it would worry me what would my side-effects be and how would I feel coming off them. So to actually – I mean I didn't know this – to actually say that they are often short lived and go away in a few days or weeks is quite encouraging, isn't it.

[04/01/0025] [round 3] [female] [59]

Digital intervention for antidepressant discontinuation

As fear of withdrawal symptoms was highlighted in the qualitative work, withdrawal symptoms were discussed at several points during the introduction module. However participants who were not initially concerned about withdrawal symptoms felt that this was setting an expectation for difficulty withdrawing. Whilst not minimising withdrawal-related problems, we therefore revised the language around concerns about withdrawal in the introduction.

Well it's very obvious withdrawal is a problem, looking at all the advice you can see to help you get over it, which – yes. There's a negative feeling there, if it's stressed to this degree on this program, then you're obviously expecting trouble.

[10/03/0003] [male] [86]

Credibility of the information appeared to be important for participants. Participants liked to see the evidence base that was provided in ADvisor and in particular liked that it would be used within an NHS setting. The NHS affiliation seemed to provide a sense of reliability and credibility.

I'd be really pleased if they [GP/nurse] referred me to a website, especially if it was from the GP, because I think, well, it's backed up or supported by them.

[14/03/0001] [round 1] [female] [36]

There was a balance that needed to be struck between portraying information as credible and maintaining a warm and friendly tone. Participants reported some of the information in ADvisor as sounding academic and reading like it could be used by practitioners. As a result, the tone was revised to be warmer and friendlier, while maintaining a sense of credibility.

> *It's just very business-like so very much like maybe something that a university would produce or maybe that a medical professional would share amongst themselves and your everyday person who's maybe not used to reading things in so much detail any more, sadly. It's quite dry.*
>
> *[14/03/0001] [round 1] [female] [36]*

Utility of information

Participants described the information on withdrawal symptoms to be useful, in particular, some participants liked the information on how to distinguish between signs of relapse and withdrawal symptoms. One participant in particular expressed a shift in her views on discontinuing as a result of the information in ADvisor. She explained that had she known that withdrawal symptoms may feel like relapse and will pass, she may have persisted with her lower dose of antidepressant for longer. She also highlighted that difficulty in getting a GP appointment is a barrier for her to persist with discontinuing in the face of difficulties.

For peer review only

.. I didn't know ... withdrawal symptoms might appear the same as the symptoms that led to needing antidepressants in the first place, but they will pass after a short time; I didn't know that. I thought if you started feeling down again, then you were heading for a crash.

[13/03/0001] [round 2] [female] [47]

Some participants described wanting more detailed information about what withdrawal symptoms might be expected. However, upon discussion with the broader study team, it was decided to avoid setting expectations around particular symptoms as this may lead patients to experience expected symptoms. Patients can instead request this information from their GP if it is something they feel they would rather know about.

Participants also noted that it was useful to reflect on the side effects of taking antidepressants. There was an awareness that these can be hard to recognise, and three participants reported that after reading the information in ADvisor, they may in fact have been experiencing side effects of which they were previously unaware. One participant described how this made him even more inclined to discontinue.

Well, as I look at these, I think maybe I'm wrong; maybe I am still getting side-effects, but I've just learned to accept them or – I'm just a little bit in denial and

For peer review only

it makes me want to get off them even more, because then – lots of these things will, you know, will disappear.

[12/03/0003] [round 1] [male] [38]

Teaching of useful skills

Participants reported the skills included in ADvisor as being useful. In particular, advice around preventing relapse and mindfulness-based skills were considered to be useful.

Your triggers, recognising your emotions and reminding yourself that you don't have to react in a certain way; you can react in a different way. Yes, I think it's very good.

[13/01/0001] [round 2] [female] [47]

Acceptance of difficulties and of emotions was discussed as a useful coping strategy by participants, both with regards to their own pre-existing relationship to their emotions, and with regards to the messages in ADvisor on acceptance.

When you read it like that, it is true; the more you worry about things, the more down you get. So you've got to learn to stop doing that. I have to start putting that into practice if I'm going to do this.

For peer review only

[13/01/0058] [round 3] [female] [62]

Participants liked having tools and techniques in ADvisor for dealing with difficult emotions and life stresses. There was an understanding that life stress is often unavoidable, and participants expressed a desire to learn ways of dealing with stresses. Some participants stated that learning how to manage emotions would act as a replacement for taking antidepressants.

> *I think that exercise of sitting by the stream is very good, because I know when I had Cognitive Behavioural Therapy I was taught to – you know – when your thoughts came – to – and I still do this now – is always remember – say to yourself that it will pass, those feelings will pass and it might be horrible while you're going through those feelings, but find somewhere nice and comfortable to sit, with a blanket even, and that sort of thing.*

[04/01/0025] [round 3] [female] [59]

By the final interviews in the final round, participants' comments were positive with no new issues being identified. This signified the intervention was now ready for further evaluation and feedback in the planned feasibility trial to follow.

Discussion

For peer review only

Digital intervention for antidepressant discontinuation

We developed a digital intervention to support appropriate antidepressant discontinuation. The intervention was developed through a process of triangulation between quantitative and qualitative review evidence, theory, and in-depth qualitative research. 'ADvisor for Patients' is designed to support ways of understanding antidepressants and to help people to withdraw more successfully. It provides resources to build confidence for, and to support, stopping including side-effect management, addressing concerns, depression relapse prevention and stress management. The application of the person-based approach [22–24] has ensured our intervention is grounded a rich understanding of patients' psychosocial context.

Discontinuation can be complex [10], and the digital ADvisor intervention is designed to be an information-based resource to support patients, alongside monitoring and review from their General Practitioner (GP, Family Doctor). A separate digital intervention has been developed for GPs and other primary care professionals, called 'ADvisor: Health Professionals'. The patient intervention will also be used with additional brief telephone guidance (up to an hour, spread over three calls by trained psychological practitioners), to support use of the material. Guided digital/internet-based resources have been found to be consistently more effective than unguided digital interventions [37] for mental health problems. Guidance in this context is especially important as patients are withdrawing from pharmacotherapy, thus close monitoring is necessary.

For peer review only

Digital intervention for antidepressant discontinuation

The intervention will be implemented in a feasibility randomised controlled trial, where we will carry out a full qualitative [38] and quantitative [35] process study. We will explore how people engage with the intervention and how it affects their discontinuation experience. On this basis, as in the latter stages of the PBA [24], we will continue to modify the intervention ahead of a fully powered main trial.

There are some limitations to consider. Our recruitment for our qualitative work was from a limited, relatively affluent, geographical area in the south of England. The majority of our participants were women in both the primary qualitative work and the think-aloud interviews. While this does reflect the higher rates of antidepressant use for depression in women [39], it may be that our findings do not accurately reflect the views of men on long-term antidepressants. In the think-aloud interview sample, only nine of the 15 participants were taking antidepressants long-term for depression or low mood. The intervention contains information on preventing depression relapse and focuses on the symptoms of depression and anxiety which may not be applicable to these individuals. As such, some members of our sample may not have adequately represented the target population for this intervention, which may have introduced bias in our findings.

To conclude, psychologically informed interventions may improve the chances of effective discontinuation from antidepressants. ADvisor is a theory- evidence-, and

For peer review only

person-based digital intervention that may provide this support. The feasibility, clinical and cost-effectiveness of ADvisor now needs to be determined.

For peer review only

Digital intervention for antidepressant discontinuation

Funding Statement

This work was supported by NIHR Programme Grant for Applied Research (PGfAR)grant number RP-PG-1214-20004.

Data Sharing

This is a qualitative study and therefore the data is not suitable for sharing beyond what is contained within the report. Further information can be requested from the corresponding author.

Competing Interests

Dr. Kendrick reports grants from National Institute for Health Research, during the conduct of the study. Dr. Moncrieff reports grants from National Institute of Health Research, during the conduct of the study; and is a member of the Council for Evidence-based Psychiatry which is an unfunded organisation, whose mission is to 'communicate evidence of the potentially harmful effects of psychiatric drugs to the people and institutions in the UK that can make a difference'. All other authors have no competing interest to disclose.

Author contribution

TK led on the grant application for the six-year REDUCE programme. EM conducted two systematic reviews and SW conducted primary qualitative interviews which informed the intervention content. AG and HB conducted theoretical modelling,

For peer review only

behavioural analysis and developed guiding principles. HB drafted intervention content and discussed with the intervention development team (AG and MG) and the wider team (TK, SW, GL, CM, CD, JM, RL, YN and GA). MG developed the intervention into a digital format using Lifeguide software and led on intervention testing. Think aloud interviews were conducted by HB, SW and TK. RL provided support with recruitment for think aloud interviews. Think aloud transcripts were coded by HB and the results were discussed with AG, GL, TK and CM for interpretation. HB, MG and AG refined the intervention in line with patient feedback, with comments from the wider team when necessary. The manuscript was prepared by HB and AG, and has been approved by all co-authors.

Patient and Public Involvement

Patient and public members of the REDUCE team were involved in discussions about the design and recruitment for this study, and were invited to comment on initial drafts of the interview schedules. Patient and public colleagues viewed prototype intervention content and provided comment on these drafts. Patient and public members of the REDUCE team were included in group discussions about the feedback from think aloud interviews and any resulting amendments to the intervention content.

References

1 Health and Social Care Information Centre. Prescriptions Dispensed in the Community - Statistics for England, 2006-2016 - NHS Digital. 2017.https://digital.nhs.uk/data-and-information/publications/statistical/prescriptions-dispensed-in-the-community/prescriptions-dispensed-in-the-community-statistics-for-england-2006-2016-pas (accessed 2 Jul 2018).

2 *Health at a Glance: Europe 2012*. OECD 2012. doi:10.1787/9789264183896-en

3 Mojtabai R, Olfson M. National trends in long-term use of antidepressant medications: results from the U.S. National Health and Nutrition Examination Survey. *J Clin Psychiatry* 2014;**75**:169–77. doi:10.4088/JCP.13m08443

4 Scholes S, Faulding S, Mindell J. Use of prescribed medicines. In: *Health and Social Care Information Centre*. 2014. https://files.digital.nhs.uk/publicationimport/pub16xxx/pub16076/hse2013-ch5-pres-meds.pdf (accessed 25 Jun 2018).

5 Ambresin G, Palmer V, Densley K, *et al.* What factors influence long-term antidepressant use in primary care? Findings from the Australian diamond cohort study. *J Affect Disord* 2015;**176**:125–32. doi:10.1016/j.jad.2015.01.055

6 Cruickshank G, MacGillivray S, Bruce D, *et al.* Cross-sectional survey of patients in receipt of long-term repeat prescriptions for antidepressant drugs in

For peer review only

primary care. *Ment Health Fam Med* 2008;**5**:105–9.

7 Piek E, Kollen BJ, van der Meer K, *et al.* Maintenance Use of Antidepressants in Dutch General Practice: Non-Guideline Concordant. *PLoS One* 2014;**9**:e97463. doi:10.1371/journal.pone.0097463

8 Kendrick T, Stuart B, Newell C, *et al.* Did NICE guidelines and the Quality Outcomes Framework change GP antidepressant prescribing in England? Observational study with time trend analyses 2003–2013. *J Affect Disord* 2015;**186**:171–7. doi:10.1016/j.jad.2015.06.052

9 Ferguson JM. SSRI Antidepressant Medications: Adverse Effects and Tolerability. *Prim Care Companion J Clin Psychiatry* 2001;**3**:22–7. doi:10.4088/PCC.v03n0105

10 Maund E, Dewar-Haggart R, Williams S, *et al.* Barriers and facilitators to discontinuing antidepressant use: A systematic review and thematic synthesis. *J Affect Disord* 2019;**245**:38–62. doi:10.1016/j.jad.2018.10.107

11 Sinclair JE, Aucott LS, Lawton K, *et al.* The monitoring of longer term prescriptions of antidepressants: Observational study in a primary care setting. *Fam Pract* 2014;**31**:419–26. doi:10.1093/fampra/cmu019

12 Eveleigh R, Grutters J, Muskens E, *et al.* Cost-utility analysis of a treatment advice to discontinue inappropriate long-term antidepressant use in primary care. *Fam Pract* 2014;**31**:578–84. doi:10.1093/fampra/cmu043

13 Johnson CF, Macdonald HJ, Atkinson P, *et al.* Reviewing long-term antidepressants can reduce drug burden: A prospective observational cohort

For peer review only

study. *Br J Gen Pract* 2012;**62**:773–9. doi:10.3399/bjgp12X658304

14 Fava GA, Rafanelli C, Grandi S, *et al.* Prevention of Recurrent Depression With Cognitive Behavioral Therapy. *Arch Gen Psychiatry* 1998;**55**:816–20.

15 Fava GA, Gandi S, Zielezny M, *et al.* Cognitive behavioral treatment of residual symptoms in primary major depressive disorder. *Am J Psychiatry* 1994;**151**:1295–9. doi:10.1176/ajp.151.9.1295

16 Huijbers MJ, Spinhoven P, Spijker J, *et al.* Discontinuation of antidepressant medication after mindfulness-based cognitive therapy for recurrent depression: Randomised controlled non-inferiority trial. *Br J Psychiatry* 2016;**208**:366–73. doi:10.1192/bjp.bp.115.168971

17 Kuyken W, Byford S, Watkins E, *et al.* Mindfulness-Based Cognitive Therapy to Prevent Relapse in Recurrent Depression. *J Consult Clin Psychol* 2008;**76**:966–78. doi:10.1037/a0013786

18 Kuyken W, Hayes R, Barrett B, *et al.* Effectiveness and cost-effectiveness of mindfulness-based cognitive therapy compared with maintenance antidepressant treatment in the prevention of depressive relapse or recurrence (PREVENT): a randomised controlled trial. *Lancet* 2015;**386**:63–73. doi:10.1016/S0140-6736(14)62222-4

19 Office for National Statistics. Internet users, UK:2018 Office for National Statistics. 2018;:8.https://www.ons.gov.uk/businessindustryandtrade/itandinternetindustry/bulletins/internetusers/2018

For peer review only

20 Andersson G, Carlbring P, Titoy N, *et al.* Internet interventions for adults with anxiety and mood disorders: A narrative umbrella review of recent meta-analyses. *Can J Psychiatry*

21 Bartholomew LK, Parcel GS, Kok G. Intervention Mapping: A Process for Developing Theory and Evidence-Based Health Education Programs. *Heal Educ Behav* 1998;**25**:545–63. doi:10.1177/109019819802500502

22 Band R, Bradbury K, Morton K, *et al.* Intervention planning for a digital intervention for self-management of hypertension: a theory-, evidence-and person-based approach. doi:10.1186/s13012-017-0553-4

23 Yardley L, Morrison L, Bradbury K, *et al.* The Person-Based Approach to Intervention Development: Application to Digital Health-Related Behavior Change Interventions. *J Med Internet Res* 2015;**17**:e30. doi:10.2196/jmir.4055

24 Yardley L, Ainsworth B, Arden-Close E, *et al.* The person-based approach to enhancing the acceptability and feasibility of interventions. *Pilot Feasibility Stud* 2015;**1**:37. doi:10.1186/s40814-015-0033-z

25 Maund E, Stuart B, Moore M, *et al.* Managing Antidepressant Discontinuation: A Systematic Review. *Ann Fam Med* 2019;**17**:52–60. doi:10.1370/afm.2336

26 Braun V, Clarke V. Using thematic analysis in psychology. *Qual Res Psychol* 2006;**3**:77–101. doi:10.1191/1478088706qp063oa

27 Joffe H, Yardley L. Content and thematic analysis. In: *Research Methods for Clinical and Health Psychology*. London: : Sage 2004. 56–68.

28 Michie S, van Stralen MM, West R. The behaviour change wheel: A new

For peer review only

method for characterising and designing behaviour change interventions. Published Online First: 2011. doi:10.1186/1748-5908-6-42

29 Michie S, Atkins L, West R. *The behaviour change wheel: a guide to designing interventions*. London: : Silverback Publishing 2015.

30 Bandura A. Social cognitive theory of self-regulation. *Organ Behav Hum Decis Process* 1991;**50**:248–87. doi:10.1016/0749-5978(91)90022-L

31 Murray E, Treweek S, Pope C, *et al.* Normalisation process theory: a framework for developing, evaluating and implementing complex interventions. *BMC Med* 2010;**8**:63. doi:10.1186/1741-7015-8-63

32 Bandura A. Health promotion from the perspective of social cognitive theory. 1998;**13**.http://www.tandfonline.com/doi/pdf/10.1080/08870449808407422 (accessed 2 Oct 2017).

33 Horne R, Chapman SCE, Parham R, *et al.* Understanding Patients' Adherence-Related Beliefs about Medicines Prescribed for Long-Term Conditions: A Meta-Analytic Review of the Necessity-Concerns Framework. *PLoS One* 2013;**8**:e80633. doi:10.1371/journal.pone.0080633

34 Clifford S, Barber N, Horne R. Understanding different beliefs held by adherers, unintentional nonadherers, and intentional nonadherers: Application of the Necessity–Concerns Framework. *J Psychosom Res* 2008;**64**:41–6. doi:10.1016/j.jpsychores.2007.05.004

35 Moore G, Audrey S, Barker M, *et al.* Process evaluation of complex interventions UK Medical Research Council (MRC) guidance.

For peer review only

https://www.mrc.ac.uk/documents/pdf/mrc-phsrn-process-evaluation-guidance-final/ (accessed 3 Aug 2017).

36 Ryan RM, Deci EL. Self-determination theory and the facilitation of intrinsic motivation, social development, and well-being. *Am Psychol* 2000;**55**:68–78.http://www.ncbi.nlm.nih.gov/pubmed/11392867 (accessed 10 May 2019).

37 Baumeister H, Reichler L, Muzinger M, *et al.* The impact of guidance on Internet-based mental health interventions - A systematic review. *Internet Interv* 2014;**1**:205–15. doi:10.1016/j.invent.2014.08.003

38 O'Cathain A, Hoddinott P, Lewin S, *et al.* Maximising the impact of qualitative research in feasibility studies for randomised controlled trials: guidance for researchers. *Pilot Feasibility Stud* 2015;**1**:32. doi:10.1186/s40814-015-0026-y

39 Scholes S, Faulding S, Mindell J. Use of Prescribed Medicines. In: *Health Survey for England 2013: Health, Social Care and Lifestyles.* Leeds: : Health and Social Care Information Centre 2014. www.hscic.gov.uk/pubs/hse2013

For peer review only

Digital intervention for antidepressant discontinuation

ADvisor Guiding Principles	
Design objectives	*Key (distinctive) design features*
To **build confidence** that discontinuing antidepressant medication is safe and achievable over the long-term	• Offer an evidence-based rationale for how withdrawal and replacement with psychological./behavioural alternatives will help.<br>• Provide withdrawal success stories and examples (modelling).<br>• Address concerns patients may have re withdrawal (side effects, symptoms) from their previous experiences – demonstrate empathy and acknowledge real barriers to change.<br>• Offer motivational support.
To be an accessible, motivating resource that supports patients in managing their withdrawal in a	• Foster autonomy through choice and a non-prescriptive approach, providing explanations for all suggestions.<br>• Offer a broad range of strategies from

Table 1. Guiding Principles for the ADvisor intervention.

Digital intervention for antidepressant discontinuation

manner that aligns with **their preferences**	quick support in managing withdrawal symptoms, to more in-depth modules on a mindful approach to preventing depression relapse, and behavioural strategies for managing day-to-day stressors.<br>• Provide options for self-tailoring to personal experiences and barriers<br>• Provide a simple, attractive interface, with a focus on accessibly of content<br>•

peer review only

Digital intervention for antidepressant discontinuation

Table 2. Outline content of the digital intervention.

Content	Description
Reducing and stopping antidepressants	An introduction to the intervention, which addresses motivations behind withdrawal, asking participants to reflect on why they might prefer to discontinue antidepressant treatment. Guidance on when to speak to their GP/nurse and advice on following a tapering regime.
Thinking about antidepressants	Acknowledging that antidepressant treatment is not necessarily required long-term and that the mechanisms are more complex than correcting a serotonin deficiency.
I'm worried about stopping	Addressing participant fears by signposting participants to appropriate resources in ADvisor.
Dealing with withdrawal symptoms	Guidance for dealing with mild withdrawal symptoms (including guided practices for accepting/tolerating unpleasant symptoms). Advice for patients to contact their GP for assistance with moderate or severe withdrawal symptoms.
Keeping well	Relapse prevention techniques grounded in Mindfulness-Based Cognitive Therapy.
Thinking about what you value	Reflection on values and committed action to values (through goal setting), based on Acceptance and Commitment Therapy.
Moving forward	Psychoeducation and techniques for managing distress (e.g. mindfulness and behaviour activation) provided through a distress-management online intervention, Healthy Paths.
My Notes	Where patients can access content from other sections where they have written their own responses (for example their own reasons for wanting to stop antidepressants and their own warning signs and triggers for relapse).
Resources	Direct links to resources in ADvisor (e.g. activity planning and information for family and friends).

For peer review only

Digital intervention for antidepressant discontinuation

Characteristics	*N (%)*
Females	9 (60)
Males	6 (40)
Married	11 (73.3)
cohabiting	2 (13.3)
Single	2 (13.3)
Employed	9 (60)
Not currently in employment	6 (40)

For peer review only

Digital intervention for antidepressant discontinuation

Table 3. Think aloud qualitative study characteristics.

Diagnosis	
Depression/low mood	9 (60)
Fibromyalgia	2 (13.3)
Unknown	2 (13.3)
Urethritis	1 (6.7)
Post Traumatic Stress Disorder	1 (6.7)
Successfully stopped before	8 (53.%)
Currently taking antidepressants	14 (93.3%)
	**Mean (SD)**
Years on antidepressants	10.43 (7.27)
PHQ-9 score	4.53 (2.50)

Digital intervention for antidepressant discontinuation

Figure 1. Logic model ADvisor intervention alongside additional components

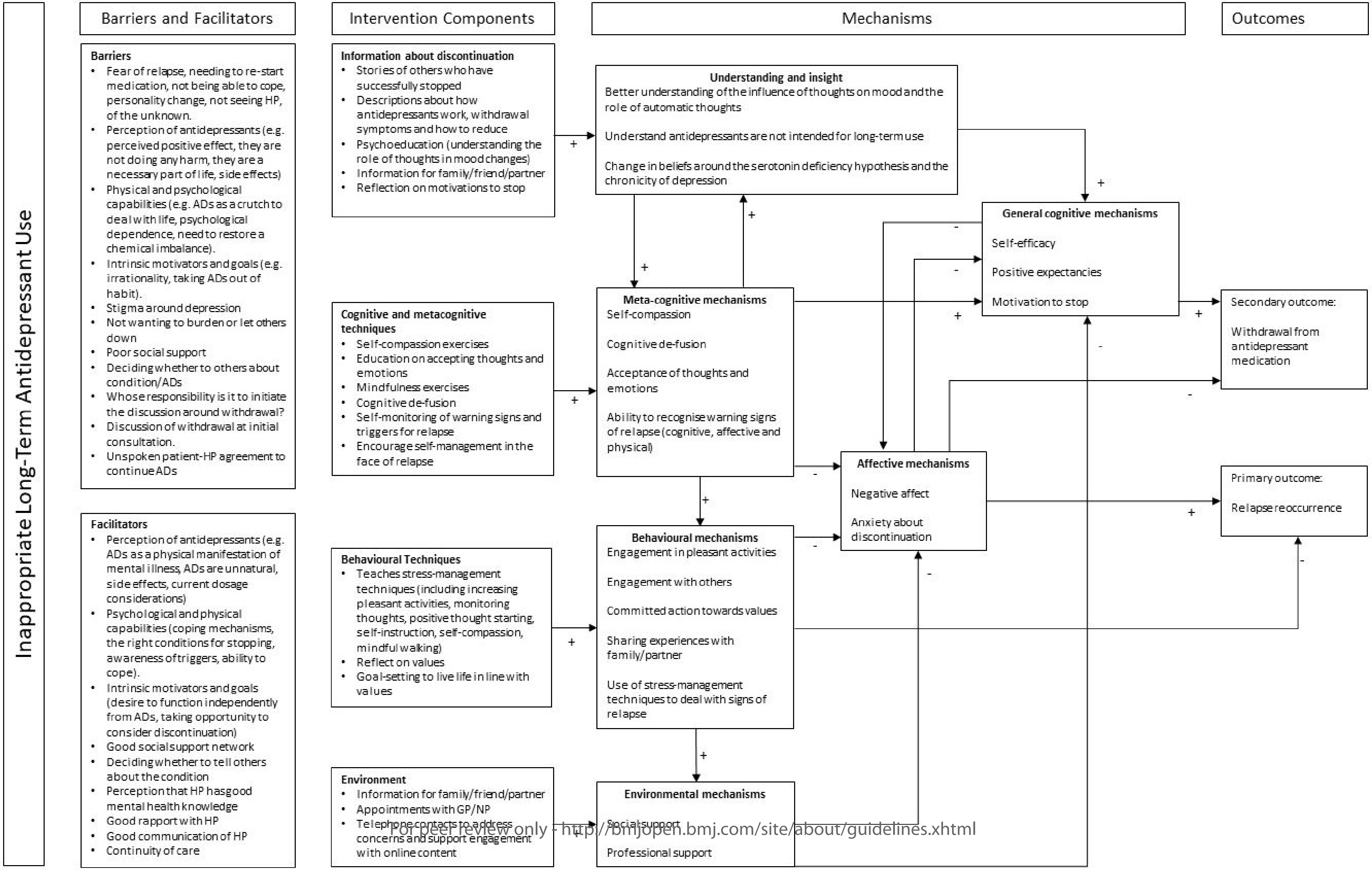

For peer review only - http://bmjopen.bmj.com/site/about/guidelines.xhtml

Digital intervention for antidepressant discontinuation

For peer review only

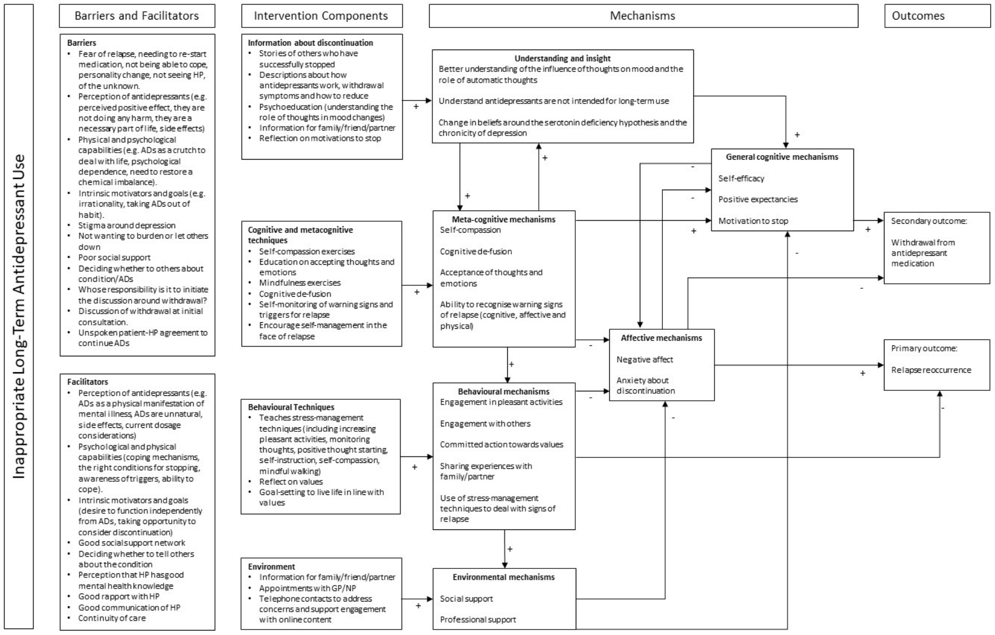

Figure 1. Logic model ADvisor intervention alongside additional components

355x266mm (96 x 96 DPI)

Appendix A – Behavioural Diagnosis

**Target behaviour: Reducing and stopping antidepressant medication**		
**BCW/COM-B Components**	**What needs to happen for the target behaviour to occur?**	**Proposed intervention element**
**Physical capability**<br>*Physical skill, strength or stamina*	• Understanding how to reduce doses physically: e.g. how to take tapered medication appropriately, in order to reduce the occurrence of side effects.	• **GP**<br>• **Internet intervention modules**<br>• **Telephone support**
**Psychological capability**<br>*Knowledge or psychological skills, strength or stamina to engage in necessary mental processes*	• Detailed, accessible guidance on the withdrawal process in general (setting up appropriate expectations)<br>• Improving knowledge on how to withdraw (practicalities)<br>• Developing psychological skills to manage the process:<br>○ Managing psychological side effects of withdrawal<br>○ Understanding helpful appraisals of symptoms<br>○ Learning about the prevention of relapse, managing fear of recurrence<br>○ Developing skills to manage life-stressors cognitively and behaviourally<br><br>*Social Cognitive Theory (SCT) and research will be broadly drawn on to ensure information/techniques are described and applied to align with evidence-based principles for increasing self-efficacy*	• **Internet intervention modules**<br>• (Telephone support)

For peer review only

**Physical opportunity**<br>*Opportunity afforded by the environment involving time recourses, locations, cues, physical affordance*	• Ability to access and get to GP appointments/pharmacy to collect reduced dose antidepressants	• **General practitioner (as a function of usual care)**<br>• **Telephone support/advice**
**Social opportunity**<br>*Opportunity afforded by interpersonal influences, social cues and cultural norms that influence the way we think about things*	• Close social network (family/friends) of patient may need to be supportive of the withdrawal process/attempt	• **Brief overview material developed for family members/friends**
**Reflective motivation**<br>*Reflective processes involving evaluations/beliefs about what is good and bad, and plans (self-conscious intentions)*	• Modification of beliefs about depression:<br>○ Exploring the nature of depression in a way that aligns with behavioural/cognitive management<br>○ Discussing impact of beliefs and expectations about chronicity<br>○ Exploring effect of analogies with physical conditions (diabetes/asthma)<br>○ Acknowledging complexity re our understanding of depression in an accessible manner<br>• Modification of beliefs about antidepressant medication:<br>○ Addressing beliefs about addiction/dependency<br>○ Exploring the serotonin hypothesis; evidence, balanced implications, rationale for behaviour/cognition to substitute medication	• **Internet intervention modules**<br>• **Internet intervention modules**

For peer review only

	• Foster motivation to withdraw through discussion of benefits, reduction of side effects, potential for increase in agency, potential for effective use of alternatives to pharmacological management<br><br>• Facilitate clear planning for the withdrawal process e.g. human contacts, management strategies, access to rapid/emergency support<br><br>*Inductive qualitative work (meta-synthesis and primary qualitative research) and theory will be used to inform this material*	• **General practitioner**<br><br>• **Telephone support/advice**
**Automatic motivation**<br>*Automatic processes involving emotional reactions, desires (wants and needs) impulses, inhibitions, drive states and reflex responses*	• Encourage awareness of automatic disruptive modes/thought process that may trigger or be triggered by symptoms<br><br>• Work on developing habitual healthier responses to symptom occurrences	• **Internet intervention modules**
**Behavourial diagnosis of the relevant COM-B components**	Although all areas of the COM-B model will need to be addressed to some extent, **psychological capability** and **reflective motivation** are likely to be the key targets for a supported digital intervention to help patients withdraw from antidepressant medication	

References:

1. Michie S, van Stralen MM, West R. The behaviour change wheel: a new method for characterising and designing behaviour change interventions. Implement Sci. 2011;6:42.
2. Michie SF, Atkins L, West R. The behaviour change wheel: a guide to designing interventions. London: Silverback Publishing; 2015

For peer review only

Appendix B – Theoretical Modelling

**Intervention module**	**Page**	**Content**	**Evidence:** Importance of barrier/facilitator content targets OR evidence for effectiveness of content	**BCW construct**	**BCW function**	**BCTs (Taxonomy V1)** Techniques broadly applied across content sections	**SCT construct** Constructs applied across content sections	**NPT construct** Constructs applied across content sections
**Reducing and stopping antidepressants**	Welcome							
	Why should I reduce and stop?	Foster a motivation to withdraw through discussion of benefits, reduction of side effects, potential for increase in agency, potential for effective use of alternatives to medication	*Bosman et al. (2016); Dickinson et al. (2010); Verbeek-Heida and Mathot (2006); Iden et al. (2011); Karp (1993); Knudsen et al. (2002); Eveleigh (2015); Gibson (2016); Schofield (2011).*	Reflexive motivation	Enablement; training; education	9.1 Credible source<br><br>9.2 Pros and cons<br><br>15.2. Persuasion about capability<br><br>13.2 Framing-reframing	Knowledge; social outcome expectations; physical outcome expectations; Self-efficacy (Somatic and emotional states)	Coherence: Individual specification<br><br>Cognitive participation: Initiation
	The downsides	Reflection on the side effects of antidepressants as a means to foster motivation to withdraw		Reflexive motivation	Enablement; training; education			

	When should I reduce and stop?	Highlighting that it is best to start withdrawal at a stable time in life		Psychological capability	Enablement; training; education			
	What to expect	Outline the discontinuation process: that the GP will provide a schedule, that this is flexible and that there may be side effects but there are ways to manage these and they are often short-lived.		Psychological capability	Enablement; training; education			
	Addressing concerns	Briefly acknowledges that many people have concerns about withdrawal but that there are techniques for dealing with this in AD-visor		Psychological capability	Enablement; training; education			
	How can my GP help?	Outline the role of the GP in discontinuation,	*Bosman et al. (2016); Dickenson et al.*	Physical capability	Enablement; training; education			

		when to go to the GP for support.	*2010; Grime & Pollock (2003); Verbeek-Heida and Mathot (2006); Eveleigh (2015); Gibson (2016); Leydon et al. (2007) ; Cartwright (2016)*					
	Planning ahead	Overview of the process: GP will give schedule and as one tapers, there is support in AD-visor that can be used		Reflexive motivation	Enablement; training; education			
	Support from family and friends	Highlight how friends and family members can play and important role	*Bosman et al. (2016); Cromartry (2011); Verbeek-Heida and Mathot (2006); Eveleigh (2015*)	Social opportunity	Enablement; training; education	3.1 Social support 3.3 Social support (emotional)		
**How to reduce antidepressants**	How to reduce	Practical information about tapering schedules		Physical capability	Enablement; training; education	4.1 Instructions on how to perform behaviour	Self-efficacy (Mastery experiences/vicarious	Coherence: Individual specification

	How to reduce (2)	Highlight that there is unlikely to be a need for liquid formulations or pill cutters but if needed, the GP can offer some guidance (perhaps via community pharmacist)		Physical capability	Environmental restructuring; Enablement; training; education	6.1 Demonstration of behaviour (modelling)	experiences).	
	When to reduce	Reiterate that there are ideal times to begin tapering, such as when no major life events are expected		Psychological capability	Enablement; training; education			
**Thinking about antidepressants**	What are antidepressants?	Briefly explains what antidepressants are used for. Highlights that while it was believed they work through increasing serotonin, we now know it is more complex than that.	*Bosman et al. (2016); Dickenson et al. 2010; Grime & Pollock (2003); Verbeek-Heida and Mathot (2006); Karp (1993); Knudsen et al. (2002); Eveleigh (2015); Gibson (2016); Cartwright*	Reflexive motivation	Enablement; training; education	13.2 Framing/reframing<br><br>15.2. Persuasion about capability	Social outcome expectations; Knowledge; physical outcome expectations	Coherence: Internalisation

	Can I stop taking them?	Key point: even though we don't know exactly *how* they work, we do know that many people can successfully discontinue	*(2016); Leydon et al. (2007).*	Reflexive motivation	Enablement; training; education			
	Other forms of 'antidepressant'	There are things other than medication which can improve mood. The relationship between brain and behaviour is highlighted through a study which shows that CBT can result in changes in the brain		Reflexive motivation	Enablement; training; education			
	How to antidepressants work	Highlights again that we don't know exactly how they work but we do know: ADs help some people and not others and many		Reflexive motivation	Enablement; training; education			

		people can successfully stop.						
**I'm worried about stopping**	I'm worried about stopping	Highlight that many people have concerns about stopping and this is understandable and does not mean you won't be able to discontinue	Bosman et al. (2016); Dickinson et al. (2010); Verbeek-Heida and Mathot (2006); Iden et al. (2011); Karp (1993); Knudsen et al. (2002); Eveleigh (2015); Gibson (2016); Schofield (2011); Leydon et al. (2007).	Psychological capability	Enablement; training; education	13.2 Framing/reframin g<br><br>15.2. Persuasion about capability	Knowledge, Self-efficacy (Mastery experiences vicarious experiences).<br><br>Social outcome expectations; Knowledge; physical outcome expectations	Cognitive participation: Initiation<br><br>Cognitive participation: Activation
	Successful stopping	Indicate that many people stop SD without problems, and those who are worried can overcome their concerns		Psychological capability	Enablement; training; education			
	Concerns about stopping	Patients will be given a selection of options to click on to read more about specific concerns		Psychological capability	Enablement; training; education			
	How will I cope if something big happens?	Reassure that AD-visor has guidance on managing stress in difficult		Psychological capability	Enablement; training; education			

For peer review only

		situations. Signpost to Moving Forward module.						
	What if I go back to how I was before?	Reassure that AD-visor has guidance on preventing relapse and signpost to Keeping Well module.		Psychological capability	Enablement; training; education			
	What if I have to start taking antidepressants again?	Reassure that hopefully this won't be necessary because they will learn how to prevent relapse, but if it is, they can try withdrawing again in future		Psychological capability	Enablement; training; education			
	How will I manage my responsibilities?	Guidance on planning activities and highlight the importance family support as well as the timing of the tapering process		Psychological capability	Enablement; training; education			

For peer review only

	Dealing with worries	Reflecting on the motivations to discontinue and weighing these up against concerns.		Reflexive motivation	Enablement; training; education			
**Keeping well**	Keeping well	Introduce to the idea of relapse prevention	Kuyken (2008); Allen (2009); Kuyken (2010); Fava (1998); Cromarty (2011); Otto (2010);	Psychological capability	Enablement; training; education	11.2 Reduce negative emotions<br><br>13.2 Framing/reframin g<br><br>6.1 Demonstration of behaviour<br><br>4.3 Reattribution	Knowledge, Goals Self-efficacy (Mastery experiences vicarious experiences).<br><br>Social outcome expectations; Knowledge; physical outcome expectation	Cognitive participation: Activation
	Automatic pilot	Define running on autopilot and explain negative automatic thoughts		Psychological capability	Enablement; training; education			
	The power of thoughts	Explain how the way we think impacts mood and teach cognitive defusion (thoughts are not facts)		Psychological capability	Enablement; training; education			
	Let it be	Defining the term 'acceptance' and why it is useful in dealing		Psychological capability	Enablement; training; education			

		with difficult thoughts and feelings						
	Recognising warning signs	Explaining and reflecting on what thoughts and physical sensations might be indicators of relapse		Psychological capability	Enablement; training; education			
	Recognising triggers	Reflecting on situations that might trigger a relapse		Psychological capability	Enablement; training; education			
	Recognising relapse	Writing down warning signs and triggers and saving these to view later		Psychological capability	Enablement; training; education			
	Responding differently	Highlight that you cannot change thoughts or the things that happen in life, but you have a choice how to respond to these. Responding in more helpful		Psychological capability	Enablement; training; education			

		ways can prevent relapse.						
	Preventing relapse	1. Take a breath<br>2. Make a decision on how to act<br>3. Take action		Psychological capability	Enablement; training; education			
**Living life with values and goals***	What are values	Defines values as like a compass point providing direction for our lives.	Swain et al. 2013; Powers et al. 2009.	Psychological capability	Enablement; training; education	11.2 Reduce negative emotions<br><br>13.2 Framing/reframing<br><br>6.1 Demonstration of behaviour	Knowledge, Goals	Coherence: Internalisation
	What do I value?	Provides a space to write down what they value		Psychological capability	Enablement; training; education			
	Goals	Explaining the need to set goals in order to		Psychological capability	Enablement; training; education			

		act in line with our values				4.3 Reattribution		
	Setting goals	Guidance and space to write goals		Psychological capability	Enablement; training; education			
	Meeting goals	Reminds users to revisit this section to review their goals and see if they have met them		Psychological capability	Enablement; training; education			
**Dealing with withdrawal symptoms**	What are withdrawal symptoms?	Describes what they are and that they are a consequence of the brain and body adapting to the change in medication	*Bosman et al. (2016); Dickinson et al. (2010); Verbeek-Heida and Mathot (2006); Iden et al. (2011); Karp (1993); Knudsen et al. (2002); Eveleigh (2015); Gibson (2016); Schofield (2011); Leydon et al. (2007)*	Psychological capability Physical capability	Enablement; training; education	13.2 Framing/reframin g<br><br>6.1 Demonstration of behaviour<br><br>4.3 Reattribution	Social outcome expectations; Knowledge; physical outcome expectations	Cognitive participation: Activation
	Recognisin g withdrawal symptoms	This page highlights that there are different symptoms that might be physical or mental. Specific details of what symptoms may occur are not given.		Psychological capability Physical capability	Enablement; training; education			

	Thinking about withdrawal symptoms	Explains that the way we think about symptoms can change how much impact they have (e.g. if you mistake a withdrawal symptom for relapse, it may be harder for the symptom to pass).		Psychological capability Physical capability	Enablement; training; education			
	Knowing the difference	Details about the differences between withdrawal symptoms and relapse.		Psychological capability Physical capability	Enablement; training; education			
	Dealing with withdrawal symptoms	Mild symptoms can be tolerated and will pass, moderate symptoms can be treated by a doctor, and severe symptoms may indicate a slower taper is needed.		Psychological capability Physical capability	Enablement; training; education			

	Accepting withdrawal symptoms	Guidance on accepting/tolerating symptoms based on acceptance and commitment exercises used with chronic physical symptoms		Psychological capability Physical capability	Enablement; training; education			
**Moving forward**	Healthy Paths Through Stress intervention (Healthy Paths). See Geraghty et al. 2017 for full description	This module is based on an intervention aimed at managing life stresses. The modules have been developed as part of a separate project and their content will be incorporated into AD-visor. This section will include guidance on mindfulness practices and behavioural activation.	Muñoz et al. 2005; Geraghty et al. 2016.	Psychological capability	Enablement; training; education	11.2 Reduce negative emotions<br><br>13.2 Framing/reframing<br><br>6.1 Demonstration of behaviour<br><br>4.3 Reattribution	Knowledge, Goals Self-efficacy (Mastery experiences vicarious experiences).<br><br>Social outcome expectations; Knowledge; physical outcome expectations	Coherence: Individual specification<br><br>Coherence: Internalisation<br><br>Cognitive participation: Initiation<br><br>Cognitive participation: Activation

For peer review only - http://bmjopen.bmj.com/site/about/guidelines.xhtml

New references added:

Bosman, R. C., Huijbregts, K. M., Verhaak, P. F. M., Ruhé, H. G., Van Marwijk, H. W. J., Van Balkom, A. J. L. M., & Batelaan, N. M. (2016). Long-term antidepressant use: A qualitative study on perspectives of patients and GPs in primary care. *British Journal of General Practice*, *66*(651), e708–e719. https://doi.org/10.3399/bjgp16X686641

Cartwright, C., Gibson, K., & Read, J. (2016). Long-term antidepressant use : patient perspectives of benefits and adverse effects, 1401–1407. https://doi.org/10.2147/PPA.S110632

Cromarty, P., Jonsson, J., Moorhead, S., & Freeston, M. H. (2011). Cognitive Behaviour Therapy for Withdrawal from Antidepressant Medication: A Single Case Series. *Behavioural and Cognitive Psychotherapy*, *39*(01), 77–97. https://doi.org/10.1017/S1352465810000512

Dickinson, R., Knapp, P., House, A. O., Dimri, V., Zermansky, A., Petty, D., … Raynor, D. K. (2010). Long-term prescribing of antidepressants in the older population: A qualitative study. *British Journal of General Practice*, *60*(573), 257–259. https://doi.org/10.3399/bjgp10X483913

Eveleigh, R. H. (2015). *Inappropriate long-term antidepressant use in primary care: A challenge to change*. Radboud University Nijmegen. https://doi.org/10.1063/1.3033202

Fava, G. A., Rafanelli, C., Grandi, S., Conti, S., & Belluardo, P. (1998). Prevention of Recurrent Depression With Cognitive Behavioral Therapy. *Arch Gen Psychiatry*, *55*, 816–820.

Geraghty, A. W. A., Muñoz, R. F., Yardley, L., Mc Sharry, J., Little, P., & Moore, M. (2016). Developing an Unguided Internet-Delivered Intervention for Emotional Distress in Primary Care Patients: Applying Common Factor and Person-Based Approaches. *JMIR Ment Health*, *3*(4), e53.

Gibson, K., Cartwright, C., & Read, J. (2016). "In my life antidepressants have been..": A qualitative analysis of users' diverse experiences with antidepressants. *BMC Psychiatry*, *16*(1), 1–7. https://doi.org/10.1186/s12888-016-0844-3

Grime, K. P. J. (2002). Understanding Depression and its Treatment: GP, Patient and Pharmacist Perspectives, (September), 52–60. Retrieved from http://www.pharmacyresearchuk.org/waterway/wp-content/uploads/2012/11/Understanding_depression_and_its_treatment1.pdf

Iden, K. R., Hjørleifsson, S., & Ruths, S. (2011). Treatment decisions on antidepressants in nursing homes: A qualitative study. *Scandinavian Journal of Primary Health Care*, *29*(4), 252–256. https://doi.org/10.3109/02813432.2011.628240

Karp, D. A. (1993). Taking anti-depressant medications: Resistance, trial commitment, conversion, disenchantment. *Qualitative Sociology*, *16*(4), 337–359. https://doi.org/10.1007/BF00989969

Knudsen, P., Hansen, E. H., Traulsen, J. M., & Eskildsen, K. (2002). Changes in Self-Concept While Using SSRI Antidepressants. *Qualitative Health Research*, *12*(7), 932–944. https://doi.org/10.1177/104973202129120368

For peer review only

Kuyken, W., Byford, S., Watkins, E., Holden, E., White, K., King 's, B. B., … Teasdale, J. D. (2008). Mindfulness-Based Cognitive Therapy to Prevent Relapse in Recurrent Depression. *Journal of Consulting and Clinical Psychology*, *76*(6), 966–978. https://doi.org/10.1037/a0013786

Kuyken, W., Watkins, E., Holden, E., White, K., Taylor, R. S., Byford, S., … Dalgleish, T. (2010). How does mindfulness-based cognitive therapy work? *Behaviour Research and Therapy*, *48*, 1105–1112. https://doi.org/10.1016/j.brat.2010.08.003

Leydon, G. M., Rodgers, L., & Kendrick, T. (2007). A qualitative study of patient views on discontinuing long-term selective serotonin reuptake inhibitors. *Family Practice*, *24*(6), 570–575. https://doi.org/10.1093/fampra/cmm069

Muñoz, R. F., & Mendelson, T. (2005). Toward evidence-based interventions for diverse populations: The San Francisco General Hospital prevention and treatment manuals. *J Consult Clin Psychol*, *73*(5), 790-799.

Otto, M. W., McHugh, R. K., Simon, N. M., Farach, F. J., Worthington, J. J., & Pollack, M. H. (2010). Efficacy of CBT for benzodiazepine discontinuation in patients with panic disorder: Further evaluation. *Behaviour Research and Therapy*, *48*(8), 720–727. https://doi.org/10.1016/J.BRAT.2010.04.002

Powers, M. B., Zum Vorde Sive Vording, M. B., & Emmelkamp, P. M. (2009). Acceptance and commitment therapy: a meta-analytic review. *Psychother Psychosom*, *78*(2), 73-80.

Schofield, P., Crosland, A., Waheed, W., Aseem, S., Gask, L., Wallace, A., … Tylee, A. (2011). Patients' views of antidepressants: From first experiences to becoming expert. *British Journal of General Practice*, *61*(585), 142–148. https://doi.org/10.3399/bjgp11X567045

Swain, J., Hancock, K., Hainsworth, C., & Bowman, J. (2013). Acceptance and commitment therapy in the treatment of anxiety: a systematic review. *Clin Psychol Rev*, *33*(8), 965-978.

Verbeek-Heida, P. M., & Mathot, E. F. (2006). Better safe than sorry — why patients prefer to stop using selective serotonin reuptake inhibitor (SSRI) antidepressants but are afraid to do so: results of a qualitative study. *Chronic Illness*, *2*(2), 133–142. https://doi.org/10.1179/174592006X111003

For peer review only

For peer review only

Appendix C – Interview Schedule

REDUCE

UNIVERSITY OF Southampton
School of Medicine

REDUCE Study Workstream (WS) 3: REviewing long-term anti-Depressant treatment Use by Careful monitoring in Everyday practice

THINK-ALOUD INTERVIEW SCHEDULE WITH PATIENTS

Below is a list of topics/questions to be discussed in this study. The qualitative work will remain flexible with respect to participants' agendas but we will cover the broad topics/questions noted. It is common in qualitative work to iteratively develop topics and questions as new ideas emerge from early data collection. Therefore, we may add new topics as the interviews progress and data collection continues. However, the key topics of exploring participants' views of the prototype intervention will remain the same.

Introduction

1. Re-introduce self and purpose of interview

2. Check with participant:

- That they are still willing to be interviewed, and to be audio recorded
- Remind them it will take approximately 60 to 90 minutes
- That they are comfortable in a quiet place where they will not be disturbed

3. Remind participant that:

- Their responses will be kept confidential, and quotes used in the results will not identify them as an individual;
- They can change their mind about taking part in the study and stop the interview at any point.

4. Remind the participant that you will start by asking them some questions about their experiences with antidepressants. Remind the participant that you want them to look at the website and use it as they normally would, but say everything that they are thinking out loud. Tell them that you will remind them to do this so that they don't forget as it is

For peer review only - http://bmjopen.bmj.com/site/about/guidelines.xhtml

very easy to forget and that there are no right or wrong answers as it is their views that are important to us.

5. Ask if the participant has any questions.

6. Start recording.

For peer review only

Section 1: Demographic Data

We would like to collect some personal information to help us describe the range of people / experiences we have collected, so could you please let me know your

Age	
Gender	M / F
Do you live alone or with someone (friends / partner / family)?	
Single / in a relationship / married?	
Employed / retired / full time carer / stay at home parent?	
Job title	
Currently on ADs?	Y / N
Successfully stopped ADs before?<br>NB. ‘Success’ = been off ADs & experienced symptom free episode(s).	Y / N
Same GP for review or different GPs within practice?	
Current Medical Diagnosis for ADs (if known)	

For peer review only - http://bmjopen.bmj.com/site/about/guidelines.xhtml

Do you pay for your prescriptions?	
Have you ever taken any sick leave from work due to depression / anxiety / stress? If yes, how much?	
Have you ever needed a carer/ or to be cared for due to depression? If yes, by whom?	
Any other medical conditions?	
Have you ever taken St John's Wort?	
Any other relevant information?	
Participant ID	
Date screened by researcher / confirm eligible	
Urban or rural location? (*researcher observation*)	
Deprivation level of area? (*researcher observation*)	

Section 2: Background history of use of antidepressants.

1. Can you tell me a little bit about when you were first prescribed antidepressants?

Prompt: Feelings about how decision to go on antidepressants was made/managed. Experience of taking ADs.

2. Could you describe your experience of taking antidepressants for me now?

Prompt: Any intent to stop? Have you found antidepressants have helped to improve your condition? Side effects/benefits? Expectations of ADs vs. lived experience.

3. Can you tell me about your current depression treatment?

Prompt:

- Regular repeat prescriptions?
- Any self-help or counselling / therapy?
- How often are you reviewed by a GP, nurse or counsellor/therapist? Feelings around frequency?
- Continuity of care?
- What treatment would you say has helped you most / least?

For peer review only - http://bmjopen.bmj.com/site/about/guidelines.xhtml

Section 3: Previous attempts to discontinue / successful withdrawal. Barriers and enablers to discontinuation (including individual / social factors).

1. Can you tell me about a time when you stopped or thought about stopping your antidepressants?

Prompt: What were your reasons for wanting to stop? How long did you stop for? What was it that made you stay on your antidepressants? Withdrawal experiences / effects. How would you feel if you had to restart your antidepressants or increase the dose (if stopped/stopping)? Explore expectations around withdrawal.

Section 4: Think-aloud and researcher prompts

Explain to them that you want them to look at the website and use it as they normally would, but say everything that they are thinking out loud. Tell them that you will remind them to do this so that they don't forget as it is very easy to forget. If you think it would help then get them to try counting the windows in their house whilst saying everything that they are thinking out loud.

- [only on first page] What are your first impressions of this page?
- What are you thinking now?
- What made you choose that option?
- What do you think about [this activity, this information]?
- Can you tell me a bit more about that?
- What is it you like about that?
- That's really interesting……

Section 5: Post-think-aloud questions

- Overall, what do you think about this website?
- Can you tell me about anything that you liked about the website?
- Was there anything that you found surprising in the website?
- Can you tell me anything about the website that you were less keen on?
- Can you tell me about anything that you think should be changed?
- What would you think if your GP or practice nurse asked you to use the website?

For peer review only - http://bmjopen.bmj.com/site/about/guidelines.xhtml

- If you were withdrawing from your antidepressants, which parts of AD-visor do you think you would like to look at and why? (E.g. dealing with withdrawal symptoms, information about how antidepressants work, relapse prevention, mindfulness etc.).
- When people use this website for real, they will be offered some support over the telephone. If you were using the programme for real, what would you think of this option to get support over the phone?
- What are your thoughts about telephone support throughout the trial in general? [Researcher to explain trial design].
- If you did have opportunity to have support over the telephone, which of the topics in AD-visor do you think would be most useful to discuss over the phone?

ANY OTHER TOPICS YOU WOULD LIKE TO DISCUSS?

ANY QUESTIONS?

Debrief

- Tell participant that the digital recorder is now being switched off.
- Thank participant for taking part in the interview.
- Revisit consent
- Ask if the participant has any questions about the study.
- Let them know that you will be sending all participants a summary of study findings.
- Check happy for data to be used for teaching / secondary analysis.
- Thank participant again for taking part in the interview.

For peer review only - http://bmjopen.bmj.com/site/about/guidelines.xhtml

BMJ Open

Supporting antidepressant discontinuation: The development and optimisation of a digital intervention for patients in UK primary care using a theory-, evidence-, and person-based approach

Journal:	*BMJ Open*
Manuscript ID	bmjopen-2019-032312.R1
Article Type:	Original research
Date Submitted by the Author:	08-Nov-2019
Complete List of Authors:	Bowers, Hannah; University of Southampton, Primary Care, Population Sciences and Medical Education<br>Kendrick, Tony; University of Southampton, Primary Care, Population Sciences and Medical Education<br>Glowacka, Marta; Bournemouth University, Faculty of Health and Social Sciences<br>Williams, Samantha; University of Southampton, Primary Care, Population Sciences and Medical Education<br>Leydon, Geraldine; University of Southampton, Primary Care, Population Sciences and Medical Education<br>May, Carl; London School of Hygiene and Tropical Medicine Faculty of Epidemiology and Population Health<br>Dowrick, CF; University of Liverpool,<br>Moncrieff, Joanna; University College London and North East London mental health trust, Mental Health Sciences<br>Laine, Rebecca; University of Southampton, Primary Care, Population Sciences and Medical Education<br>Nestoriuc, Yvonne; Helmet Schmidt University, Department of Psychology<br>Andersson, Gerhard; Linkoping University; Karolinska Institutet, Department of Clinical Neuroscience and Psychiatry<br>Geraghty, Adam; University of Southampton, Primary Care, Population Sciences and Medical Education
**Primary Subject Heading**:	Mental health
Secondary Subject Heading:	Mental health, General practice / Family practice
Keywords:	Depression & mood disorders < PSYCHIATRY, Intervention Development, Digital Intervention, Antidepressants, PRIMARY CARE

SCHOLARONE™
Manuscripts

I, the Submitting Author has the right to grant and does grant on behalf of all authors of the Work (as defined in the below author licence), an exclusive licence and/or a non-exclusive licence for contributions from authors who are: i) UK Crown employees; ii) where BMJ has agreed a CC-BY licence shall apply, and/or iii) in accordance with the terms applicable for US Federal Government officers or employees acting as part of their official duties; on a worldwide, perpetual, irrevocable, royalty-free basis to BMJ Publishing Group Ltd ("BMJ") its licensees and where the relevant Journal is co-owned by BMJ to the co-owners of the Journal, to publish the Work in this journal and any other BMJ products and to exploit all rights, as set out in our licence.

The Submitting Author accepts and understands that any supply made under these terms is made by BMJ to the Submitting Author unless you are acting as an employee on behalf of your employer or a postgraduate student of an affiliated institution which is paying any applicable article publishing charge ("APC") for Open Access articles. Where the Submitting Author wishes to make the Work available on an Open Access basis (and intends to pay the relevant APC), the terms of reuse of such Open Access shall be governed by a Creative Commons licence – details of these licences and which Creative Commons licence will apply to this Work are set out in our licence referred to above.

Other than as permitted in any relevant BMJ Author's Self Archiving Policies, I confirm this Work has not been accepted for publication elsewhere, is not being considered for publication elsewhere and does not duplicate material already published. I confirm all authors consent to publication of this Work and authorise the granting of this licence.

For peer review only

Supporting antidepressant discontinuation: The development and optimisation of a digital intervention for patients in UK primary care using a theory-, evidence-, and person-based approach

Hannah M Bowers*[1], Tony Kendrick[1], Marta Glowacka[2], Samantha Williams[1], Geraldine M Leydon[1], Carl May[3], C F Dowrick[4], Joanna Moncrieff[5], Rebecca Laine[1], Yvonne Nestoriuc[6], Gerhard Andersson[7,8], Adam W A Geraghty[1].

[1] Primary Care, Population Sciences and Medical Education, University of Southampton, UK.

[2] Faculty of Health and Social Sciences, Bournemouth University, UK.

[3] Department of Health Services Research and Policy, London School of Hygiene and Tropical Medicine, UK

[4] Institute of Population Health Sciences, University of Liverpool, UK.

[5] Division of Psychiatry, University College London, UK.

[6] Department of Psychology, Helmut Schmidt University, Germany.

[7] Department of Behavioural Sciences and Learning, Linkoping University, Sweden.

[8] Department of Clinical Neuroscience and Psychiatry, Karolinska Institutet, Sweden.

…

For peer review only

Digital intervention for antidepressant discontinuation

*Corresponding Author: H.M.Bowers@soton.ac.uk. Primary Care and Population Sciences, University of Southampton, Aldermoor Health Centre, Aldermoor Close, Southampton, SO15 5HT. Tel: 02380 591743

…

Abstract

Objectives: We aimed to develop a digital intervention to support antidepressant discontinuation in UK primary care that is scalable, accessible, safe and feasible. In this paper we describe the development using a theory- evidence- and person-based approach.

Design: Intervention development using a theory-, evidence-, and person-based approach

Setting: Primary Care in the South of England

Participants: Fifteen participants with a range of antidepressant experience took part in 'think aloud' interviews for intervention optimisation

Intervention: Our digital intervention prototype (called 'ADvisor') was developed on the basis of a planning phase consisting of qualitative and quantitative reviews, an in-depth qualitative study, the development of guiding principles and a theory-based behavioural analysis. Our optimisation phase consisted of 'think aloud' interviews where the intervention was iteratively refined.

Results: The qualitative systematic review and in-depth qualitative study highlighted the centrality of fear of depression relapse as a key barrier to discontinuation. The quantitative systematic review showed that psychologically informed approaches such as cognitive

For peer review only

behaviour therapy (CBT) were associated with greater rates of discontinuation than simple advice to reduce. Following a behavioural diagnosis based on the Behaviour Change Wheel, Social Cognitive Theory provided a theoretical basis for the intervention. The intervention was optimised on the basis of think aloud interviews, where participants suggested they like the flexibility of the system and found it reassuring. Changes were made to the tone of the material and the structure was adjusted based on this qualitative feedback.

Conclusions: 'ADvisor' is an evidence-, theory- and person-based digital intervention designed to support antidepressant discontinuation. The intervention was perceived as helpful and reassuring in optimisation interviews. Trials are now needed to determine the feasibility, clinical and cost effectiveness of this approach.

278 word (BMJOpen limit 300).

For peer review only

Strengths and Limitations of the study

- A systematic review and qualitative meta-synthesis were conducted alongside primary qualitative work to guide the content of the intervention.
- A theory-based behavioural analysis and the development of guiding principles further informed the planning phase of intervention development.
- Think aloud interviews provided in-depth understanding of patients' views of the intervention in terms of usability and content.
- The intervention was iteratively refined throughout the think aloud interviews to produce an intervention that aligns with patient preference.
- Think aloud participants were predominantly White British and from more affluent regions in the South of England and may not represent the views of all antidepressant users.

For peer review only

Digital intervention for antidepressant discontinuation

Introduction

The number of antidepressant prescriptions in the UK has continued to rise over the past four decades [1], a trend which has also been seen in the United States and across Europe [2,3]. Approximately 10% of adults in the UK are currently prescribed antidepressant medication [4]. Though antidepressants can prevent relapse, there is evidence that 30-50% of patients on long-term antidepressants have no indication based on guidelines for long-term use [5–7]. Research suggests this increase in prescribing is primarily due to general practitioners (GPs) prescribing antidepressants for longer and longer durations over time [8]. Long-term antidepressant use is both costly to the UK National Health Service (NHS) (in terms of prescription and appointment costs) and is associated with increased side effects [9]. Attempting to discontinue antidepressants in the 30-50% with no indication for long-term use may therefore be beneficial to patients and positively impact on use of health-care resources.

There are many factors that may contribute to long-term antidepressant use, including the occurrence of a physiological withdrawal syndrome following reduction or cessation and psychological factors such as beliefs about the necessity of long-term use and fear of relapse [10]. Infrequent reviews of patients taking antidepressants may also contribute to sustained use [11]. However, simply prompting for patient reviews has resulted in discontinuation rates of 6-8%, not

For peer review only

Digital intervention for antidepressant discontinuation

significantly differing from usual care [12,13]. This highlights the potential importance of psychologically informed interventions to support withdrawal.

Trials have shown that Cognitive Behavioural Therapy (CBT) and Mindfulness-Based Cognitive Therapy (MBCT) can effectively support discontinuation of antidepressants, with cessation rates ranging from between 55%-95% [14–18]. Although producing positive outcomes, these interventions involve intensive group/face-to-face courses, thus access and ability to scale up within resource-strapped health services may be severely limited. There is a need for accessible, scalable psychologically-informed interventions that can effectively support individuals where discontinuation is appropriate.

In the UK, 89% of the general population in 2018 used the internet weekly, up from 55% in 2006 [19]. Internet-based digital interventions supported with human contact have been shown to effectively reduce depression and anxiety [20]. Digital intervention may have potential to provide a scalable, accessible way of supporting appropriate antidepressant discontinuation. We aimed to develop such a supported digital intervention as part of the UK-based REDUCE (REviewing long term antiDepressant Use by Careful monitoring in Everyday practice) programme to develop and trial safe, feasible and effective ways to support patients withdrawing from antidepressants where appropriate.

For peer review only

In this paper we describe the planning and optimisation of our patient-facing digital intervention to support discontinuation, named 'ADvisor'. This paper provides an overview of the different stages of development and how these together informed a digital intervention. Some of this work has implications beyond intervention development and further details are therefore published elsewhere. This paper is instead focused on the particular work involved in developing a digital intervention.

Methods

Phase 1: Intervention planning and development

There is a range of systematic protocols for intervention development that can be drawn on at the outset of a development project (e.g. Intervention Mapping [21]). We chose to implement a theory-, evidence- and person-based approach [22]. This comprehensive strategy integrates the person-based approach (PBA) [23,24] with more commonly used theory and evidenced-based methods. The PBA provides guidance for integrating systematic in-depth qualitative research into the development process. Drawing on the PBA ensures evidence and theory-based techniques are applied with a full understanding of the target users' perspectives and psychosocial context [23]. We will outline the components of our comprehensive

For peer review only

approach including systematic reviewing, primary qualitative research, development of guiding principles, behavioural analysis and logic modelling.

Systematic reviewing

Two systematic reviews were conducted: a quantitative review with meta-analysis, and a qualitative thematic synthesis, described in detail elsewhere [10,25]. The qualitative review searched nine databases from inception to February 2017 and updated searches were carried out in July 2018. Citation searching, reference list checking and related article checking was also performed. The quantitative review involved searching eight databases from inception to March 2017. Citations and reference lists were searched for full papers that met the inclusion criteria. Both searches were developed by an experience librarian and systematic reviewer. Further details of the search strategies can be found in the full publications of these reviews [10,25].

For intervention planning, from the quantitative review we drew out interventions that had successfully supported discontinuation and considered their intervention components, seeking full manuals where possible. We aimed to determine which components could be best translated into a digital format. In the qualitative review we identified barriers and facilitators to antidepressant discontinuation. Barriers and facilitators were tabulated and used to inform the 'Guiding Principles' (see below) as well as content for the intervention.

For peer review only

Primary qualitative research

Individual semi-structured interviews were conducted by SW with primary care patients with varying experiences of antidepressants, and varying levels of motivation to stop, with the aim to explore experiences of antidepressant discontinuation. These interviews explored patients' views on barriers and facilitators to withdrawal, the role of health care professionals in supporting withdrawal attempts, and elements of a proposed intervention to support withdrawal. Interviews were conducted at the patients' homes or their GP practices and were audio recorded and transcribed verbatim. Analysis was conducted following thematic analytic principles suggested by Braun and Clarke [26], and Joffe and Yardley [27]. Analysis was conducted by SW (a qualitative researcher). The coding manual and developed themes were discussed and agreed by the wider development group. Only the findings related to the development of the intervention are described in this paper. Further details of the methods and the findings related to the broader aims of this piece of qualitative work will be published elsewhere.

Development of guiding principles

Guiding principles are a fundamental part of the PBA [23]. They represent broad design objectives that guide the application/implementation of the core intervention strategies, aiming to increase engagement [24]. Guiding principles were developed based on the qualitative synthesis [10] and primary qualitative findings. Through this

For peer review only

qualitative work we aimed to identify key behavioural needs, challenges or issues the intervention needed to address.

Behavioural analysis

Behavioural and implementation theory was drawn on as we triangulated between the qualitative and quantitative evidence, and the expert views of our team (including patient representatives, GPs, psychiatrists, psychologists, sociologists and health services researchers) to determine important intervention components. Using the Behaviour Change Wheel and COM-B model of behavior (Capability, Opportunity, Motivation – Behaviour) [28], informed by our qualitative research, we conducted a 'behavioural diagnosis' [29]. In behavioural diagnosis, factors that are likely to affect the central target behaviour are considered in terms of capability, opportunity, and motivation [28,29]. Once we had proposed initial intervention content/components, these were mapped theoretically using the Behaviour Change Wheel, Social Cognitive Theory (SCT) [30] and Normalisation Process Theory [31]. As well as providing a mapped full description of the proposed intervention, this process ensured we did not miss areas of theory that may have improved the intervention.

Phase 2: Intervention optimisation

Design

For peer review only

Within the PBA, 'think aloud' qualitative studies are employed to optimise the prototype intervention. Think aloud studies are designed to elicit in-depth perspectives about the nature of the content, rather than solely focusing on functionality and usability. Ethical approval for the study was granted by NHS South Central Oxford B Research Ethics Committee.

Participants

Participants were recruited from eight primary care practices in the South of England. Eligibility criteria were as follows: Inclusion criteria: Taking antidepressants for more than one year for a first episode or two years for a subsequent episode; discontinued antidepressants, or were in the process of tapering. Exclusion criteria: PHQ-9 scores greater than or equal to 10 (suggesting persisting symptoms of depression) and those who reported any suicide ideation; history of suicide attempts; ongoing social difficulties or recent life events likely to provoke relapse; more than three previous significant episodes of depression; comorbid psychosis, bipolar disorder, obsessive-compulsive disorder, or substance use (or past history of these conditions); or currently receiving psychiatric treatment.

Procedure

Eligible participants met with a researcher (HB, SW or TK) either in their own home or at their primary care practice to take part in a think-aloud interview. Interviews invited participants to engage with the prototype intervention using a study laptop

For peer review only

and say what they were thinking, aloud in real time. The interviewer prompted participants when necessary (for example asking patients 'How do you feel about the information on this page?'). Interviews ranged from 38 to 93 minutes in length and were audio recorded, and transcribed verbatim. The interview ended when patients concluded they had looked at all the information they would like to see or if the interview length was approaching 90 minutes. The amount of intervention content the patient saw therefore depended on their own preferences and the time they took to look at the information. The interview schedule can be found in Appendix A. There were three primary iterations of interviews based on three key modified prototype interventions. Patients at the start of the study therefore saw different versions of the intervention to those who were recruited later rounds. This allowed the changes made as a result of patient feedback to continue to be tested. Interviews with patients continued until data saturation was reached, defined here as when comments about the intervention reflected that no further changes were necessary according to the person-based approach and when there were no new codes identified as part of the thematic analysis.

Analysis

Transcribed interviews were analysed using two primary analytic methods. The first analytic method was a more rapid coding than thematic analysis, which involves using coding tables designed for the PBA, where positive and negative comments were tabulated. Core problematic issues likely to affect participant engagement or

For peer review only

intervention effectiveness identified using this coding method were brought to the broader group, and amendments to the intervention agreed. Alongside this method, a more in-depth thematic analysis [26,27] was developed to capture patient views of the intervention and ideas about how they might engage with it, beyond comments on what might be amended. For this latter analysis, HB independently coded the transcripts and discussed a preliminary coding frame with a second researcher (AG). Theme labelling and interpretation were discussed and agreed by the team. The thematic analysis is presented here. Therefore while the initial analysis informed what changes were necessary, the thematic analysis explored what patients thought about the intervention in greater depth. These analyses were related in that some things that were identified in our initial analysis informed the development of themes.

Results

Phase 1: Intervention planning and development

Systematic reviewing

Our qualitative thematic synthesis (see [10] for full results) across 22 studies highlighted key barriers and facilitators to discontinuation. Patients' concerns regarding their ability to cope and psychological dependence were common barriers,

For peer review only

as were difficulties experienced in previous stopping attempts. Confidence in abilities to stop, effective coping strategies and stable life circumstances facilitated discontinuation. Additional important themes included fear of relapse – this was the central fear that prohibited stopping attempts – and beliefs about depression. The belief that depression was a long-term condition caused by biochemical changes in the brain was a key barrier to discontinuation. Where patients reported a very different belief, that depression was due to changing life circumstances, this seemed to facilitate discontinuation. Patients' self-identity and goals were an important factor: Having self-identifying as “old” or “disabled” acted as a barrier to discontinuation, and having goals to function independently functioned as facilitator to discontinuation.

In the quantitative systematic review (see [25] for full results) a variety of therapeutic techniques were implemented including a patient-specific letter to the GP with a recommendation to discontinue plus tapering advice; GP review of the patient's condition and medication; CBT plus tapering; MBCT with tapering support gradual discontinuation and one-week tapering. The results indicated that CBT or MBCT plus tapering are helpful for patients discontinuing antidepressants, with cessation rates of 40-95% [23], compared to only 6-8% cessation where health professionals are simply prompted to review patients. CBT plus tapering resulted in lower relapse rates compared with clinical management plus taper (15-25% vs 35-80%) [23]. The content of the interventions were extracted and feasibility of delivery in a digital

For peer review only

format was considered. We developed a module based closely on MBCT protocols on the basis of this review.

The findings from both reviews' findings informed the guiding principles, behavioural analysis and logic model, which formed the basis for intervention content selection and development.

Primary qualitative research

Five themes were developed through the thematic analysis of 19 patient interviews (full details will be published elsewhere). A summary is presented here. Participants spoke of the centrality of personal medication and health care factors, for example some patients described the need for a personalised tapering regime to support them discontinuing. Beliefs about depression and its treatment were key in shaping participants' stance towards discontinuing. For example, ideas around the necessity of anti-depressant medication due to 'chemical imbalance' were common. Holding these beliefs made patients less likely to consider stopping. Fear of stopping, driven by fear of relapse were discussed as central barriers to withdrawal. The impact of others also appeared to be important. For example, the perception of stigma and the feeling of letting people down, made participants less willing to discontinue, while having a good support network was considered beneficial to stopping. Participants were also asked to consider digital methods of intervention delivery. Elements participants wanted to see in the intervention included explanation around how

For peer review only

antidepressants work, support for anxiety/fear of discontinuing, coping strategies and information on withdrawal symptoms. There was some concern around privacy and around preference for greater face-to-face interaction to support them during the discontinuation phase. Patients expressed a need to have accessible, interactive and information presented in an aesthetically pleasing way.

The full findings in our primary qualitative research mirrored and expanded the findings of our qualitative thematic synthesis. They fed into the guiding principles, behavioural analysis logic model and content for the intervention.

Guiding principles

On the basis of the qualitative work guiding principles were developed (comprised of design objectives and design features), see Table 1. We developed two broad design objectives: The first, regarding building confidence that discontinuing antidepressant medication is safe and achievable, was developed from prominent themes around fear of stopping, the need for confidence, and beliefs that antidepressant medications are needed long-term. The second objective, that the intervention should be an accessible, motivating resource that supports patients in managing their withdrawal in a manner that aligns with their preferences, was developed in response to the range of views and beliefs held about the nature of depression and why antidepressants were necessary. Design features that support both these objectives are listed in Table 1.

For peer review only

Digital intervention for antidepressant discontinuation

[Insert table 1 about here]

Behavioural analysis

Our behavioural diagnosis following the COM-B model can be found in Appendix B. Our target behaviour was reducing and stopping the taking of antidepressant medication. Based on our reviews, qualitative work and discussion amongst our broader team, psychological capability and reflective motivation were considered key constructs for changing the target behaviour. The results of our behavioural diagnosis are presented in Appendix B.

Following the drafting of module content and structure, we mapped content against 1) studies suggesting content would be important, 2) Behaviour Change Wheel (BCW) constructs, 3) Social Cognitive Theory (SCT), and 4) Normalisation Process Theory (NPT). See Appendix C for detailed theoretical mapping for our intervention content.

Fundamentally, SCT [32] underlies the approach taken in the intervention to facilitate behaviour change. The intervention is designed to increase self-efficacy for stopping and to modify outcome expectations e.g. increase positive expectation that the recommended strategies are likely to support effective discontinuation. At a later stage in development, the Necessity Concerns Framework (NCF) [33] was

For peer review only

considered. NCF was developed to explain the role of treatment beliefs on adherence behaviours. According to NCF, adherence to treatment is a function of patients' beliefs about the necessity of their medication and the concerns they have about it; high necessity beliefs and low concerns are likely to predict medication adherence [34]. In the context of antidepressant withdrawal, accordingly, we would need to reduce patients' beliefs about the necessity of the medication, highlight likely benefits of stopping, and reduce concern regarding the stopping process. All of these factors will ultimately impact on self-efficacy, hence the centrality of SCT in our theoretical modelling.

Logic modelling

Logic models represent proposed or hypothesised 'theories of change' outlining the problem/issue and barriers, ingredients mechanism, and how these may affect target outcomes [35]. We developed a draft logic model for the REDUCE patient intervention, drawing on theory, evidence and our person-based qualitative work, see Figure 1.

[Insert Figure 1 about here]

Outline intervention content

On the basis of our planning process, a prototype digital intervention was developed for patients taking antidepressants long-term (defined as more than one year for a

For peer review only

Digital intervention for antidepressant discontinuation

first episode or more than two years following two or more episodes). The contents of the online intervention are described in Table 2. A digital intervention for health professionals (providing information and guidance on antidepressant reduction) was also developed as part of the REDUCE programme and is reported separately.

[Insert Table 2 about here]

Content was developed using findings from the reviews of the literature, primary qualitative work, behavioural analysis and logic modelling. In addition to online content, scheduled telephone support contacts with specialists trained in providing psychological support and email reminders were developed as part of the patient intervention.

When accessing the ADvisor intervention for the first time, users view a core module with the central rationale for stopping antidepressants; they can then access a menu with a range of further modules based on our planning work. Aligning with our guiding principles, users are advised that they can use ADvisor how and when they would like. It is their tool, to be used to support them in a way that is consistent with their needs, preferences and experience. Through this approach we aimed to maximise autonomous motivation [36].

For peer review only

Digital intervention for antidepressant discontinuation

Content for the online intervention was initially drafted by a member of the content development team (HB) before AG and MG and then wider team members offered their expertise and informed further development of the content. This iterative process continued until all team members were satisfied that the prototype intervention addressed key experiences, barriers and facilitators identified by the work from phase one and were in line with the guiding principles, theoretical modelling and logic model. The content was transferred into online pages in LifeGuide (www.lifeguideonline.org) and further amendments to the presentation were made by the team before moving forward to the optimisation phase.

Phase 2: Intervention optimisation

Of the 42 patients who returned a postal reply slip expressing interest, 11 were ineligible, nine could not subsequently be contacted, two later declined, and five expressed an interest only after data saturation had been reached. This resulted in a final sample of 15 patients (see Table 3 for sample characteristics).

[Insert Table 3 about here]

Iterations of Advisor

There were three rounds of iterations of the intervention during the think-aloud interviews. Patients in round one were shown the first prototype. Changes made to

For peer review only

the version in round two included making the tone less formal, revising the introduction navigation and the wording to be more gentle. The 'my notes' section was also reorganized to be clearer and buttons to exit the intervention at the end of each module were removed to try to keep the patients on site for longer. In the version shown in round three some changes included further revision of the tone, some of the information was presented in a more aesthetically pleasing way and some links within the intervention to other modules were removed as these were confusing for patients.

Findings

Six themes were developed, namely: flexible use; familiarity with content; reassurance; utility of information; teaching of useful skills; and feeling supported. Patient identifiers and demographic information are presented below each quote, where round number refers to the iteration of the intervention that the patient saw.

Flexible use

Participants discussed how ADvisor could be used in different ways to suit the individual. When viewing the main menu page in ADvisor participants talked about how different sections would be more useful for them, and that some sections were not relevant for them at that particular time.

For peer review only

Dealing with withdrawal symptoms, I don't have any, so it's fine. That [keeping well and moving forward modules] I'm more interested in about because I think that's - for me, keeping well and moving forward is where I am and where I want to be.

[14/03/0001] [round 1]

Initial versions of the intervention included an introduction module within which participants could choose which of two options they would like to view first, though they would need to view both sections before moving onto the main menu. Some participants felt that this was in contradiction to the aim of choice and flexibility. We therefore modified the intervention so that the introduction was shorter and these two choices were moved to optional buttons in the main menu.

It's kind of saying you've really got to look at that one; otherwise, you will have flicked back through or I would have thought it might have been, if it's really flexible, user friendly, you might be allowed to skip that page because you could always revisit it again.

[01/01/0026] [round 1]

Participants not only varied in the topics they wanted to look at, but also in terms of the different exercises they would choose to engage with in ADvisor. Some

For peer review only

participants liked the idea of writing down their responses in ADvisor while others did not.

> *No. That's me. No, I'm very stoic and – just – I don't need to write it down, it's fine; I know what I'm doing, I'm fine, very much, I think.*
>
> *[01/01/0005] [round 2]*

> *I'd like to say that I would [write things down]; I think I probably would if I was – you know – really serious about it, because I like to write things down and if I haven't written it down, it can just go out of my brain. So I think, for me, it would be important to write that down.*
>
> *[05/01/0022] [round 2]*

Participants also discussed how ADvisor could be used in different ways. For example, it can be something used regularly, something one can pick up as and when necessary or it can be read through all in one go.

> *So it looks like you can use it when you want to but if you feel you're coping without, so it's not something you have to do all the time.*
>
> *[05/01/0022] [round 2]*

For peer review only

> *Yes, I would use it for future reference, as well, because you can always go backwards, can't you? With anything, I mean. If I ever came to a time where I was feeling down, I think, to go back on to something is to remind you. Because it's easy to forget.*
>
> *[13/01/0058] [round 3]*

Familiarity with content

Many of the participants referred to previous experience with psychological therapies or tools they have used in the past for their symptoms of depression. When reading cognitive-behavioural, acceptance and commitment, or mindfulness-based information in ADvisor, participants expressed a sense of familiarity with the terminology or messages they were presented.

> *Clicking on Breathing Space; that's very much mindfulness, isn't it? Yes, I like that, that's nice.*
>
> *[14/03/0001] [round 1]*

Some of the information about depression and antidepressants seemed to be obvious to a small number of participants who had pre-existing knowledge, but they understood that not all patients would have the same prior knowledge. One participant in particular who worked in healthcare found that much of the information was not new to her.

For peer review only

I'm obviously interested in reducing still further or coming off the antidepressants. ... See I don't think I can – I do know an awful lot about it and read a lot about it and very – sorry – but, you know, being in the business myself, it's all a bit Noddy to Big Ears.

[13/01/0033] [round 3] [works in healthcare]

Reassurance

Participants described a sense of fear around stopping antidepressants. This has been reported in previous qualitative studies of patient and health professional perspectives on stopping [10]. Participants in this study often reported feeling reassured by information in ADvisor. While participants differed in terms of which particular piece of information they found reassuring, some participants noted feeling reassured knowing that they could go back on their antidepressant if they felt necessary. Other participants found that knowing that withdrawal symptoms are often short-lived offered reassurance.

Well that's a good section because that is quite a worry, I think, for anybody wanting to come off them; it would worry me what would my side-effects be and how would I feel coming off them. So to actually – I mean I didn't know this – to actually say that they are often short lived and go away in a few days or weeks is quite encouraging, isn't it.

For peer review only

Digital intervention for antidepressant discontinuation

[04/01/0025] [round 3]

As fear of withdrawal symptoms was highlighted in the qualitative work, withdrawal symptoms were discussed at several points during the introduction module. However participants who were not initially concerned about withdrawal symptoms felt that this was setting an expectation for difficulty withdrawing. Whilst not minimising withdrawal-related problems, we therefore revised the language around concerns about withdrawal in the introduction.

> *Well it's very obvious withdrawal is a problem, looking at all the advice you can see to help you get over it, which – yes. There's a negative feeling there, if it's stressed to this degree on this program, then you're obviously expecting trouble.*

[10/03/0003]

Credibility of the information appeared to be important for participants. Participants liked to see the evidence base that was provided in ADvisor and in particular liked that it would be used within an NHS setting. The NHS affiliation seemed to provide a sense of reliability and credibility.

> *I'd be really pleased if they [GP/nurse] referred me to a website, especially if it was from the GP, because I think, well, it's backed up or supported by them.*

For peer review only

[14/03/0001] [round 1]

There was a balance that needed to be struck between portraying information as credible and maintaining a warm and friendly tone. Participants reported some of the information in ADvisor as sounding academic and reading like it could be used by practitioners. As a result, the tone was revised to be warmer and friendlier, while maintaining a sense of credibility.

> *It's just very business-like so very much like maybe something that a university would produce or maybe that a medical professional would share amongst themselves and your everyday person who's maybe not used to reading things in so much detail any more, sadly. It's quite dry.*

[14/03/0001] [round 1]

Utility of information

Participants described the information on withdrawal symptoms to be useful, in particular, some participants liked the information on how to distinguish between signs of relapse and withdrawal symptoms. One participant in particular expressed a shift in her views on discontinuing as a result of the information in ADvisor. She explained that had she known that withdrawal symptoms may feel like relapse and will pass, she may have persisted with her lower dose of antidepressant for longer.

For peer review only

Digital intervention for antidepressant discontinuation

She also highlighted that difficulty in getting a GP appointment is a barrier for her to persist with discontinuing in the face of difficulties.

> *.. I didn't know ... withdrawal symptoms might appear the same as the symptoms that led to needing antidepressants in the first place, but they will pass after a short time; I didn't know that. I thought if you started feeling down again, then you were heading for a crash.*
>
> *[13/03/0001] [round 2]*

Some participants described wanting more detailed information about what withdrawal symptoms might be expected. However, upon discussion with the broader study team, it was decided to avoid setting expectations around particular symptoms as this may lead patients to experience expected symptoms. Patients can instead request this information from their GP if it is something they feel they would rather know about. While this information is provided to GPs as part of our health professional intervention package, it must be acknowledged that there are limitations around access to GP appointments which may act as a barrier to getting information about withdrawal symptoms.

Participants also noted that it was useful to reflect on the side effects of taking antidepressants. There was an awareness that these can be hard to recognise, and three participants reported that after reading the information in ADvisor, they may in

For peer review only

fact have been experiencing side effects of which they were previously unaware. One participant described how this made him even more inclined to discontinue.

> *Well, as I look at these, I think maybe I'm wrong; maybe I am still getting side-effects, but I've just learned to accept them or – I'm just a little bit in denial and it makes me want to get off them even more, because then – lots of these things will, you know, will disappear.*
>
> *[12/03/0003] [round 1]*

Teaching of useful skills

Participants reported the skills included in ADvisor as being useful. In particular, advice around preventing relapse and mindfulness-based skills were considered to be useful.

> *Your triggers, recognising your emotions and reminding yourself that you don't have to react in a certain way; you can react in a different way. Yes, I think it's very good.*
>
> *[13/01/0001] [round 2]*

For peer review only

Acceptance of difficulties and of emotions was discussed as a useful coping strategy by participants, both with regards to their own pre-existing relationship to their emotions, and with regards to the messages in ADvisor on acceptance.

When you read it like that, it is true; the more you worry about things, the more down you get. So you've got to learn to stop doing that. I have to start putting that into practice if I'm going to do this.

[13/01/0058] [round 3]

Participants liked having tools and techniques in ADvisor for dealing with difficult emotions and life stresses. There was an understanding that life stress is often unavoidable, and participants expressed a desire to learn ways of dealing with stresses. Some participants stated that learning how to manage emotions would act as a replacement for taking antidepressants.

I think that exercise of sitting by the stream is very good, because I know when I had Cognitive Behavioural Therapy I was taught to – you know – when your thoughts came – to – and I still do this now – is always remember – say to yourself that it will pass, those feelings will pass and it might be horrible while you're going through those feelings, but find somewhere nice and comfortable to sit, with a blanket even, and that sort of thing.

[04/01/0025] [round 3]

For peer review only

By the final interviews in the final round, participants' comments were positive with no new issues being identified. This signified the intervention was now ready for further evaluation and feedback in the planned feasibility trial to follow.

Discussion

We developed a digital intervention to support appropriate antidepressant discontinuation. The intervention was developed through a process of triangulation between quantitative and qualitative review evidence, theory, and in-depth qualitative research. 'ADvisor for Patients' is designed to support ways of understanding antidepressants and to help people to withdraw more successfully. It provides resources to build confidence for, and to support, stopping including side-effect management, addressing concerns, depression relapse prevention and stress management. The application of the person-based approach [22–24] has ensured our intervention is grounded a rich understanding of patients' psychosocial context.

Discontinuation can be complex [10], and the digital ADvisor intervention is designed to be an information-based resource to support patients, alongside monitoring and review from their General Practitioner (GP, Family Doctor). A separate digital intervention has been developed for GPs and other primary care professionals, called 'ADvisor: Health Professionals'. The patient intervention will also be used with

For peer review only

additional brief telephone guidance (up to an hour, spread over three calls by trained psychological practitioners), to support use of the material. Guided digital/internet-based resources have been found to be consistently more effective than unguided digital interventions [37] for mental health problems. Guidance in this context is especially important as patients are withdrawing from pharmacotherapy, thus close monitoring is necessary.

The intervention will be implemented in a feasibility randomised controlled trial, where we will carry out a full qualitative [38] and quantitative [35] process study. We will explore how people engage with the intervention and how it affects their discontinuation experience. On this basis, as in the latter stages of the PBA [24], we will continue to modify the intervention ahead of a fully powered main trial.

There are some limitations to consider. Our recruitment for our qualitative work was from a limited, relatively affluent, geographical area in the south of England. The majority of our participants were women in both the primary qualitative work and the think-aloud interviews. While this does reflect the higher rates of antidepressant use for depression in women [39], it may be that our findings do not accurately reflect the views of men on long-term antidepressants. In the think-aloud interview sample, only nine of the 15 participants were taking antidepressants long-term for depression or low mood. The intervention contains information on preventing depression relapse and focuses on the symptoms of depression and anxiety which may not be

For peer review only

applicable to these individuals. As such, some members of our sample may not have adequately represented the target population for this intervention, which may have introduced bias in our findings. The average age of participants in our think-aloud interview sample was 55.2 years, which may be a reflection of the typical populations in the geographical locations in this study. In the feasibility trial and main trial phases of intervention testing, further qualitative work will be carried out with a larger and demographically wider population of patients from a range of different areas in the UK.

The researchers conducting the think-aloud interviews were involved in the development of the intervention. This may have resulted in bias when asking questions about the intervention. However in think-aloud interviews the patients often express their views in response to what they see on the page as opposed to solely responding to questions from the researcher. While prompting and follow-up questions might have been affected by researcher bias, patients were not aware the interviewers had designed and written elements of the intervention and were encouraged to provide both positive and negative feedback to the researchers.

To conclude, psychologically informed interventions may improve the chances of effective discontinuation from antidepressants. ADvisor is a theory- evidence-, and person-based digital intervention that may provide this support. The feasibility, clinical and cost-effectiveness of ADvisor now needs to be determined.

For peer review only

Digital intervention for antidepressant discontinuation

For peer review only

Funding Statement

This work was supported by NIHR Programme Grant for Applied Research (PGfAR)grant number RP-PG-1214-20004.

Data Sharing

This is a qualitative study and therefore the data is not suitable for sharing beyond what is contained within the report. Further information can be requested from the corresponding author.

Competing Interests

Dr. Kendrick reports grants from National Institute for Health Research, during the conduct of the study. Dr. Moncrieff reports grants from National Institute of Health Research, during the conduct of the study; and is a member of the Council for Evidence-based Psychiatry which is an unfunded organisation, whose mission is to 'communicate evidence of the potentially harmful effects of psychiatric drugs to the people and institutions in the UK that can make a difference'. All other authors have no competing interest to disclose.

Author contribution

TK led on the grant application for the six-year REDUCE programme. SW conducted primary qualitative interviews which informed the intervention content. AG and HB conducted theoretical modelling, behavioural analysis and developed guiding

For peer review only

principles. HB drafted intervention content and discussed with the intervention development team (AG and MG) and the wider team (TK, SW, GL, CM, CD, JM, RL, YN and GA). MG developed the intervention into a digital format using Lifeguide software and led on intervention testing. Think aloud interviews were conducted by HB, SW and TK. RL provided support with recruitment for think aloud interviews. Think aloud transcripts were coded by HB and the results were discussed with AG, GL, TK and CM for interpretation. HB, MG and AG refined the intervention in line with patient feedback, with comments from the wider team when necessary. The manuscript was prepared by HB and AG, and has been approved by all co-authors.

Patient and Public Involvement

Patient and public members of the REDUCE team were involved in discussions about the design and recruitment for this study, and were invited to comment on initial drafts of the interview schedules. Patient and public colleagues viewed prototype intervention content and provided comment on these drafts. Patient and public members of the REDUCE team were included in group discussions about the feedback from think aloud interviews and any resulting amendments to the intervention content.

Acknowledgments

The authors would like to acknowledge the work of Emma Maund while working on the REDUCE Programme, who conducted two systematic reviews which informed the intervention development.

For peer review only

References

1 Health and Social Care Information Centre. Prescriptions Dispensed in the Community - Statistics for England, 2006-2016 - NHS Digital. 2017.https://digital.nhs.uk/data-and-information/publications/statistical/prescriptions-dispensed-in-the-community/prescriptions-dispensed-in-the-community-statistics-for-england-2006-2016-pas (accessed 2 Jul 2018).

2 *Health at a Glance: Europe 2012*. OECD 2012. doi:10.1787/9789264183896-en

3 Mojtabai R, Olfson M. National trends in long-term use of antidepressant medications: results from the U.S. National Health and Nutrition Examination Survey. *J Clin Psychiatry* 2014;**75**:169–77. doi:10.4088/JCP.13m08443

4 Scholes S, Faulding S, Mindell J. Use of prescribed medicines. In: *Health and Social Care Information Centre*. 2014. https://files.digital.nhs.uk/publicationimport/pub16xxx/pub16076/hse2013-ch5-pres-meds.pdf (accessed 25 Jun 2018).

5 Ambresin G, Palmer V, Densley K, *et al.* What factors influence long-term antidepressant use in primary care? Findings from the Australian diamond cohort study. *J Affect Disord* 2015;**176**:125–32. doi:10.1016/j.jad.2015.01.055

6 Cruickshank G, MacGillivray S, Bruce D, *et al.* Cross-sectional survey of patients in receipt of long-term repeat prescriptions for antidepressant drugs in

For peer review only

Digital intervention for antidepressant discontinuation

primary care. *Ment Health Fam Med* 2008;**5**:105–9.

7 Piek E, Kollen BJ, van der Meer K, *et al.* Maintenance Use of Antidepressants in Dutch General Practice: Non-Guideline Concordant. *PLoS One* 2014;**9**:e97463. doi:10.1371/journal.pone.0097463

8 Kendrick T, Stuart B, Newell C, *et al.* Did NICE guidelines and the Quality Outcomes Framework change GP antidepressant prescribing in England? Observational study with time trend analyses 2003–2013. *J Affect Disord* 2015;**186**:171–7. doi:10.1016/j.jad.2015.06.052

9 Ferguson JM. SSRI Antidepressant Medications: Adverse Effects and Tolerability. *Prim Care Companion J Clin Psychiatry* 2001;**3**:22–7. doi:10.4088/PCC.v03n0105

10 Maund E, Dewar-Haggart R, Williams S, *et al.* Barriers and facilitators to discontinuing antidepressant use: A systematic review and thematic synthesis. *J Affect Disord* 2019;**245**:38–62. doi:10.1016/j.jad.2018.10.107

11 Sinclair JE, Aucott LS, Lawton K, *et al.* The monitoring of longer term prescriptions of antidepressants: Observational study in a primary care setting. *Fam Pract* 2014;**31**:419–26. doi:10.1093/fampra/cmu019

12 Eveleigh R, Grutters J, Muskens E, *et al.* Cost-utility analysis of a treatment advice to discontinue inappropriate long-term antidepressant use in primary care. *Fam Pract* 2014;**31**:578–84. doi:10.1093/fampra/cmu043

13 Johnson CF, Macdonald HJ, Atkinson P, *et al.* Reviewing long-term antidepressants can reduce drug burden: A prospective observational cohort

For peer review only

study. *Br J Gen Pract* 2012;**62**:773–9. doi:10.3399/bjgp12X658304

14 Fava GA, Rafanelli C, Grandi S, *et al.* Prevention of Recurrent Depression With Cognitive Behavioral Therapy. *Arch Gen Psychiatry* 1998;**55**:816–20.

15 Fava GA, Gandi S, Zielezny M, *et al.* Cognitive behavioral treatment of residual symptoms in primary major depressive disorder. *Am J Psychiatry* 1994;**151**:1295–9. doi:10.1176/ajp.151.9.1295

16 Huijbers MJ, Spinhoven P, Spijker J, *et al.* Discontinuation of antidepressant medication after mindfulness-based cognitive therapy for recurrent depression: Randomised controlled non-inferiority trial. *Br J Psychiatry* 2016;**208**:366–73. doi:10.1192/bjp.bp.115.168971

17 Kuyken W, Byford S, Watkins E, *et al.* Mindfulness-Based Cognitive Therapy to Prevent Relapse in Recurrent Depression. *J Consult Clin Psychol* 2008;**76**:966–78. doi:10.1037/a0013786

18 Kuyken W, Hayes R, Barrett B, *et al.* Effectiveness and cost-effectiveness of mindfulness-based cognitive therapy compared with maintenance antidepressant treatment in the prevention of depressive relapse or recurrence (PREVENT): a randomised controlled trial. *Lancet* 2015;**386**:63–73. doi:10.1016/S0140-6736(14)62222-4

19 Office for National Statistics. Internet users, UK:2018 Office for National Statistics. 2018;:8.https://www.ons.gov.uk/businessindustryandtrade/itandinternetindustry/bulletins/internetusers/2018

For peer review only

20 Andersson G, Carlbring P, Titoy N, *et al.* Internet interventions for adults with anxiety and mood disorders: A narrative umbrella review of recent meta-analyses. *Can J Psychiatry*

21 Bartholomew LK, Parcel GS, Kok G. Intervention Mapping: A Process for Developing Theory and Evidence-Based Health Education Programs. *Heal Educ Behav* 1998;**25**:545–63. doi:10.1177/109019819802500502

22 Band R, Bradbury K, Morton K, *et al.* Intervention planning for a digital intervention for self-management of hypertension: a theory-, evidence-and person-based approach. doi:10.1186/s13012-017-0553-4

23 Yardley L, Morrison L, Bradbury K, *et al.* The Person-Based Approach to Intervention Development: Application to Digital Health-Related Behavior Change Interventions. *J Med Internet Res* 2015;**17**:e30. doi:10.2196/jmir.4055

24 Yardley L, Ainsworth B, Arden-Close E, *et al.* The person-based approach to enhancing the acceptability and feasibility of interventions. *Pilot Feasibility Stud* 2015;**1**:37. doi:10.1186/s40814-015-0033-z

25 Maund E, Stuart B, Moore M, *et al.* Managing Antidepressant Discontinuation: A Systematic Review. *Ann Fam Med* 2019;**17**:52–60. doi:10.1370/afm.2336

26 Braun V, Clarke V. Using thematic analysis in psychology. *Qual Res Psychol* 2006;**3**:77–101. doi:10.1191/1478088706qp063oa

27 Joffe H, Yardley L. Content and thematic analysis. In: *Research Methods for Clinical and Health Psychology*. London: : Sage 2004. 56–68.

28 Michie S, van Stralen MM, West R. The behaviour change wheel: A new

For peer review only

method for characterising and designing behaviour change interventions. Published Online First: 2011. doi:10.1186/1748-5908-6-42

29 Michie S, Atkins L, West R. *The behaviour change wheel: a guide to designing interventions*. London: : Silverback Publishing 2015.

30 Bandura A. Social cognitive theory of self-regulation. *Organ Behav Hum Decis Process* 1991;**50**:248–87. doi:10.1016/0749-5978(91)90022-L

31 Murray E, Treweek S, Pope C, *et al.* Normalisation process theory: a framework for developing, evaluating and implementing complex interventions. *BMC Med* 2010;**8**:63. doi:10.1186/1741-7015-8-63

32 Bandura A. Health promotion from the perspective of social cognitive theory. 1998;**13**.http://www.tandfonline.com/doi/pdf/10.1080/08870449808407422 (accessed 2 Oct 2017).

33 Horne R, Chapman SCE, Parham R, *et al.* Understanding Patients' Adherence-Related Beliefs about Medicines Prescribed for Long-Term Conditions: A Meta-Analytic Review of the Necessity-Concerns Framework. *PLoS One* 2013;**8**:e80633. doi:10.1371/journal.pone.0080633

34 Clifford S, Barber N, Horne R. Understanding different beliefs held by adherers, unintentional nonadherers, and intentional nonadherers: Application of the Necessity–Concerns Framework. *J Psychosom Res* 2008;**64**:41–6. doi:10.1016/j.jpsychores.2007.05.004

35 Moore G, Audrey S, Barker M, *et al.* Process evaluation of complex interventions UK Medical Research Council (MRC) guidance.

For peer review only

https://www.mrc.ac.uk/documents/pdf/mrc-phsrn-process-evaluation-guidance-final/ (accessed 3 Aug 2017).

36 Ryan RM, Deci EL. Self-determination theory and the facilitation of intrinsic motivation, social development, and well-being. *Am Psychol* 2000;**55**:68–78.http://www.ncbi.nlm.nih.gov/pubmed/11392867 (accessed 10 May 2019).

37 Baumeister H, Reichler L, Muzinger M, *et al.* The impact of guidance on Internet-based mental health interventions - A systematic review. *Internet Interv* 2014;**1**:205–15. doi:10.1016/j.invent.2014.08.003

38 O'Cathain A, Hoddinott P, Lewin S, *et al.* Maximising the impact of qualitative research in feasibility studies for randomised controlled trials: guidance for researchers. *Pilot Feasibility Stud* 2015;**1**:32. doi:10.1186/s40814-015-0026-y

39 Scholes S, Faulding S, Mindell J. Use of Prescribed Medicines. In: *Health Survey for England 2013: Health, Social Care and Lifestyles.* Leeds: : Health and Social Care Information Centre 2014. www.hscic.gov.uk/pubs/hse2013

ADvisor Guiding Principles	
Design objectives	*Key (distinctive) design features*
To **build confidence** that discontinuing antidepressant medication is safe and achievable over the long-term	• Offer an evidence-based rationale for how withdrawal and replacement with psychological./behavioural alternatives will help.<br>• Provide withdrawal success stories and examples (modelling).<br>• Address concerns patients may have re withdrawal (side effects, symptoms) from their previous experiences – demonstrate empathy and acknowledge real barriers to change.<br>• Offer motivational support.
To be an accessible, motivating resource that supports patients in managing their withdrawal in a	• Foster autonomy through choice and a non-prescriptive approach, providing explanations for all suggestions.<br>• Offer a broad range of strategies from

Table 1. Guiding Principles for the ADvisor intervention.

Digital intervention for antidepressant discontinuation

manner that aligns with **their preferences**	quick support in managing withdrawal symptoms, to more in-depth modules on a mindful approach to preventing depression relapse, and behavioural strategies for managing day-to-day stressors.<br>• Provide options for self-tailoring to personal experiences and barriers<br>• Provide a simple, attractive interface, with a focus on accessibly of content<br>•

1

2

peer review only

Digital intervention for antidepressant discontinuation

Table 2. Outline content of the digital intervention.

Content	Description
Reducing and stopping antidepressants	An introduction to the intervention, which addresses motivations behind withdrawal, asking participants to reflect on why they might prefer to discontinue antidepressant treatment. Guidance on when to speak to their GP/nurse and advice on following a tapering regime.
Thinking about antidepressants	Acknowledging that antidepressant treatment is not necessarily required long-term and that the mechanisms are more complex than correcting a serotonin deficiency.
I'm worried about stopping	Addressing participant fears by signposting participants to appropriate resources in ADvisor.
Dealing with withdrawal symptoms	Guidance for dealing with mild withdrawal symptoms (including guided practices for accepting/tolerating unpleasant symptoms). Advice for patients to contact their GP for assistance with moderate or severe withdrawal symptoms.
Keeping well	Relapse prevention techniques grounded in Mindfulness-Based Cognitive Therapy.
Thinking about what you value	Reflection on values and committed action to values (through goal setting), based on Acceptance and Commitment Therapy.
Moving forward	Psychoeducation and techniques for managing distress (e.g. mindfulness and behaviour activation) provided through a distress-management online intervention, Healthy Paths.
My Notes	Where patients can access content from other sections where they have written their own responses (for example their own reasons for wanting to stop antidepressants and their own warning signs and triggers for relapse).
Resources	Direct links to resources in ADvisor (e.g. activity planning and information for family and friends).

For peer review only

Digital intervention for antidepressant discontinuation

Characteristics	*N (%)*
Females	9 (60)
Males	6 (40)
Married	11 (73.3)
cohabiting	2 (13.3)
Single	2 (13.3)
Employed	9 (60)
Not currently in employment	6 (40)

Table 3. Think aloud qualitative study characteristics.

Diagnosis	
Depression/low mood	9 (60)
Fibromyalgia	2 (13.3)
Unknown	2 (13.3)
Urethritis	1 (6.7)
Post Traumatic Stress Disorder	1 (6.7)
Successfully stopped before	8 (53.%)
Currently taking antidepressants	14 (93.3%)
	**Mean (SD)**
Age	55.20 (15.59)
Years on antidepressants	10.43 (7.27)
PHQ-9 score	4.53 (2.50)

Digital intervention for antidepressant discontinuation

Figure 1. Logic model ADvisor intervention alongside additional components

For peer review only

Digital intervention for antidepressant discontinuation

For peer review only

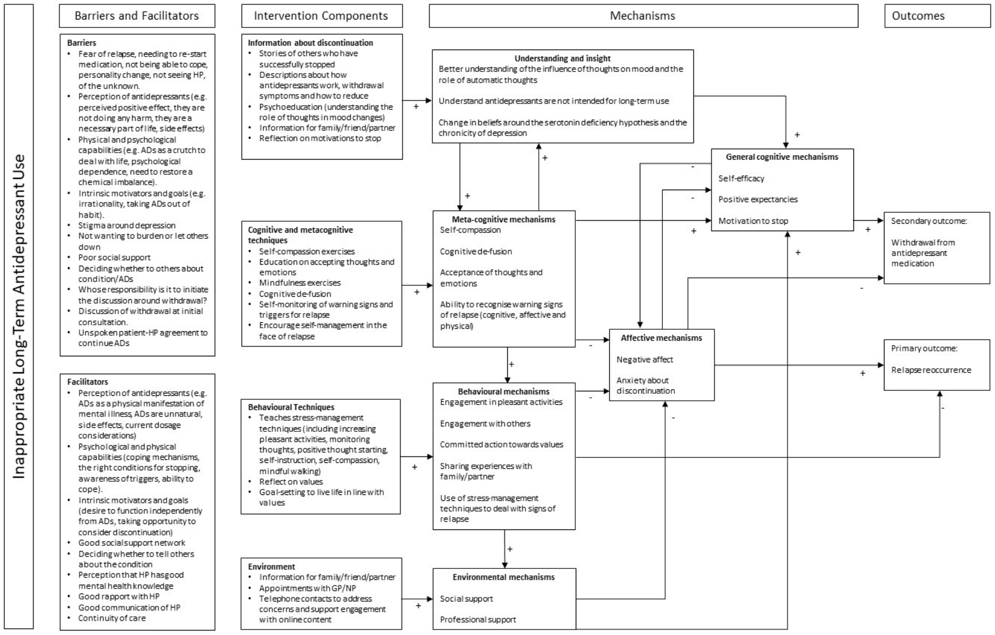

Figure 1. Logic model ADvisor intervention alongside additional components

355x266mm (96 x 96 DPI)

Appendix A – Interview Schedule

REDUCE

UNIVERSITY OF Southampton School of Medicine

REDUCE Study Workstream (WS) 3: REviewing long-term anti-Depressant treatment Use by Careful monitoring in Everyday practice

THINK-ALOUD INTERVIEW SCHEDULE WITH PATIENTS

Below is a list of topics/questions to be discussed in this study. The qualitative work will remain flexible with respect to participants' agendas but we will cover the broad topics/questions noted. It is common in qualitative work to iteratively develop topics and questions as new ideas emerge from early data collection. Therefore, we may add new topics as the interviews progress and data collection continues. However, the key topics of exploring participants' views of the prototype intervention will remain the same.

Introduction

1. Re-introduce self and purpose of interview

2. Check with participant:
- That they are still willing to be interviewed, and to be audio recorded
- Remind them it will take approximately 60 to 90 minutes
- That they are comfortable in a quiet place where they will not be disturbed

3. Remind participant that:
- Their responses will be kept confidential, and quotes used in the results will not identify them as an individual;
- They can change their mind about taking part in the study and stop the interview at any point.

4. Remind the participant that you will start by asking them some questions about their experiences with antidepressants. Remind the participant that you want them to look at the website and use it as they normally would, but say everything that they are thinking out loud. Tell them that you will remind them to do this so that they don't forget as it is very easy to forget and that there are no right or wrong answers as it is their views that are important to us.

5. Ask if the participant has any questions.

6. Start recording.

For peer review only - http://bmjopen.bmj.com/site/about/guidelines.xhtml

Section 1: Demographic Data

We would like to collect some personal information to help us describe the range of people / experiences we have collected, so could you please let me know your

Age	
Gender	M / F
Do you live alone or with someone (friends / partner / family)?	
Single / in a relationship / married?	
Employed / retired / full time carer / stay at home parent?	
Job title	
Currently on ADs?	Y / N
Successfully stopped ADs before?<br>NB. 'Success' = been off ADs & experienced symptom free episode(s).	Y / N
Same GP for review or different GPs within practice?	
Current Medical Diagnosis for ADs (if known)	
Do you pay for your prescriptions?	
Have you ever taken any sick leave from work due to depression / anxiety / stress? If yes, how much?	
Have you ever needed a carer/ or to be cared for due to depression? If yes, by whom?	
Any other medical conditions?	
Have you ever taken St John's Wort?	
Any other relevant information?	
Participant ID	
Date screened by researcher / confirm eligible	
Urban or rural location? (*researcher observation*)	
Deprivation level of area? (*researcher observation*)	

Section 2: Background history of use of antidepressants.

1. Can you tell me a little bit about when you were first prescribed antidepressants?

Prompt: Feelings about how decision to go on antidepressants was made/managed. Experience of taking ADs.

2. Could you describe your experience of taking antidepressants for me now?

Prompt: Any intent to stop? Have you found antidepressants have helped to improve your condition? Side effects/benefits? Expectations of ADs vs. lived experience.

3. Can you tell me about your current depression treatment?

Prompt:
- Regular repeat prescriptions?
- Any self-help or counselling / therapy?
- How often are you reviewed by a GP, nurse or counsellor/therapist? Feelings around frequency?
- Continuity of care?

For peer review only - http://bmjopen.bmj.com/site/about/guidelines.xhtml

- What treatment would you say has helped you most / least?

Section 3: Previous attempts to discontinue / successful withdrawal. Barriers and enablers to discontinuation (including individual / social factors).

1. Can you tell me about a time when you stopped or thought about stopping your antidepressants?

Prompt: What were your reasons for wanting to stop? How long did you stop for? What was it that made you stay on your antidepressants? Withdrawal experiences / effects. How would you feel if you had to restart your antidepressants or increase the dose (if stopped/stopping)? Explore expectations around withdrawal.

Section 4: Think-aloud and researcher prompts

Explain to them that you want them to look at the website and use it as they normally would, but say everything that they are thinking out loud. Tell them that you will remind them to do this so that they don't forget as it is very easy to forget. If you think it would help then get them to try counting the windows in their house whilst saying everything that they are thinking out loud.

- [only on first page] What are your first impressions of this page?
- What are you thinking now?
- What made you choose that option?
- What do you think about [this activity, this information]?
- Can you tell me a bit more about that?
- What is it you like about that?
- That's really interesting......

Section 5: Post-think-aloud questions

- Overall, what do you think about this website?
- Can you tell me about anything that you liked about the website?
- Was there anything that you found surprising in the website?
- Can you tell me anything about the website that you were less keen on?
- Can you tell me about anything that you think should be changed?
- What would you think if your GP or practice nurse asked you to use the website?
- If you were withdrawing from your antidepressants, which parts of AD-visor do you think you would like to look at and why? (E.g. dealing with withdrawal symptoms, information about how antidepressants work, relapse prevention, mindfulness etc.).
- When people use this website for real, they will be offered some support over the telephone. If you were using the programme for real, what would you think of this option to get support over the phone?
- What are your thoughts about telephone support throughout the trial in general? [Researcher to explain trial design].
- If you did have opportunity to have support over the telephone, which of the topics in AD-visor do you think would be most useful to discuss over the phone?

ANY OTHER TOPICS YOU WOULD LIKE TO DISCUSS?

ANY QUESTIONS?

For peer review only - http://bmjopen.bmj.com/site/about/guidelines.xhtml

Debrief
- Tell participant that the digital recorder is now being switched off.
- Thank participant for taking part in the interview.
- Revisit consent
- Ask if the participant has any questions about the study.
- Let them know that you will be sending all participants a summary of study findings.
- Check happy for data to be used for teaching / secondary analysis.
- Thank participant again for taking part in the interview.

For peer review only

For peer review only - http://bmjopen.bmj.com/site/about/guidelines.xhtml

Appendix B – Behavioural Diagnosis

Target behaviour: Reducing and stopping antidepressant medication		
**BCW/COM-B Components**	**What needs to happen for the target behaviour to occur?**	**Proposed intervention element**
**Physical capability**<br>*Physical skill, strength or stamina*	• Understanding how to reduce doses physically: e.g. how to take tapered medication appropriately, in order to reduce the occurrence of side effects.	• **GP**<br>• **Internet intervention modules**<br>• **Telephone support**
**Psychological capability**<br>*Knowledge or psychological skills, strength or stamina to engage in necessary mental processes*	• Detailed, accessible guidance on the withdrawal process in general (setting up appropriate expectations)<br>• Improving knowledge on how to withdraw (practicalities)<br>• Developing psychological skills to manage the process:<br>o Managing psychological side effects of withdrawal<br>o Understanding helpful appraisals of symptoms<br>o Learning about the prevention of relapse, managing fear of recurrence<br>o Developing skills to manage life-stressors cognitively and behaviourally<br><br>*Social Cognitive Theory (SCT) and research will be broadly drawn on to ensure information/techniques are described and applied to align with evidence-based principles for increasing self-efficacy*	• **Internet intervention modules**<br>• (Telephone support)

For peer review only

**Physical opportunity**<br>*Opportunity afforded by the environment involving time recourses, locations, cues, physical affordance*	• Ability to access and get to GP appointments/pharmacy to collect reduced dose antidepressants	• **General practitioner (as a function of usual care)**<br>• **Telephone support/advice**
**Social opportunity**<br>*Opportunity afforded by interpersonal influences, social cues and cultural norms that influence the way we think about things*	• Close social network (family/friends) of patient may need to be supportive of the withdrawal process/attempt	• **Brief overview material developed for family members/friends**
**Reflective motivation**<br>*Reflective processes involving evaluations/beliefs about what is good and bad, and plans (self-conscious intentions)*	• Modification of beliefs about depression:<br>o Exploring the nature of depression in a way that aligns with behavioural/cognitive management<br>o Discussing impact of beliefs and expectations about chronicity<br>o Exploring effect of analogies with physical conditions (diabetes/asthma)<br>o Acknowledging complexity re our understanding of depression in an accessible manner<br>• Modification of beliefs about antidepressant medication:<br>o Addressing beliefs about addiction/dependency<br>o Exploring the serotonin hypothesis; evidence, balanced implications, rationale for behaviour/cognition to substitute medication	• **Internet intervention modules**<br>• **Internet intervention modules**

For peer review only

	• Foster motivation to withdraw through discussion of benefits, reduction of side effects, potential for increase in agency, potential for effective use of alternatives to pharmacological management<br><br>• Facilitate clear planning for the withdrawal process e.g. human contacts, management strategies, access to rapid/emergency support<br><br>*Inductive qualitative work (meta-synthesis and primary qualitative research) and theory will be used to inform this material*	• **General practitioner**<br><br>• **Telephone support/advice**
**Automatic motivation**<br>*Automatic processes involving emotional reactions, desires (wants and needs) impulses, inhibitions, drive states and reflex responses*	• Encourage awareness of automatic disruptive modes/thought process that may trigger or be triggered by symptoms<br><br>• Work on developing habitual healthier responses to symptom occurrences	• **Internet intervention modules**
**Behavourial diagnosis of the relevant COM-B components**	Although all areas of the COM-B model will need to be addressed to some extent, **psychological capability** and **reflective motivation** are likely to be the key targets for a supported digital intervention to help patients withdraw from antidepressant medication	

References:

1. Michie S, van Stralen MM, West R. The behaviour change wheel: a new method for characterising and designing behaviour change interventions. Implement Sci. 2011;6:42.
2. Michie SF, Atkins L, West R. The behaviour change wheel: a guide to designing interventions. London: Silverback Publishing; 2015

For peer review only

Appendix C – Theoretical Modelling

**Intervention module**	**Page**	**Content**	**Evidence:** Importance of barrier/facilitator content targets OR evidence for effectiveness of content	**BCW construct**	**BCW function**	**BCTs (Taxonomy V1)** Techniques broadly applied across content sections	**SCT construct** Constructs applied across content sections	**NPT construct** Constructs applied across content sections
**Reducing and stopping antidepressants**	Welcome							
	Why should I reduce and stop?	Foster a motivation to withdraw through discussion of benefits, reduction of side effects, potential for increase in agency, potential for effective use of alternatives to medication	*Bosman et al. (2016); Dickinson et al. (2010); Verbeek-Heida and Mathot (2006); Iden et al. (2011); Karp (1993); Knudsen et al. (2002); Eveleigh (2015); Gibson (2016); Schofield (2011).*	Reflexive motivation	Enablement; training; education	9.1 Credible source<br><br>9.2 Pros and cons<br><br>15.2. Persuasion about capability<br><br>13.2 Framing-reframing	Knowledge; social outcome expectations; physical outcome expectations; Self-efficacy (Somatic and emotional states)	Coherence: Individual specification<br><br>Cognitive participation: Initiation
	The downsides	Reflection on the side effects of antidepressants as a means to foster motivation to withdraw		Reflexive motivation	Enablement; training; education			

For peer review only - http://bmjopen.bmj.com/site/about/guidelines.xhtml

	When should I reduce and stop?	Highlighting that it is best to start withdrawal at a stable time in life		Psychological capability	Enablement; training; education			
	What to expect	Outline the discontinuation process: that the GP will provide a schedule, that this is flexible and that there may be side effects but there are ways to manage these and they are often short-lived.		Psychological capability	Enablement; training; education			
	Addressing concerns	Briefly acknowledges that many people have concerns about withdrawal but that there are techniques for dealing with this in AD-visor		Psychological capability	Enablement; training; education			
	How can my GP help?	Outline the role of the GP in discontinuation,	*Bosman et al. (2016); Dickenson et al.*	Physical capability	Enablement; training; education			

		when to go to the GP for support.	*2010; Grime & Pollock (2003); Verbeek-Heida and Mathot (2006); Eveleigh (2015); Gibson (2016); Leydon et al. (2007) ; Cartwright (2016)*					
	Planning ahead	Overview of the process: GP will give schedule and as one tapers, there is support in AD-visor that can be used		Reflexive motivation	Enablement; training; education			
	Support from family and friends	Highlight how friends and family members can play and important role	*Bosman et al. (2016); Cromartry (2011); Verbeek-Heida and Mathot (2006); Eveleigh (2015)*	Social opportunity	Enablement; training; education	3.1 Social support 3.3 Social support (emotional)		
**How to reduce antidepressants**	How to reduce	Practical information about tapering schedules		Physical capability	Enablement; training; education	4.1 Instructions on how to perform behaviour	Self-efficacy (Mastery experiences/vic	Coherence: Individual specification

	How to reduce (2)	Highlight that there is unlikely to be a need for liquid formulations or pill cutters but if needed, the GP can offer some guidance (perhaps via community pharmacist)		Physical capability	Environmental restructuring; Enablement; training; education	6.1 Demonstration of behaviour (modelling)	arious experiences).	
	When to reduce	Reiterate that there are ideal times to begin tapering, such as when no major life events are expected		Psychological capability	Enablement; training; education			
**Thinking about antidepressants**	What are antidepressants?	Briefly explains what antidepressants are used for. Highlights that while it was believed they work through increasing serotonin, we now know it is more complex than that.	*Bosman et al. (2016); Dickenson et al. 2010; Grime & Pollock (2003); Verbeek-Heida and Mathot (2006); Karp (1993); Knudsen et al. (2002); Eveleigh (2015); Gibson (2016); Cartwright*	Reflexive motivation	Enablement; training; education	13.2 Framing/reframing<br><br>15.2. Persuasion about capability	Social outcome expectations; Knowledge; physical outcome expectations	Coherence: Internalisation

For peer review only

	Can I stop taking them?	Key point: even though we don't know exactly *how* they work, we do know that many people can successfully discontinue	*(2016); Leydon et al. (2007).*	Reflexive motivation	Enablement; training; education			
	Other forms of 'antidepressant'	There are things other than medication which can improve mood. The relationship between brain and behaviour is highlighted through a study which shows that CBT can result in changes in the brain		Reflexive motivation	Enablement; training; education			
	How to antidepressants work	Highlights again that we don't know exactly how they work but we do know: ADs help some people and not others and many		Reflexive motivation	Enablement; training; education			

		people can successfully stop.						
**I'm worried about stopping**	I'm worried about stopping	Highlight that many people have concerns about stopping and this is understandable and does not mean you won't be able to discontinue	Bosman et al. (2016); Dickinson et al. (2010); Verbeek-Heida and Mathot (2006); Iden et al. (2011); Karp (1993); Knudsen et al. (2002); Eveleigh (2015); Gibson (2016); Schofield (2011); Leydon et al. (2007).	Psychological capability	Enablement; training; education	13.2 Framing/reframing<br><br>15.2. Persuasion about capability	Knowledge, Self-efficacy (Mastery experiences vicarious experiences).<br><br>Social outcome expectations; Knowledge; physical outcome expectations	Cognitive participation: Initiation<br><br>Cognitive participation: Activation
	Successful stopping	Indicate that many people stop SD without problems, and those who are worried can overcome their concerns		Psychological capability	Enablement; training; education			
	Concerns about stopping	Patients will be given a selection of options to click on to read more about specific concerns		Psychological capability	Enablement; training; education			
	How will I cope if something big happens?	Reassure that AD-visor has guidance on managing stress in difficult		Psychological capability	Enablement; training; education			

For peer review only

		situations. Signpost to Moving Forward module.						
	What if I go back to how I was before?	Reassure that AD-visor has guidance on preventing relapse and signpost to Keeping Well module.		Psychological capability	Enablement; training; education			
	What if I have to start taking antidepressants again?	Reassure that hopefully this won't be necessary because they will learn how to prevent relapse, but if it is, they can try withdrawing again in future		Psychological capability	Enablement; training; education			
	How will I manage my responsibilities?	Guidance on planning activities and highlight the importance family support as well as the timing of the tapering process		Psychological capability	Enablement; training; education			

For peer review only

	Dealing with worries	Reflecting on the motivations to discontinue and weighing these up against concerns.		Reflexive motivation	Enablement; training; education			
**Keeping well**	Keeping well	Introduce to the idea of relapse prevention	Kuyken (2008); Allen (2009); Kuyken (2010); Fava (1998); Cromarty (2011); Otto (2010);	Psychological capability	Enablement; training; education	11.2 Reduce negative emotions<br><br>13.2 Framing/reframin g<br><br>6.1 Demonstration of behaviour<br><br>4.3 Reattribution	Knowledge, Goals Self-efficacy (Mastery experiences vicarious experiences).<br><br>Social outcome expectations; Knowledge; physical outcome expectation	Cognitive participation: Activation
	Automatic pilot	Define running on autopilot and explain negative automatic thoughts		Psychological capability	Enablement; training; education			
	The power of thoughts	Explain how the way we think impacts mood and teach cognitive defusion (thoughts are not facts)		Psychological capability	Enablement; training; education			
	Let it be	Defining the term 'acceptance' and why it is useful in dealing		Psychological capability	Enablement; training; education			

		with difficult thoughts and feelings						
	Recognising warning signs	Explaining and reflecting on what thoughts and physical sensations might be indicators of relapse		Psychological capability	Enablement; training; education			
	Recognising triggers	Reflecting on situations that might trigger a relapse		Psychological capability	Enablement; training; education			
	Recognising relapse	Writing down warning signs and triggers and saving these to view later		Psychological capability	Enablement; training; education			
	Responding differently	Highlight that you cannot change thoughts or the things that happen in life, but you have a choice how to respond to these. Responding in more helpful		Psychological capability	Enablement; training; education			

		ways can prevent relapse.						
	Preventing relapse	1. Take a breath<br>2. Make a decision on how to act<br>3. Take action		Psychological capability	Enablement; training; education			
**Living life with values and goals***	What are values	Defines values as like a compass point providing direction for our lives.	Swain et al. 2013; Powers et al. 2009.	Psychological capability	Enablement; training; education	11.2 Reduce negative emotions<br><br>13.2 Framing/reframin g<br><br>6.1 Demonstration of behaviour	Knowledge, Goals	Coherence: Internalisation
	What do I value?	Provides a space to write down what they value		Psychological capability	Enablement; training; education			
	Goals	Explaining the need to set goals in order to		Psychological capability	Enablement; training; education			

		act in line with our values				4.3 Reattribution		
	Setting goals	Guidance and space to write goals		Psychological capability	Enablement; training; education			
	Meeting goals	Reminds users to revisit this section to review their goals and see if they have met them		Psychological capability	Enablement; training; education			
**Dealing with withdrawal symptoms**	What are withdrawal symptoms?	Describes what they are and that they are a consequence of the brain and body adapting to the change in medication	*Bosman et al. (2016); Dickinson et al. (2010); Verbeek-Heida and Mathot (2006); Iden et al. (2011); Karp (1993); Knudsen et al. (2002); Eveleigh (2015); Gibson (2016); Schofield (2011); Leydon et al. (2007)*	Psychological capability Physical capability	Enablement; training; education	13.2 Framing/reframing<br><br>6.1 Demonstration of behaviour<br><br>4.3 Reattribution	Social outcome expectations; Knowledge; physical outcome expectations	Cognitive participation: Activation
	Recognising withdrawal symptoms	This page highlights that there are different symptoms that might be physical or mental. Specific details of what symptoms may occur are not given.		Psychological capability Physical capability	Enablement; training; education			

	Thinking about withdrawal symptoms	Explains that the way we think about symptoms can change how much impact they have (e.g. if you mistake a withdrawal symptom for relapse, it may be harder for the symptom to pass).		Psychological capability Physical capability	Enablement; training; education			
	Knowing the difference	Details about the differences between withdrawal symptoms and relapse.		Psychological capability Physical capability	Enablement; training; education			
	Dealing with withdrawal symptoms	Mild symptoms can be tolerated and will pass, moderate symptoms can be treated by a doctor, and severe symptoms may indicate a slower taper is needed.		Psychological capability Physical capability	Enablement; training; education			

	Accepting withdrawal symptoms	Guidance on accepting/tolerating symptoms based on acceptance and commitment exercises used with chronic physical symptoms		Psychological capability Physical capability	Enablement; training; education			
**Moving forward**	Healthy Paths Through Stress intervention (Healthy Paths). See Geraghty et al. 2017 for full description	This module is based on an intervention aimed at managing life stresses. The modules have been developed as part of a separate project and their content will be incorporated into AD-visor. This section will include guidance on mindfulness practices and behavioural activation.	Muñoz et al. 2005; Geraghty et al. 2016.	Psychological capability	Enablement; training; education	11.2 Reduce negative emotions<br><br>13.2 Framing/reframing<br><br>6.1 Demonstration of behaviour<br><br>4.3 Reattribution	Knowledge, Goals Self-efficacy (Mastery experiences vicarious experiences).<br><br>Social outcome expectations; Knowledge; physical outcome expectations	Coherence: Individual specification<br><br>Coherence: Internalisation<br><br>Cognitive participation: Initiation<br><br>Cognitive participation: Activation

For peer review only

New references added:

Bosman, R. C., Huijbregts, K. M., Verhaak, P. F. M., Ruhé, H. G., Van Marwijk, H. W. J., Van Balkom, A. J. L. M., & Batelaan, N. M. (2016). Long-term antidepressant use: A qualitative study on perspectives of patients and GPs in primary care. *British Journal of General Practice*, *66*(651), e708–e719. https://doi.org/10.3399/bjgp16X686641

Cartwright, C., Gibson, K., & Read, J. (2016). Long-term antidepressant use : patient perspectives of benefits and adverse effects, 1401–1407. https://doi.org/10.2147/PPA.S110632

Cromarty, P., Jonsson, J., Moorhead, S., & Freeston, M. H. (2011). Cognitive Behaviour Therapy for Withdrawal from Antidepressant Medication: A Single Case Series. *Behavioural and Cognitive Psychotherapy*, *39*(01), 77–97. https://doi.org/10.1017/S1352465810000512

Dickinson, R., Knapp, P., House, A. O., Dimri, V., Zermansky, A., Petty, D., … Raynor, D. K. (2010). Long-term prescribing of antidepressants in the older population: A qualitative study. *British Journal of General Practice*, *60*(573), 257–259. https://doi.org/10.3399/bjgp10X483913

Eveleigh, R. H. (2015). *Inappropriate long-term antidepressant use in primary care: A challenge to change*. Radboud University Nijmegen. https://doi.org/10.1063/1.3033202

Fava, G. A., Rafanelli, C., Grandi, S., Conti, S., & Belluardo, P. (1998). Prevention of Recurrent Depression With Cognitive Behavioral Therapy. *Arch Gen Psychiatry*, *55*, 816–820.

Geraghty, A. W. A., Muñoz, R. F., Yardley, L., Mc Sharry, J., Little, P., & Moore, M. (2016). Developing an Unguided Internet-Delivered Intervention for Emotional Distress in Primary Care Patients: Applying Common Factor and Person-Based Approaches. *JMIR Ment Health*, *3*(4), e53.

Gibson, K., Cartwright, C., & Read, J. (2016). “In my life antidepressants have been..”: A qualitative analysis of users’ diverse experiences with antidepressants. *BMC Psychiatry*, *16*(1), 1–7. https://doi.org/10.1186/s12888-016-0844-3

Grime, K. P. J. (2002). Understanding Depression and its Treatment: GP, Patient and Pharmacist Perspectives, (September), 52–60. Retrieved from http://www.pharmacyresearchuk.org/waterway/wp-content/uploads/2012/11/Understanding_depression_and_its_treatment1.pdf

Iden, K. R., Hjørleifsson, S., & Ruths, S. (2011). Treatment decisions on antidepressants in nursing homes: A qualitative study. *Scandinavian Journal of Primary Health Care*, *29*(4), 252–256. https://doi.org/10.3109/02813432.2011.628240

Karp, D. A. (1993). Taking anti-depressant medications: Resistance, trial commitment, conversion, disenchantment. *Qualitative Sociology*, *16*(4), 337–359. https://doi.org/10.1007/BF00989969

Knudsen, P., Hansen, E. H., Traulsen, J. M., & Eskildsen, K. (2002). Changes in Self-Concept While Using SSRI Antidepressants. *Qualitative Health Research*, *12*(7), 932–944. https://doi.org/10.1177/104973202129120368

For peer review only

Kuyken, W., Byford, S., Watkins, E., Holden, E., White, K., King 's, B. B., … Teasdale, J. D. (2008). Mindfulness-Based Cognitive Therapy to Prevent Relapse in Recurrent Depression. *Journal of Consulting and Clinical Psychology*, *76*(6), 966–978. https://doi.org/10.1037/a0013786

Kuyken, W., Watkins, E., Holden, E., White, K., Taylor, R. S., Byford, S., … Dalgleish, T. (2010). How does mindfulness-based cognitive therapy work? *Behaviour Research and Therapy*, *48*, 1105–1112. https://doi.org/10.1016/j.brat.2010.08.003

Leydon, G. M., Rodgers, L., & Kendrick, T. (2007). A qualitative study of patient views on discontinuing long-term selective serotonin reuptake inhibitors. *Family Practice*, *24*(6), 570–575. https://doi.org/10.1093/fampra/cmm069

Muñoz, R. F., & Mendelson, T. (2005). Toward evidence-based interventions for diverse populations: The San Francisco General Hospital prevention and treatment manuals. *J Consult Clin Psychol*, *73*(5), 790-799.

Otto, M. W., McHugh, R. K., Simon, N. M., Farach, F. J., Worthington, J. J., & Pollack, M. H. (2010). Efficacy of CBT for benzodiazepine discontinuation in patients with panic disorder: Further evaluation. *Behaviour Research and Therapy*, *48*(8), 720–727. https://doi.org/10.1016/J.BRAT.2010.04.002

Powers, M. B., Zum Vorde Sive Vording, M. B., & Emmelkamp, P. M. (2009). Acceptance and commitment therapy: a meta-analytic review. *Psychother Psychosom*, *78*(2), 73-80.

Schofield, P., Crosland, A., Waheed, W., Aseem, S., Gask, L., Wallace, A., … Tylee, A. (2011). Patients' views of antidepressants: From first experiences to becoming expert. *British Journal of General Practice*, *61*(585), 142–148. https://doi.org/10.3399/bjgp11X567045

Swain, J., Hancock, K., Hainsworth, C., & Bowman, J. (2013). Acceptance and commitment therapy in the treatment of anxiety: a systematic review. *Clin Psychol Rev*, *33*(8), 965-978.

Verbeek-Heida, P. M., & Mathot, E. F. (2006). Better safe than sorry — why patients prefer to stop using selective serotonin reuptake inhibitor (SSRI) antidepressants but are afraid to do so: results of a qualitative study. *Chronic Illness*, *2*(2), 133–142. https://doi.org/10.1179/174592006X111003

For peer review only

For peer review only

Standards for Reporting Qualitative Research (SRQR)*

http://www.equator-network.org/reporting-guidelines/srqr/

Page/line no(s).

Title and abstract

**Title** - Concise description of the nature and topic of the study Identifying the study as qualitative or indicating the approach (e.g., ethnography, grounded theory) or data collection methods (e.g., interview, focus group) is recommended	Page 1
**Abstract** - Summary of key elements of the study using the abstract format of the intended publication; typically includes background, purpose, methods, results, and conclusions	Page 2

Introduction

**Problem formulation** - Description and significance of the problem/phenomenon studied; review of relevant theory and empirical work; problem statement	Page 4 line 4-30
**Purpose or research question** - Purpose of the study and specific objectives or questions	Page 5 line 1-10

Methods

**Qualitative approach and research paradigm** - Qualitative approach (e.g., ethnography, grounded theory, case study, phenomenology, narrative research) and guiding theory if appropriate; identifying the research paradigm (e.g., postpositivist, constructivist/ interpretivist) is also recommended; rationale**	Page 7, line 17-19
**Researcher characteristics and reflexivity** - Researchers' characteristics that may influence the research, including personal attributes, qualifications/experience, relationship with participants, assumptions, and/or presuppositions; potential or actual interaction between researchers' characteristics and the research questions, approach, methods, results, and/or transferability	Page 22 line 1-7
**Context** - Setting/site and salient contextual factors; rationale**	Page 8 line 1-17
**Sampling strategy** - How and why research participants, documents, or events were selected; criteria for deciding when no further sampling was necessary (e.g., sampling saturation); rationale**	Page 7 line 22-31<br>Page 8 line 14-17
**Ethical issues pertaining to human subjects** - Documentation of approval by an appropriate ethics review board and participant consent, or explanation for lack thereof; other confidentiality and data security issues	Page 7 line 19-20
**Data collection methods** - Types of data collected; details of data collection procedures including (as appropriate) start and stop dates of data collection and analysis, iterative process, triangulation of sources/methods, and modification of procedures in response to evolving study findings; rationale**	Page 8 line 1-38

For peer review only

For peer review only - http://bmjopen.bmj.com/site/about/guidelines.xhtml

**Data collection instruments and technologies** - Description of instruments (e.g., interview guides, questionnaires) and devices (e.g., audio recorders) used for data collection; if/how the instrument(s) changed over the course of the study	Page 8 line 1-17
**Units of study** - Number and relevant characteristics of participants, documents, or events included in the study; level of participation (could be reported in results)	Page 30 Table 3
**Data processing** - Methods for processing data prior to and during analysis, including transcription, data entry, data management and security, verification of data integrity, data coding, and anonymization/de-identification of excerpts	Page 8 line 1-17
**Data analysis** - Process by which inferences, themes, etc., were identified and developed, including the researchers involved in data analysis; usually references a specific paradigm or approach; rationale**	Page 8 line 20-33
**Techniques to enhance trustworthiness** - Techniques to enhance trustworthiness and credibility of data analysis (e.g., member checking, audit trail, triangulation); rationale**	Page 8 line 27-29

Results/findings

**Synthesis and interpretation** - Main findings (e.g., interpretations, inferences, and themes); might include development of a theory or model, or integration with prior research or theory	Page 13 line 27 to page19 line 22
**Links to empirical data** - Evidence (e.g., quotes, field notes, text excerpts, photographs) to substantiate analytic findings	Page 13 line 27 to page19 line 22

Discussion

**Integration with prior work, implications, transferability, and contribution(s) to the field -** Short summary of main findings; explanation of how findings and conclusions connect to, support, elaborate on, or challenge conclusions of earlier scholarship; discussion of scope of application/generalizability; identification of unique contribution(s) to scholarship in a discipline or field	Page 19 line 26 to Page 20 line 10
**Limitations** - Trustworthiness and limitations of findings	Page 20 line 18 to page 21 line 7

Other

**Conflicts of interest** - Potential sources of influence or perceived influence on study conduct and conclusions; how these were managed	Page 22 line 11-16
**Funding** - Sources of funding and other support; role of funders in data collection, interpretation, and reporting	Page 22 line 2-3

*The authors created the SRQR by searching the literature to identify guidelines, reporting standards, and critical appraisal criteria for qualitative research; reviewing the reference lists of retrieved sources; and contacting experts to gain feedback. The SRQR aims to improve the transparency of all aspects of qualitative research by providing clear standards for reporting qualitative research.

For peer review only - http://bmjopen.bmj.com/site/about/guidelines.xhtml

**The rationale should briefly discuss the justification for choosing that theory, approach, method, or technique rather than other options available, the assumptions and limitations implicit in those choices, and how those choices influence study conclusions and transferability. As appropriate, the rationale for several items might be discussed together.

Reference:

O'Brien BC, Harris IB, Beckman TJ, Reed DA, Cook DA. **Standards for reporting qualitative research: a synthesis of recommendations.** *Academic Medicine*, Vol. 89, No. 9 / Sept 2014 DOI: 10.1097/ACM.0000000000000388

For peer review only

For peer review only - http://bmjopen.bmj.com/site/about/guidelines.xhtml

BMJ Open

Supporting antidepressant discontinuation: The development and optimisation of a digital intervention for patients in UK primary care using a theory-, evidence-, and person-based approach

Journal:	*BMJ Open*
Manuscript ID	bmjopen-2019-032312.R2
Article Type:	Original research
Date Submitted by the Author:	20-Dec-2019
Complete List of Authors:	Bowers, Hannah; University of Southampton, Primary Care, Population Sciences and Medical Education<br>Kendrick, Tony; University of Southampton, Primary Care, Population Sciences and Medical Education<br>Glowacka, Marta; Bournemouth University, Faculty of Health and Social Sciences<br>Williams, Samantha; University of Southampton, Primary Care, Population Sciences and Medical Education<br>Leydon, Geraldine; University of Southampton, Primary Care, Population Sciences and Medical Education<br>May, Carl; London School of Hygiene and Tropical Medicine Faculty of Epidemiology and Population Health<br>Dowrick, CF; University of Liverpool,<br>Moncrieff, Joanna; University College London and North East London mental health trust, Mental Health Sciences<br>Laine, Rebecca; University of Southampton, Primary Care, Population Sciences and Medical Education<br>Nestoriuc, Yvonne; Helmet Schmidt University, Department of Psychology<br>Andersson, Gerhard; Linkoping University; Karolinska Institutet, Department of Clinical Neuroscience and Psychiatry<br>Geraghty, Adam; University of Southampton, Primary Care, Population Sciences and Medical Education
**Primary Subject Heading**:	Mental health
Secondary Subject Heading:	Mental health, General practice / Family practice
Keywords:	Depression & mood disorders < PSYCHIATRY, Intervention Development, Digital Intervention, Antidepressants, PRIMARY CARE

SCHOLARONE™ Manuscripts

I, the Submitting Author has the right to grant and does grant on behalf of all authors of the Work (as defined in the below author licence), an exclusive licence and/or a non-exclusive licence for contributions from authors who are: i) UK Crown employees; ii) where BMJ has agreed a CC-BY licence shall apply, and/or iii) in accordance with the terms applicable for US Federal Government officers or employees acting as part of their official duties; on a worldwide, perpetual, irrevocable, royalty-free basis to BMJ Publishing Group Ltd ("BMJ") its licensees and where the relevant Journal is co-owned by BMJ to the co-owners of the Journal, to publish the Work in this journal and any other BMJ products and to exploit all rights, as set out in our licence.

The Submitting Author accepts and understands that any supply made under these terms is made by BMJ to the Submitting Author unless you are acting as an employee on behalf of your employer or a postgraduate student of an affiliated institution which is paying any applicable article publishing charge ("APC") for Open Access articles. Where the Submitting Author wishes to make the Work available on an Open Access basis (and intends to pay the relevant APC), the terms of reuse of such Open Access shall be governed by a Creative Commons licence – details of these licences and which Creative Commons licence will apply to this Work are set out in our licence referred to above.

Other than as permitted in any relevant BMJ Author's Self Archiving Policies, I confirm this Work has not been accepted for publication elsewhere, is not being considered for publication elsewhere and does not duplicate material already published. I confirm all authors consent to publication of this Work and authorise the granting of this licence.

For peer review only

Supporting antidepressant discontinuation: The development and optimisation of a digital intervention for patients in UK primary care using a theory-, evidence-, and person-based approach

Hannah M Bowers*[1], Tony Kendrick[1], Marta Glowacka[2], Samantha Williams[1], Geraldine M Leydon[1], Carl May[3], C F Dowrick[4], Joanna Moncrieff[5], Rebecca Laine[1], Yvonne Nestoriuc[6], Gerhard Andersson[7,8], Adam W A Geraghty[1].

[1] Primary Care, Population Sciences and Medical Education, University of Southampton, UK.

[2] Faculty of Health and Social Sciences, Bournemouth University, UK.

[3] Department of Health Services Research and Policy, London School of Hygiene and Tropical Medicine, UK

[4] Institute of Population Health Sciences, University of Liverpool, UK.

[5] Division of Psychiatry, University College London, UK.

[6] Department of Psychology, Helmut Schmidt University, Germany.

[7] Department of Behavioural Sciences and Learning, Linkoping University, Sweden.

[8] Department of Clinical Neuroscience and Psychiatry, Karolinska Institutet, Sweden.

…

For peer review only

Digital intervention for antidepressant discontinuation

*Corresponding Author: H.M.Bowers@soton.ac.uk. Primary Care and Population Sciences, University of Southampton, Aldermoor Health Centre, Aldermoor Close, Southampton, SO15 5HT. Tel: 02380 591743

…

For peer review only

Abstract

Objectives: We aimed to develop a digital intervention to support antidepressant discontinuation in UK primary care that is scalable, accessible, safe and feasible. In this paper we describe the development using a theory- evidence- and person-based approach.

Design: Intervention development using a theory-, evidence-, and person-based approach

Setting: Primary Care in the South of England

Participants: Fifteen participants with a range of antidepressant experience took part in 'think aloud' interviews for intervention optimisation

Intervention: Our digital intervention prototype (called 'ADvisor') was developed on the basis of a planning phase consisting of qualitative and quantitative reviews, an in-depth qualitative study, the development of guiding principles and a theory-based behavioural analysis. Our optimisation phase consisted of 'think aloud' interviews where the intervention was iteratively refined.

Results: The qualitative systematic review and in-depth qualitative study highlighted the centrality of fear of depression relapse as a key barrier to discontinuation. The quantitative systematic review showed that psychologically informed approaches such as cognitive

Digital intervention for antidepressant discontinuation

behaviour therapy (CBT) were associated with greater rates of discontinuation than simple advice to reduce. Following a behavioural diagnosis based on the Behaviour Change Wheel, Social Cognitive Theory provided a theoretical basis for the intervention. The intervention was optimised on the basis of think aloud interviews, where participants suggested they like the flexibility of the system and found it reassuring. Changes were made to the tone of the material and the structure was adjusted based on this qualitative feedback.

Conclusions: 'ADvisor' is an evidence-, theory- and person-based digital intervention designed to support antidepressant discontinuation. The intervention was perceived as helpful and reassuring in optimisation interviews. Trials are now needed to determine the feasibility, clinical and cost effectiveness of this approach.

278 word (BMJOpen limit 300).

For peer review only

Strengths and Limitations of the study

- A systematic review and qualitative meta-synthesis were conducted alongside primary qualitative work to guide the content of the intervention.
- A theory-based behavioural analysis and the development of guiding principles further informed the planning phase of intervention development.
- Think aloud interviews provided in-depth understanding of patients' views of the intervention in terms of usability and content.
- The intervention was iteratively refined throughout the think aloud interviews to produce an intervention that aligns with patient preference.
- Think aloud participants were predominantly White British and from more affluent regions in the South of England and may not represent the views of all antidepressant users.

For peer review only

Introduction

The number of antidepressant prescriptions in the UK has continued to rise over the past four decades [1], a trend which has also been seen in the United States and across Europe [2,3]. Approximately 10% of adults in the UK are currently prescribed antidepressant medication [4]. Though antidepressants can prevent relapse, there is evidence that 30-50% of patients on long-term antidepressants have no indication based on guidelines for long-term use [5–7]. Research suggests this increase in prescribing is primarily due to general practitioners (GPs) prescribing antidepressants for longer and longer durations over time [8]. Long-term antidepressant use is both costly to the UK National Health Service (NHS) (in terms of prescription and appointment costs) and is associated with increased side effects [9]. Attempting to discontinue antidepressants in the 30-50% with no indication for long-term use may therefore be beneficial to patients and positively impact on use of health-care resources.

There are many factors that may contribute to long-term antidepressant use, including the occurrence of a physiological withdrawal syndrome following reduction or cessation and psychological factors such as beliefs about the necessity of long-term use and fear of relapse [10]. Infrequent reviews of patients taking antidepressants may also contribute to sustained use [11]. However, simply prompting for patient reviews has resulted in discontinuation rates of 6-8%, not

For peer review only - http://bmjopen.bmj.com/site/about/guidelines.xhtml

significantly differing from usual care [12,13]. This highlights the potential importance of psychologically informed interventions to support withdrawal.

Trials have shown that Cognitive Behavioural Therapy (CBT) and Mindfulness-Based Cognitive Therapy (MBCT) can effectively support discontinuation of antidepressants, with cessation rates ranging from between 55%-95% [14–18]. Although producing positive outcomes, these interventions involve intensive group/face-to-face courses, thus access and ability to scale up within resource-strapped health services may be severely limited. There is a need for accessible, scalable psychologically-informed interventions that can effectively support individuals where discontinuation is appropriate.

In the UK, 89% of the general population in 2018 used the internet weekly, up from 55% in 2006 [19]. Internet-based digital interventions supported with human contact have been shown to effectively reduce depression and anxiety [20]. Digital intervention may have potential to provide a scalable, accessible way of supporting appropriate antidepressant discontinuation. We aimed to develop such a supported digital intervention as part of the UK-based REDUCE (REviewing long term antiDepressant Use by Careful monitoring in Everyday practice) programme to develop and trial safe, feasible and effective ways to support patients withdrawing from antidepressants where appropriate.

For peer review only

For peer review only - http://bmjopen.bmj.com/site/about/guidelines.xhtml

Digital intervention for antidepressant discontinuation

In this paper we describe the planning and optimisation of our patient-facing digital intervention to support discontinuation, named ‘ADvisor’. This paper provides an overview of the different stages of development and how these together informed a digital intervention. Some of this work has implications beyond intervention development and further details are therefore published elsewhere. This paper is instead focused on the particular work involved in developing a digital intervention.

Methods

Phase 1: Intervention planning and development

There is a range of systematic protocols for intervention development that can be drawn on at the outset of a development project (e.g. Intervention Mapping [21]). We chose to implement a theory-, evidence- and person-based approach [22]. This comprehensive strategy integrates the person-based approach (PBA) [23,24] with more commonly used theory and evidenced-based methods. The PBA provides guidance for integrating systematic in-depth qualitative research into the development process. Drawing on the PBA ensures evidence and theory-based techniques are applied with a full understanding of the target users’ perspectives and psychosocial context [23]. We will outline the components of our comprehensive

For peer review only - http://bmjopen.bmj.com/site/about/guidelines.xhtml

approach including systematic reviewing, primary qualitative research, development of guiding principles, behavioural analysis and logic modelling.

Systematic reviewing

Two systematic reviews were conducted: a quantitative review with meta-analysis, and a qualitative thematic synthesis, described in detail elsewhere [10,25]. The qualitative review searched nine databases from inception to February 2017 and updated searches were carried out in July 2018. Citation searching, reference list checking and related article checking was also performed. The quantitative review involved searching eight databases from inception to March 2017. Citations and reference lists were searched for full papers that met the inclusion criteria. Both searches were developed by an experience librarian and systematic reviewer. Further details of the search strategies can be found in the full publications of these reviews [10,25].

For intervention planning, from the quantitative review we drew out interventions that had successfully supported discontinuation and considered their intervention components, seeking full manuals where possible. We aimed to determine which components could be best translated into a digital format. In the qualitative review we identified barriers and facilitators to antidepressant discontinuation. Barriers and facilitators were tabulated and used to inform the 'Guiding Principles' (see below) as well as content for the intervention.

For peer review only

Digital intervention for antidepressant discontinuation

Primary qualitative research

Individual semi-structured interviews were conducted by SW with primary care patients with varying experiences of antidepressants, and varying levels of motivation to stop, with the aim to explore experiences of antidepressant discontinuation. These interviews explored patients' views on barriers and facilitators to withdrawal, the role of health care professionals in supporting withdrawal attempts, and elements of a proposed intervention to support withdrawal. Interviews were conducted at the patients' homes or their GP practices and were audio recorded and transcribed verbatim. Patients provided written consent. Analysis was conducted following thematic analytic principles suggested by Braun and Clarke [26], and Joffe and Yardley [27]. Analysis was conducted by SW (a qualitative researcher). The coding manual and developed themes were discussed and agreed by the wider development group. Only the findings related to the development of the intervention are described in this paper. Further details of the methods and the findings related to the broader aims of this piece of qualitative work will be published elsewhere.

Development of guiding principles

Guiding principles are a fundamental part of the PBA [23]. They represent broad design objectives that guide the application/implementation of the core intervention strategies, aiming to increase engagement [24]. Guiding principles were developed based on the qualitative synthesis [10] and primary qualitative findings. Through this

For peer review only - http://bmjopen.bmj.com/site/about/guidelines.xhtml

qualitative work we aimed to identify key behavioural needs, challenges or issues the intervention needed to address.

Behavioural analysis

Behavioural and implementation theory was drawn on as we triangulated between the qualitative and quantitative evidence, and the expert views of our team (including patient representatives, GPs, psychiatrists, psychologists, sociologists and health services researchers) to determine important intervention components. Using the Behaviour Change Wheel and COM-B model of behavior (Capability, Opportunity, Motivation – Behaviour) [28], informed by our qualitative research, we conducted a 'behavioural diagnosis' [29]. In behavioural diagnosis, factors that are likely to affect the central target behaviour are considered in terms of capability, opportunity, and motivation [28,29]. Once we had proposed initial intervention content/components, these were mapped theoretically using the Behaviour Change Wheel, Social Cognitive Theory (SCT) [30] and Normalisation Process Theory [31]. As well as providing a mapped full description of the proposed intervention, this process ensured we did not miss areas of theory that may have improved the intervention.

Phase 2: Intervention optimisation

Design

For peer review only - http://bmjopen.bmj.com/site/about/guidelines.xhtml

Digital intervention for antidepressant discontinuation

Within the PBA, 'think aloud' qualitative studies are employed to optimise the prototype intervention. Think aloud studies are designed to elicit in-depth perspectives about the nature of the content, rather than solely focusing on functionality and usability. Ethical approval for the study was granted by NHS South Central Oxford B Research Ethics Committee.

Participants

Participants were recruited from eight primary care practices in the South of England. Eligibility criteria were as follows: Inclusion criteria: Taking antidepressants for more than one year for a first episode or two years for a subsequent episode; discontinued antidepressants, or were in the process of tapering. Exclusion criteria: PHQ-9 scores greater than or equal to 10 (suggesting persisting symptoms of depression) and those who reported any suicide ideation; history of suicide attempts; ongoing social difficulties or recent life events likely to provoke relapse; more than three previous significant episodes of depression; comorbid psychosis, bipolar disorder, obsessive-compulsive disorder, or substance use (or past history of these conditions); or currently receiving psychiatric treatment.

Procedure

Eligible participants met with a researcher (HB, SW or TK) either in their own home or at their primary care practice where they provided written consent to take part in a think-aloud interview. Interviews invited participants to engage with the prototype

For peer review only

intervention using a study laptop and say what they were thinking, aloud in real time. The interviewer prompted participants when necessary (for example asking patients 'How do you feel about the information on this page?'). Interviews ranged from 38 to 93 minutes in length and were audio recorded, and transcribed verbatim. The interview ended when patients concluded they had looked at all the information they would like to see or if the interview length was approaching 90 minutes. The amount of intervention content the patient saw therefore depended on their own preferences and the time they took to look at the information. The interview schedule can be found in Appendix A. There were three primary iterations of interviews based on three key modified prototype interventions. Patients at the start of the study therefore saw different versions of the intervention to those who were recruited later rounds. This allowed the changes made as a result of patient feedback to continue to be tested. Interviews with patients continued until data saturation was reached, defined here as when comments about the intervention reflected that no further changes were necessary according to the person-based approach and when there were no new codes identified as part of the thematic analysis.

Analysis

Transcribed interviews were analysed using two primary analytic methods. The first analytic method was a more rapid coding than thematic analysis, which involves using coding tables designed for the PBA, where positive and negative comments were tabulated. Core problematic issues likely to affect participant engagement or

For peer review only

intervention effectiveness identified using this coding method were brought to the broader group, and amendments to the intervention agreed. Alongside this method, a more in-depth thematic analysis [26,27] was developed to capture patient views of the intervention and ideas about how they might engage with it, beyond comments on what might be amended. For this latter analysis, HB independently coded the transcripts and discussed a preliminary coding frame with a second researcher (AG). Theme labelling and interpretation were discussed and agreed by the team. The thematic analysis is presented here. Therefore while the initial analysis informed what changes were necessary, the thematic analysis explored what patients thought about the intervention in greater depth. These analyses were related in that some things that were identified in our initial analysis informed the development of themes.

Results

Phase 1: Intervention planning and development

Systematic reviewing

Our qualitative thematic synthesis (see [10] for full results) across 22 studies highlighted key barriers and facilitators to discontinuation. Patients' concerns regarding their ability to cope and psychological dependence were common barriers,

For peer review only - http://bmjopen.bmj.com/site/about/guidelines.xhtml

as were difficulties experienced in previous stopping attempts. Confidence in abilities to stop, effective coping strategies and stable life circumstances facilitated discontinuation. Additional important themes included fear of relapse – this was the central fear that prohibited stopping attempts – and beliefs about depression. The belief that depression was a long-term condition caused by biochemical changes in the brain was a key barrier to discontinuation. Where patients reported a very different belief, that depression was due to changing life circumstances, this seemed to facilitate discontinuation. Patients' self-identity and goals were an important factor: Having self-identifying as "old" or "disabled" acted as a barrier to discontinuation, and having goals to function independently functioned as facilitator to discontinuation.

In the quantitative systematic review (see [25] for full results) a variety of therapeutic techniques were implemented including a patient-specific letter to the GP with a recommendation to discontinue plus tapering advice; GP review of the patient's condition and medication; CBT plus tapering; MBCT with tapering support gradual discontinuation and one-week tapering. The results indicated that CBT or MBCT plus tapering are helpful for patients discontinuing antidepressants, with cessation rates of 40-95% [23], compared to only 6-8% cessation where health professionals are simply prompted to review patients. CBT plus tapering resulted in lower relapse rates compared with clinical management plus taper (15-25% vs 35-80%) [23]. The content of the interventions were extracted and feasibility of delivery in a digital

For peer review only - http://bmjopen.bmj.com/site/about/guidelines.xhtml

format was considered. We developed a module based closely on MBCT protocols on the basis of this review.

The findings from both reviews' findings informed the guiding principles, behavioural analysis and logic model, which formed the basis for intervention content selection and development.

Primary qualitative research

Five themes were developed through the thematic analysis of 19 patient interviews (full details will be published elsewhere). A summary is presented here. Participants spoke of the centrality of personal medication and health care factors, for example some patients described the need for a personalised tapering regime to support them discontinuing. Beliefs about depression and its treatment were key in shaping participants' stance towards discontinuing. For example, ideas around the necessity of anti-depressant medication due to 'chemical imbalance' were common. Holding these beliefs made patients less likely to consider stopping. Fear of stopping, driven by fear of relapse were discussed as central barriers to withdrawal. The impact of others also appeared to be important. For example, the perception of stigma and the feeling of letting people down, made participants less willing to discontinue, while having a good support network was considered beneficial to stopping. Participants were also asked to consider digital methods of intervention delivery. Elements participants wanted to see in the intervention included explanation around how

For peer review only - http://bmjopen.bmj.com/site/about/guidelines.xhtml

antidepressants work, support for anxiety/fear of discontinuing, coping strategies and information on withdrawal symptoms. There was some concern around privacy and around preference for greater face-to-face interaction to support them during the discontinuation phase. Patients expressed a need to have accessible, interactive and information presented in an aesthetically pleasing way.

The full findings in our primary qualitative research mirrored and expanded the findings of our qualitative thematic synthesis. They fed into the guiding principles, behavioural analysis logic model and content for the intervention.

Guiding principles

On the basis of the qualitative work guiding principles were developed (comprised of design objectives and design features), see Table 1. We developed two broad design objectives: The first, regarding building confidence that discontinuing antidepressant medication is safe and achievable, was developed from prominent themes around fear of stopping, the need for confidence, and beliefs that antidepressant medications are needed long-term. The second objective, that the intervention should be an accessible, motivating resource that supports patients in managing their withdrawal in a manner that aligns with their preferences, was developed in response to the range of views and beliefs held about the nature of depression and why antidepressants were necessary. Design features that support both these objectives are listed in Table 1.

For peer review only

[Insert table 1 about here]

Behavioural analysis

Our behavioural diagnosis following the COM-B model can be found in Appendix B. Our target behaviour was reducing and stopping the taking of antidepressant medication. Based on our reviews, qualitative work and discussion amongst our broader team, psychological capability and reflective motivation were considered key constructs for changing the target behaviour. The results of our behavioural diagnosis are presented in Appendix B.

Following the drafting of module content and structure, we mapped content against 1) studies suggesting content would be important, 2) Behaviour Change Wheel (BCW) constructs, 3) Social Cognitive Theory (SCT), and 4) Normalisation Process Theory (NPT). See Appendix C for detailed theoretical mapping for our intervention content.

Fundamentally, SCT [32] underlies the approach taken in the intervention to facilitate behaviour change. We ensured content aligned with the principles of SCT on how best to increase patient's confidence that they will be able to safely stop antidepressants (e.g. drawing on persuasion, modeling and supporting performance exposure). We also focused on modifying outcome expectations e.g. increase

For peer review only

positive expectation that the recommended strategies are likely to support effective discontinuation. At a later stage in development, the Necessity Concerns Framework (NCF) [33] was considered. NCF was developed to explain the role of treatment beliefs on adherence behaviours. According to NCF, adherence to treatment is a function of patients' beliefs about the necessity of their medication and the concerns they have about it; high necessity beliefs and low concerns are likely to predict medication adherence [34]. In the context of antidepressant withdrawal, accordingly, we would need to reduce patients' beliefs about the necessity of the medication, highlight likely benefits of stopping, and reduce concern regarding the stopping process. All of these factors will ultimately impact on self-efficacy, hence the centrality of SCT in our theoretical modelling.

Logic modelling

Logic models represent proposed or hypothesised 'theories of change' outlining the problem/issue and barriers, ingredients mechanism, and how these may affect target outcomes [35]. We developed a draft logic model for the REDUCE patient intervention, drawing on theory, evidence and our person-based qualitative work, see Figure 1.

[Insert Figure 1 about here]

Outline intervention content

For peer review only

Digital intervention for antidepressant discontinuation

On the basis of our planning process, a prototype digital intervention was developed for patients taking antidepressants long-term (defined as more than one year for a first episode or more than two years following two or more episodes). The contents of the online intervention are described in Table 2. A digital intervention for health professionals (providing information and guidance on antidepressant reduction) was also developed as part of the REDUCE programme and is reported separately.

[Insert Table 2 about here]

Content was developed using findings from the reviews of the literature, primary qualitative work, behavioural analysis and logic modelling. In addition to online content, scheduled telephone support contacts with specialists trained in providing psychological support and email reminders were developed as part of the patient intervention.

When accessing the ADvisor intervention for the first time, users view a core module with the central rationale for stopping antidepressants; they can then access a menu with a range of further modules based on our planning work. Aligning with our guiding principles, users are advised that they can use ADvisor how and when they would like. It is their tool, to be used to support them in a way that is consistent with their needs, preferences and experience. Through this approach we aimed to maximise autonomous motivation [36].

For peer review only - http://bmjopen.bmj.com/site/about/guidelines.xhtml

Content for the online intervention was initially drafted by a member of the content development team (HB) before AG and MG and then wider team members offered their expertise and informed further development of the content. This iterative process continued until all team members were satisfied that the prototype intervention addressed key experiences, barriers and facilitators identified by the work from phase one and were in line with the guiding principles, theoretical modelling and logic model. The content was transferred into online pages in LifeGuide (www.lifeguideonline.org) and further amendments to the presentation were made by the team before moving forward to the optimisation phase.

Phase 2: Intervention optimisation

Of the 42 patients who returned a postal reply slip expressing interest, 11 were ineligible, nine could not subsequently be contacted, two later declined, and five expressed an interest only after data saturation had been reached. This resulted in a final sample of 15 patients (see Table 3 for sample characteristics).

[Insert Table 3 about here]

Iterations of Advisor

For peer review only

There were three rounds of iterations of the intervention during the think-aloud interviews. Patients in round one were shown the first prototype. Changes made to the version in round two included making the tone less formal, revising the introduction navigation and the wording to be more gentle. The ‘my notes’ section was also reorganized to be clearer and buttons to exit the intervention at the end of each module were removed to try to keep the patients on site for longer. In the version shown in round three some changes included further revision of the tone, some of the information was presented in a more aesthetically pleasing way and some links within the intervention to other modules were removed as these were confusing for patients.

Findings

Six themes were developed, namely: flexible use; familiarity with content; reassurance; utility of information; teaching of useful skills; and feeling supported. Patient identifiers and demographic information are presented below each quote, where round number refers to the iteration of the intervention that the patient saw.

Flexible use

Participants discussed how ADvisor could be used in different ways to suit the individual. When viewing the main menu page in ADvisor participants talked about how different sections would be more useful for them, and that some sections were not relevant for them at that particular time.

For peer review only

Dealing with withdrawal symptoms, I don't have any, so it's fine. That [keeping well and moving forward modules] I'm more interested in about because I think that's - for me, keeping well and moving forward is where I am and where I want to be.

[14/03/0001] [round 1]

Initial versions of the intervention included an introduction module within which participants could choose which of two options they would like to view first, though they would need to view both sections before moving onto the main menu. Some participants felt that this was in contradiction to the aim of choice and flexibility. We therefore modified the intervention so that the introduction was shorter and these two choices were moved to optional buttons in the main menu.

It's kind of saying you've really got to look at that one; otherwise, you will have flicked back through or I would have thought it might have been, if it's really flexible, user friendly, you might be allowed to skip that page because you could always revisit it again.

[01/01/0026] [round 1]

Participants not only varied in the topics they wanted to look at, but also in terms of the different exercises they would choose to engage with in ADvisor. Some

For peer review only - http://bmjopen.bmj.com/site/about/guidelines.xhtml

participants liked the idea of writing down their responses in ADvisor while others did not.

No. That's me. No, I'm very stoic and – just – I don't need to write it down, it's fine; I know what I'm doing, I'm fine, very much, I think.

[01/01/0005] [round 2]

I'd like to say that I would [write things down]; I think I probably would if I was – you know – really serious about it, because I like to write things down and if I haven't written it down, it can just go out of my brain. So I think, for me, it would be important to write that down.

[05/01/0022] [round 2]

Participants also discussed how ADvisor could be used in different ways. For example, it can be something used regularly, something one can pick up as and when necessary or it can be read through all in one go.

So it looks like you can use it when you want to but if you feel you're coping without, so it's not something you have to do all the time.

[05/01/0022] [round 2]

For peer review only

> *Yes, I would use it for future reference, as well, because you can always go backwards, can't you? With anything, I mean. If I ever came to a time where I was feeling down, I think, to go back on to something is to remind you. Because it's easy to forget.*
>
> *[13/01/0058] [round 3]*

Familiarity with content

Many of the participants referred to previous experience with psychological therapies or tools they have used in the past for their symptoms of depression. When reading cognitive-behavioural, acceptance and commitment, or mindfulness-based information in ADvisor, participants expressed a sense of familiarity with the terminology or messages they were presented.

> *Clicking on Breathing Space; that's very much mindfulness, isn't it? Yes, I like that, that's nice.*
>
> *[14/03/0001] [round 1]*

Some of the information about depression and antidepressants seemed to be obvious to a small number of participants who had pre-existing knowledge, but they understood that not all patients would have the same prior knowledge. One participant in particular who worked in healthcare found that much of the information was not new to her.

For peer review only - http://bmjopen.bmj.com/site/about/guidelines.xhtml

Digital intervention for antidepressant discontinuation

I'm obviously interested in reducing still further or coming off the antidepressants. ... See I don't think I can – I do know an awful lot about it and read a lot about it and very – sorry – but, you know, being in the business myself, it's all a bit Noddy to Big Ears.

[13/01/0033] [round 3] [works in healthcare]

Reassurance

Participants described a sense of fear around stopping antidepressants. This has been reported in previous qualitative studies of patient and health professional perspectives on stopping [10]. Participants in this study often reported feeling reassured by information in ADvisor. While participants differed in terms of which particular piece of information they found reassuring, some participants noted feeling reassured knowing that they could go back on their antidepressant if they felt necessary. Other participants found that knowing that withdrawal symptoms are often short-lived offered reassurance.

Well that's a good section because that is quite a worry, I think, for anybody wanting to come off them; it would worry me what would my side-effects be and how would I feel coming off them. So to actually – I mean I didn't know this – to actually say that they are often short lived and go away in a few days or weeks is quite encouraging, isn't it.

For peer review only

[04/01/0025] [round 3]

As fear of withdrawal symptoms was highlighted in the qualitative work, withdrawal symptoms were discussed at several points during the introduction module. However participants who were not initially concerned about withdrawal symptoms felt that this was setting an expectation for difficulty withdrawing. Whilst not minimising withdrawal-related problems, we therefore revised the language around concerns about withdrawal in the introduction.

> *Well it's very obvious withdrawal is a problem, looking at all the advice you can see to help you get over it, which – yes. There's a negative feeling there, if it's stressed to this degree on this program, then you're obviously expecting trouble.*

[10/03/0003]

Credibility of the information appeared to be important for participants. Participants liked to see the evidence base that was provided in ADvisor and in particular liked that it would be used within an NHS setting. The NHS affiliation seemed to provide a sense of reliability and credibility.

> *I'd be really pleased if they [GP/nurse] referred me to a website, especially if it was from the GP, because I think, well, it's backed up or supported by them.*

For peer review only

[14/03/0001] [round 1]

There was a balance that needed to be struck between portraying information as credible and maintaining a warm and friendly tone. Participants reported some of the information in ADvisor as sounding academic and reading like it could be used by practitioners. As a result, the tone was revised to be warmer and friendlier, while maintaining a sense of credibility.

> *It's just very business-like so very much like maybe something that a university would produce or maybe that a medical professional would share amongst themselves and your everyday person who's maybe not used to reading things in so much detail any more, sadly. It's quite dry.*

[14/03/0001] [round 1]

Utility of information

Participants described the information on withdrawal symptoms to be useful, in particular, some participants liked the information on how to distinguish between signs of relapse and withdrawal symptoms. One participant in particular expressed a shift in her views on discontinuing as a result of the information in ADvisor. She explained that had she known that withdrawal symptoms may feel like relapse and will pass, she may have persisted with her lower dose of antidepressant for longer.

For peer review only

Digital intervention for antidepressant discontinuation

She also highlighted that difficulty in getting a GP appointment is a barrier for her to persist with discontinuing in the face of difficulties.

> *.. I didn't know ... withdrawal symptoms might appear the same as the symptoms that led to needing antidepressants in the first place, but they will pass after a short time; I didn't know that. I thought if you started feeling down again, then you were heading for a crash.*
>
> *[13/03/0001] [round 2]*

Some participants described wanting more detailed information about what withdrawal symptoms might be expected. However, upon discussion with the broader study team, it was decided to avoid setting expectations around particular symptoms as this may lead patients to experience expected symptoms. Patients can instead request this information from their GP if it is something they feel they would rather know about. While this information is provided to GPs as part of our health professional intervention package, it must be acknowledged that there are limitations around access to GP appointments which may act as a barrier to getting information about withdrawal symptoms.

Participants also noted that it was useful to reflect on the side effects of taking antidepressants. There was an awareness that these can be hard to recognise, and three participants reported that after reading the information in ADvisor, they may in

For peer review only - http://bmjopen.bmj.com/site/about/guidelines.xhtml

fact have been experiencing side effects of which they were previously unaware. One participant described how this made him even more inclined to discontinue.

Well, as I look at these, I think maybe I'm wrong; maybe I am still getting side-effects, but I've just learned to accept them or – I'm just a little bit in denial and it makes me want to get off them even more, because then – lots of these things will, you know, will disappear.

[12/03/0003] [round 1]

Teaching of useful skills

Participants reported the skills included in ADvisor as being useful. In particular, advice around preventing relapse and mindfulness-based skills were considered to be useful.

Your triggers, recognising your emotions and reminding yourself that you don't have to react in a certain way; you can react in a different way. Yes, I think it's very good.

[13/01/0001] [round 2]

For peer review only - http://bmjopen.bmj.com/site/about/guidelines.xhtml

Digital intervention for antidepressant discontinuation

Acceptance of difficulties and of emotions was discussed as a useful coping strategy by participants, both with regards to their own pre-existing relationship to their emotions, and with regards to the messages in ADvisor on acceptance.

> *When you read it like that, it is true; the more you worry about things, the more down you get. So you've got to learn to stop doing that. I have to start putting that into practice if I'm going to do this.*
>
> *[13/01/0058] [round 3]*

Participants liked having tools and techniques in ADvisor for dealing with difficult emotions and life stresses. There was an understanding that life stress is often unavoidable, and participants expressed a desire to learn ways of dealing with stresses. Some participants stated that learning how to manage emotions would act as a replacement for taking antidepressants.

> *I think that exercise of sitting by the stream is very good, because I know when I had Cognitive Behavioural Therapy I was taught to – you know – when your thoughts came – to – and I still do this now – is always remember – say to yourself that it will pass, those feelings will pass and it might be horrible while you're going through those feelings, but find somewhere nice and comfortable to sit, with a blanket even, and that sort of thing.*
>
> *[04/01/0025] [round 3]*

For peer review only - http://bmjopen.bmj.com/site/about/guidelines.xhtml

By the final interviews in the final round, participants' comments were positive with no new issues being identified. This signified the intervention was now ready for further evaluation and feedback in the planned feasibility trial to follow.

Discussion

We developed a digital intervention to support appropriate antidepressant discontinuation. The intervention was developed through a process of triangulation between quantitative and qualitative review evidence, theory, and in-depth qualitative research. 'ADvisor for Patients' is designed to support ways of understanding antidepressants and to help people to withdraw more successfully. It provides resources to build confidence for, and to support, stopping including side-effect management, addressing concerns, depression relapse prevention and stress management. The application of the person-based approach [22–24] has ensured our intervention is grounded a rich understanding of patients' psychosocial context.

Discontinuation can be complex [10], and the digital ADvisor intervention is designed to be an information-based resource to support patients, alongside monitoring and review from their General Practitioner (GP, Family Doctor). A separate digital intervention has been developed for GPs and other primary care professionals, called 'ADvisor: Health Professionals'. The patient intervention will also be used with

For peer review only - http://bmjopen.bmj.com/site/about/guidelines.xhtml

additional brief telephone guidance (up to an hour, spread over three calls by trained psychological practitioners), to support use of the material. Guided digital/internet-based resources have been found to be consistently more effective than unguided digital interventions [37] for mental health problems. Guidance in this context is especially important as patients are withdrawing from pharmacotherapy, thus close monitoring is necessary.

The intervention will be implemented in a feasibility randomised controlled trial, where we will carry out a full qualitative [38] and quantitative [35] process study. We will explore how people engage with the intervention and how it affects their discontinuation experience. On this basis, as in the latter stages of the PBA [24], we will continue to modify the intervention ahead of a fully powered main trial.

There are some limitations to consider. Our recruitment for our qualitative work was from a limited, relatively affluent, geographical area in the south of England. The majority of our participants were women in both the primary qualitative work and the think-aloud interviews. While this does reflect the higher rates of antidepressant use for depression in women [39], it may be that our findings do not accurately reflect the views of men on long-term antidepressants. In the think-aloud interview sample, only nine of the 15 participants were taking antidepressants long-term for depression or low mood. The intervention contains information on preventing depression relapse and focuses on the symptoms of depression and anxiety which may not be

For peer review only - http://bmjopen.bmj.com/site/about/guidelines.xhtml

applicable to these individuals. As such, some members of our sample may not have adequately represented the target population for this intervention, which may have introduced bias in our findings. The average age of participants in our think-aloud interview sample was 55.2 years, which may be a reflection of the typical populations in the geographical locations in this study. In the feasibility trial and main trial phases of intervention testing, further qualitative work will be carried out with a larger and demographically wider population of patients from a range of different areas in the UK.

The researchers conducting the think-aloud interviews were involved in the development of the intervention. This may have resulted in bias when asking questions about the intervention. However in think-aloud interviews the patients often express their views in response to what they see on the page as opposed to solely responding to questions from the researcher. While prompting and follow-up questions might have been affected by researcher bias, patients were not aware the interviewers had designed and written elements of the intervention and were encouraged to provide both positive and negative feedback to the researchers.

To conclude, psychologically informed interventions may improve the chances of effective discontinuation from antidepressants. ADvisor is a theory- evidence-, and person-based digital intervention that may provide this support. The feasibility, clinical and cost-effectiveness of ADvisor now needs to be determined.

For peer review only - http://bmjopen.bmj.com/site/about/guidelines.xhtml

Digital intervention for antidepressant discontinuation

1

For peer review only

For peer review only - http://bmjopen.bmj.com/site/about/guidelines.xhtml

Funding Statement

This work was supported by NIHR Programme Grant for Applied Research (PGfAR)grant number RP-PG-1214-20004.

Acknowledgements

The ADvisor was developed using LifeGuide software and / or methodologies, which was partly funded by the NIHR Southampton Biomedical Research Centre (BRC). The authors would like to acknowledge the work of Emma Maund while working on the REDUCE Programme, who conducted two systematic reviews which informed the intervention development.

Data Sharing

This is a qualitative study and therefore the data is not suitable for sharing beyond what is contained within the report. Further information can be requested from the corresponding author.

Competing Interests

Dr. Kendrick reports grants from National Institute for Health Research, during the conduct of the study. Dr. Moncrieff reports grants from National Institute of Health Research, during the conduct of the study; and is a member of the Council for Evidence-based Psychiatry which is an unfunded organisation, whose mission is to 'communicate evidence of the potentially harmful effects of psychiatric drugs to the people and institutions in the UK that can make a difference'. All other authors have no competing interest to disclose.

For peer review only - http://bmjopen.bmj.com/site/about/guidelines.xhtml

Author contribution

TK led on the grant application for the six-year REDUCE programme. SW conducted primary qualitative interviews which informed the intervention content. AG and HB conducted theoretical modelling, behavioural analysis and developed guiding principles. HB drafted intervention content and discussed with the intervention development team (AG and MG) and the wider team (TK, SW, GL, CM, CD, JM, RL, YN and GA). MG developed the intervention into a digital format using Lifeguide software and led on intervention testing. Think aloud interviews were conducted by HB, SW and TK. RL provided support with recruitment for think aloud interviews. Think aloud transcripts were coded by HB and the results were discussed with AG, GL, TK and CM for interpretation. HB, MG and AG refined the intervention in line with patient feedback, with comments from the wider team when necessary. The manuscript was prepared by HB and AG, and has been approved by all co-authors.

Patient and Public Involvement

Patient and public members of the REDUCE team were involved in discussions about the design and recruitment for this study, and were invited to comment on initial drafts of the interview schedules. Patient and public colleagues viewed prototype intervention content and provided comment on these drafts. Patient and public members of the REDUCE team were included in group discussions about the

For peer review only

feedback from think aloud interviews and any resulting amendments to the intervention content.

For peer review only

For peer review only - http://bmjopen.bmj.com/site/about/guidelines.xhtml

References

1 Health and Social Care Information Centre. Prescriptions Dispensed in the Community - Statistics for England, 2006-2016 - NHS Digital. 2017.https://digital.nhs.uk/data-and-information/publications/statistical/prescriptions-dispensed-in-the-community/prescriptions-dispensed-in-the-community-statistics-for-england-2006-2016-pas (accessed 2 Jul 2018).

2 *Health at a Glance: Europe 2012*. OECD 2012. doi:10.1787/9789264183896-en

3 Mojtabai R, Olfson M. National trends in long-term use of antidepressant medications: results from the U.S. National Health and Nutrition Examination Survey. *J Clin Psychiatry* 2014;**75**:169–77. doi:10.4088/JCP.13m08443

4 Scholes S, Faulding S, Mindell J. Use of prescribed medicines. In: *Health and Social Care Information Centre*. 2014. https://files.digital.nhs.uk/publicationimport/pub16xxx/pub16076/hse2013-ch5-pres-meds.pdf (accessed 25 Jun 2018).

5 Ambresin G, Palmer V, Densley K, *et al.* What factors influence long-term antidepressant use in primary care? Findings from the Australian diamond cohort study. *J Affect Disord* 2015;**176**:125–32. doi:10.1016/j.jad.2015.01.055

6 Cruickshank G, MacGillivray S, Bruce D, *et al.* Cross-sectional survey of patients in receipt of long-term repeat prescriptions for antidepressant drugs in

For peer review only

primary care. *Ment Health Fam Med* 2008;**5**:105–9.

7 Piek E, Kollen BJ, van der Meer K, *et al.* Maintenance Use of Antidepressants in Dutch General Practice: Non-Guideline Concordant. *PLoS One* 2014;**9**:e97463. doi:10.1371/journal.pone.0097463

8 Kendrick T, Stuart B, Newell C, *et al.* Did NICE guidelines and the Quality Outcomes Framework change GP antidepressant prescribing in England? Observational study with time trend analyses 2003–2013. *J Affect Disord* 2015;**186**:171–7. doi:10.1016/j.jad.2015.06.052

9 Ferguson JM. SSRI Antidepressant Medications: Adverse Effects and Tolerability. *Prim Care Companion J Clin Psychiatry* 2001;**3**:22–7. doi:10.4088/PCC.v03n0105

10 Maund E, Dewar-Haggart R, Williams S, *et al.* Barriers and facilitators to discontinuing antidepressant use: A systematic review and thematic synthesis. *J Affect Disord* 2019;**245**:38–62. doi:10.1016/j.jad.2018.10.107

11 Sinclair JE, Aucott LS, Lawton K, *et al.* The monitoring of longer term prescriptions of antidepressants: Observational study in a primary care setting. *Fam Pract* 2014;**31**:419–26. doi:10.1093/fampra/cmu019

12 Eveleigh R, Grutters J, Muskens E, *et al.* Cost-utility analysis of a treatment advice to discontinue inappropriate long-term antidepressant use in primary care. *Fam Pract* 2014;**31**:578–84. doi:10.1093/fampra/cmu043

13 Johnson CF, Macdonald HJ, Atkinson P, *et al.* Reviewing long-term antidepressants can reduce drug burden: A prospective observational cohort

For peer review only

study. *Br J Gen Pract* 2012;**62**:773–9. doi:10.3399/bjgp12X658304

14 Fava GA, Rafanelli C, Grandi S, *et al.* Prevention of Recurrent Depression With Cognitive Behavioral Therapy. *Arch Gen Psychiatry* 1998;**55**:816–20.

15 Fava GA, Gandi S, Zielezny M, *et al.* Cognitive behavioral treatment of residual symptoms in primary major depressive disorder. *Am J Psychiatry* 1994;**151**:1295–9. doi:10.1176/ajp.151.9.1295

16 Huijbers MJ, Spinhoven P, Spijker J, *et al.* Discontinuation of antidepressant medication after mindfulness-based cognitive therapy for recurrent depression: Randomised controlled non-inferiority trial. *Br J Psychiatry* 2016;**208**:366–73. doi:10.1192/bjp.bp.115.168971

17 Kuyken W, Byford S, Watkins E, *et al.* Mindfulness-Based Cognitive Therapy to Prevent Relapse in Recurrent Depression. *J Consult Clin Psychol* 2008;**76**:966–78. doi:10.1037/a0013786

18 Kuyken W, Hayes R, Barrett B, *et al.* Effectiveness and cost-effectiveness of mindfulness-based cognitive therapy compared with maintenance antidepressant treatment in the prevention of depressive relapse or recurrence (PREVENT): a randomised controlled trial. *Lancet* 2015;**386**:63–73. doi:10.1016/S0140-6736(14)62222-4

19 Office for National Statistics. Internet users, UK:2018 Office for National Statistics. 2018;:8.https://www.ons.gov.uk/businessindustryandtrade/itandinternetindustry/bulletins/internetusers/2018

For peer review only

20 Andersson G, Carlbring P, Titoy N, *et al.* Internet interventions for adults with anxiety and mood disorders: A narrative umbrella review of recent meta-analyses. *Can J Psychiatry*

21 Bartholomew LK, Parcel GS, Kok G. Intervention Mapping: A Process for Developing Theory and Evidence-Based Health Education Programs. *Heal Educ Behav* 1998;**25**:545–63. doi:10.1177/109019819802500502

22 Band R, Bradbury K, Morton K, *et al.* Intervention planning for a digital intervention for self-management of hypertension: a theory-, evidence-and person-based approach. doi:10.1186/s13012-017-0553-4

23 Yardley L, Morrison L, Bradbury K, *et al.* The Person-Based Approach to Intervention Development: Application to Digital Health-Related Behavior Change Interventions. *J Med Internet Res* 2015;**17**:e30. doi:10.2196/jmir.4055

24 Yardley L, Ainsworth B, Arden-Close E, *et al.* The person-based approach to enhancing the acceptability and feasibility of interventions. *Pilot Feasibility Stud* 2015;**1**:37. doi:10.1186/s40814-015-0033-z

25 Maund E, Stuart B, Moore M, *et al.* Managing Antidepressant Discontinuation: A Systematic Review. *Ann Fam Med* 2019;**17**:52–60. doi:10.1370/afm.2336

26 Braun V, Clarke V. Using thematic analysis in psychology. *Qual Res Psychol* 2006;**3**:77–101. doi:10.1191/1478088706qp063oa

27 Joffe H, Yardley L. Content and thematic analysis. In: *Research Methods for Clinical and Health Psychology*. London: : Sage 2004. 56–68.

28 Michie S, van Stralen MM, West R. The behaviour change wheel: A new

For peer review only

For peer review only - http://bmjopen.bmj.com/site/about/guidelines.xhtml

method for characterising and designing behaviour change interventions. Published Online First: 2011. doi:10.1186/1748-5908-6-42

29 Michie S, Atkins L, West R. *The behaviour change wheel: a guide to designing interventions*. London: : Silverback Publishing 2015.

30 Bandura A. Social cognitive theory of self-regulation. *Organ Behav Hum Decis Process* 1991;**50**:248–87. doi:10.1016/0749-5978(91)90022-L

31 Murray E, Treweek S, Pope C, *et al.* Normalisation process theory: a framework for developing, evaluating and implementing complex interventions. *BMC Med* 2010;**8**:63. doi:10.1186/1741-7015-8-63

32 Bandura A. Health promotion from the perspective of social cognitive theory. 1998;**13**.http://www.tandfonline.com/doi/pdf/10.1080/08870449808407422 (accessed 2 Oct 2017).

33 Horne R, Chapman SCE, Parham R, *et al.* Understanding Patients' Adherence-Related Beliefs about Medicines Prescribed for Long-Term Conditions: A Meta-Analytic Review of the Necessity-Concerns Framework. *PLoS One* 2013;**8**:e80633. doi:10.1371/journal.pone.0080633

34 Clifford S, Barber N, Horne R. Understanding different beliefs held by adherers, unintentional nonadherers, and intentional nonadherers: Application of the Necessity–Concerns Framework. *J Psychosom Res* 2008;**64**:41–6. doi:10.1016/j.jpsychores.2007.05.004

35 Moore G, Audrey S, Barker M, *et al.* Process evaluation of complex interventions UK Medical Research Council (MRC) guidance.

For peer review only

Digital intervention for antidepressant discontinuation

https://www.mrc.ac.uk/documents/pdf/mrc-phsrn-process-evaluation-guidance-final/ (accessed 3 Aug 2017).

36 Ryan RM, Deci EL. Self-determination theory and the facilitation of intrinsic motivation, social development, and well-being. *Am Psychol* 2000;**55**:68–78.http://www.ncbi.nlm.nih.gov/pubmed/11392867 (accessed 10 May 2019).

37 Baumeister H, Reichler L, Muzinger M, *et al.* The impact of guidance on Internet-based mental health interventions - A systematic review. *Internet Interv* 2014;**1**:205–15. doi:10.1016/j.invent.2014.08.003

38 O'Cathain A, Hoddinott P, Lewin S, *et al.* Maximising the impact of qualitative research in feasibility studies for randomised controlled trials: guidance for researchers. *Pilot Feasibility Stud* 2015;**1**:32. doi:10.1186/s40814-015-0026-y

39 Scholes S, Faulding S, Mindell J. Use of Prescribed Medicines. In: *Health Survey for England 2013: Health, Social Care and Lifestyles.* Leeds: : Health and Social Care Information Centre 2014. www.hscic.gov.uk/pubs/hse2013

For peer review only - http://bmjopen.bmj.com/site/about/guidelines.xhtml

ADvisor Guiding Principles	
Design objectives	*Key (distinctive) design features*
To **build confidence** that discontinuing antidepressant medication is safe and achievable over the long-term	• Offer an evidence-based rationale for how withdrawal and replacement with psychological./behavioural alternatives will help.<br>• Provide withdrawal success stories and examples (modelling).<br>• Address concerns patients may have re withdrawal (side effects, symptoms) from their previous experiences – demonstrate empathy and acknowledge real barriers to change.<br>• Offer motivational support.
To be an accessible, motivating resource that supports patients in managing their withdrawal in a	• Foster autonomy through choice and a non-prescriptive approach, providing explanations for all suggestions.<br>• Offer a broad range of strategies from

Table 1. Guiding Principles for the ADvisor intervention.

Digital intervention for antidepressant discontinuation

manner that aligns with **their preferences**	quick support in managing withdrawal symptoms, to more in-depth modules on a mindful approach to preventing depression relapse, and behavioural strategies for managing day-to-day stressors.<br>• Provide options for self-tailoring to personal experiences and barriers<br>• Provide a simple, attractive interface, with a focus on accessibly of content<br>•

peer review only

Digital intervention for antidepressant discontinuation

1 Table 2. Outline content of the digital intervention.

Content	Description
Reducing and stopping antidepressants	An introduction to the intervention, which addresses motivations behind withdrawal, asking participants to reflect on why they might prefer to discontinue antidepressant treatment. Guidance on when to speak to their GP/nurse and advice on following a tapering regime.
Thinking about antidepressants	Acknowledging that antidepressant treatment is not necessarily required long-term and that the mechanisms are more complex than correcting a serotonin deficiency.
I'm worried about stopping	Addressing participant fears by signposting participants to appropriate resources in ADvisor.
Dealing with withdrawal symptoms	Guidance for dealing with mild withdrawal symptoms (including guided practices for accepting/tolerating unpleasant symptoms). Advice for patients to contact their GP for assistance with moderate or severe withdrawal symptoms.
Keeping well	Relapse prevention techniques grounded in Mindfulness-Based Cognitive Therapy.
Thinking about what you value	Reflection on values and committed action to values (through goal setting), based on Acceptance and Commitment Therapy.
Moving forward	Psychoeducation and techniques for managing distress (e.g. mindfulness and behaviour activation) provided through a distress-management online intervention, Healthy Paths.
My Notes	Where patients can access content from other sections where they have written their own responses (for example their own reasons for wanting to stop antidepressants and their own warning signs and triggers for relapse).
Resources	Direct links to resources in ADvisor (e.g. activity planning and information for family and friends).

2

For peer review only

Digital intervention for antidepressant discontinuation

Characteristics	*N (%)*
Females	9 (60)
Males	6 (40)
Married	11 (73.3)
cohabiting	2 (13.3)
Single	2 (13.3)
Employed	9 (60)
Not currently in employment	6 (40)

For peer review only - http://bmjopen.bmj.com/site/about/guidelines.xhtml

Table 3. Think aloud qualitative study characteristics.

Diagnosis	
Depression/low mood	9 (60)
Fibromyalgia	2 (13.3)
Unknown	2 (13.3)
Urethritis	1 (6.7)
Post Traumatic Stress Disorder	1 (6.7)
Successfully stopped before	8 (53.%)
Currently taking antidepressants	14 (93.3%)
	**Mean (SD)**
Age	55.20 (15.59)
Years on antidepressants	10.43 (7.27)
PHQ-9 score	4.53 (2.50)

For peer review only - http://bmjopen.bmj.com/site/about/guidelines.xhtml

Digital intervention for antidepressant discontinuation

Figure 1. Logic model ADvisor intervention alongside additional components

For peer review only

4

Digital intervention for antidepressant discontinuation

For peer review only

For peer review only - http://bmjopen.bmj.com/site/about/guidelines.xhtml

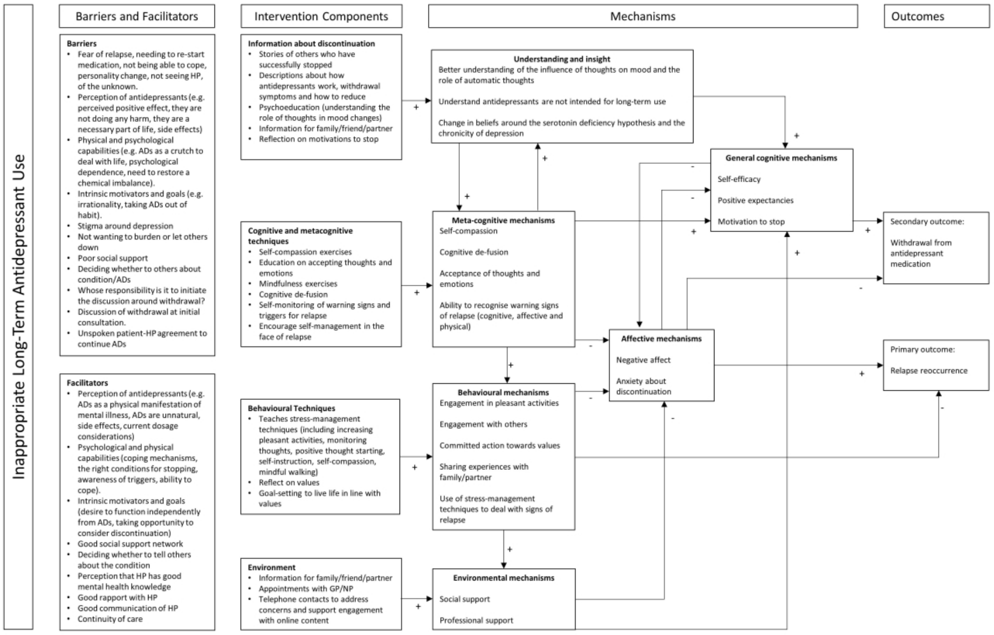

Figure 1. Logic model ADvisor intervention alongside additional components

355x266mm (300 x 300 DPI)

Appendix A – Interview Schedule

REDUCE

UNIVERSITY OF Southampton School of Medicine

REDUCE Study Workstream (WS) 3: REviewing long-term anti-Depressant treatment Use by Careful monitoring in Everyday practice

THINK-ALOUD INTERVIEW SCHEDULE WITH PATIENTS

Below is a list of topics/questions to be discussed in this study. The qualitative work will remain flexible with respect to participants' agendas but we will cover the broad topics/questions noted. It is common in qualitative work to iteratively develop topics and questions as new ideas emerge from early data collection. Therefore, we may add new topics as the interviews progress and data collection continues. However, the key topics of exploring participants' views of the prototype intervention will remain the same.

Introduction

1. Re-introduce self and purpose of interview

2. Check with participant:
- That they are still willing to be interviewed, and to be audio recorded
- Remind them it will take approximately 60 to 90 minutes
- That they are comfortable in a quiet place where they will not be disturbed

3. Remind participant that:
- Their responses will be kept confidential, and quotes used in the results will not identify them as an individual;
- They can change their mind about taking part in the study and stop the interview at any point.

4. Remind the participant that you will start by asking them some questions about their experiences with antidepressants. Remind the participant that you want them to look at the website and use it as they normally would, but say everything that they are thinking out loud. Tell them that you will remind them to do this so that they don't forget as it is very easy to forget and that there are no right or wrong answers as it is their views that are important to us.

5. Ask if the participant has any questions.

6. Start recording.

For peer review only - http://bmjopen.bmj.com/site/about/guidelines.xhtml

Section 1: Demographic Data

We would like to collect some personal information to help us describe the range of people / experiences we have collected, so could you please let me know your

Age	
Gender	M / F
Do you live alone or with someone (friends / partner / family)?	
Single / in a relationship / married?	
Employed / retired / full time carer / stay at home parent?	
Job title	
Currently on ADs?	Y / N
Successfully stopped ADs before?<br>NB. 'Success' = been off ADs & experienced symptom free episode(s).	Y / N
Same GP for review or different GPs within practice?	
Current Medical Diagnosis for ADs (if known)	
Do you pay for your prescriptions?	
Have you ever taken any sick leave from work due to depression / anxiety / stress? If yes, how much?	
Have you ever needed a carer/ or to be cared for due to depression? If yes, by whom?	
Any other medical conditions?	
Have you ever taken St John's Wort?	
Any other relevant information?	
Participant ID	
Date screened by researcher / confirm eligible	
Urban or rural location? (*researcher observation*)	
Deprivation level of area? (*researcher observation*)	

Section 2: Background history of use of antidepressants.

1. Can you tell me a little bit about when you were first prescribed antidepressants?

Prompt: Feelings about how decision to go on antidepressants was made/managed. Experience of taking ADs.

2. Could you describe your experience of taking antidepressants for me now?

Prompt: Any intent to stop? Have you found antidepressants have helped to improve your condition? Side effects/benefits? Expectations of ADs vs. lived experience.

3. Can you tell me about your current depression treatment?

Prompt:
- Regular repeat prescriptions?
- Any self-help or counselling / therapy?
- How often are you reviewed by a GP, nurse or counsellor/therapist? Feelings around frequency?
- Continuity of care?

For peer review only - http://bmjopen.bmj.com/site/about/guidelines.xhtml

- What treatment would you say has helped you most / least?

Section 3: Previous attempts to discontinue / successful withdrawal. Barriers and enablers to discontinuation (including individual / social factors).

1. Can you tell me about a time when you stopped or thought about stopping your antidepressants?

Prompt: What were your reasons for wanting to stop? How long did you stop for? What was it that made you stay on your antidepressants? Withdrawal experiences / effects. How would you feel if you had to restart your antidepressants or increase the dose (if stopped/stopping)? Explore expectations around withdrawal.

Section 4: Think-aloud and researcher prompts

Explain to them that you want them to look at the website and use it as they normally would, but say everything that they are thinking out loud. Tell them that you will remind them to do this so that they don't forget as it is very easy to forget. If you think it would help then get them to try counting the windows in their house whilst saying everything that they are thinking out loud.

- [only on first page] What are your first impressions of this page?
- What are you thinking now?
- What made you choose that option?
- What do you think about [this activity, this information]?
- Can you tell me a bit more about that?
- What is it you like about that?
- That's really interesting......

Section 5: Post-think-aloud questions

- Overall, what do you think about this website?
- Can you tell me about anything that you liked about the website?
- Was there anything that you found surprising in the website?
- Can you tell me anything about the website that you were less keen on?
- Can you tell me about anything that you think should be changed?
- What would you think if your GP or practice nurse asked you to use the website?
- If you were withdrawing from your antidepressants, which parts of AD-visor do you think you would like to look at and why? (E.g. dealing with withdrawal symptoms, information about how antidepressants work, relapse prevention, mindfulness etc.).
- When people use this website for real, they will be offered some support over the telephone. If you were using the programme for real, what would you think of this option to get support over the phone?
- What are your thoughts about telephone support throughout the trial in general? [Researcher to explain trial design].
- If you did have opportunity to have support over the telephone, which of the topics in AD-visor do you think would be most useful to discuss over the phone?

ANY OTHER TOPICS YOU WOULD LIKE TO DISCUSS?

ANY QUESTIONS?

For peer review only - http://bmjopen.bmj.com/site/about/guidelines.xhtml

Debrief

- Tell participant that the digital recorder is now being switched off.
- Thank participant for taking part in the interview.
- Revisit consent
- Ask if the participant has any questions about the study.
- Let them know that you will be sending all participants a summary of study findings.
- Check happy for data to be used for teaching / secondary analysis.
- Thank participant again for taking part in the interview.

For peer review only

For peer review only - http://bmjopen.bmj.com/site/about/guidelines.xhtml

Appendix B – Behavioural Diagnosis

Target behaviour: Reducing and stopping antidepressant medication		
**BCW/COM-B Components**	**What needs to happen for the target behaviour to occur?**	**Proposed intervention element**
**Physical capability**<br>*Physical skill, strength or stamina*	• Understanding how to reduce doses physically: e.g. how to take tapered medication appropriately, in order to reduce the occurrence of side effects.	• **GP**<br>• **Internet intervention modules**<br>• **Telephone support**
**Psychological capability**<br>*Knowledge or psychological skills, strength or stamina to engage in necessary mental processes*	• Detailed, accessible guidance on the withdrawal process in general (setting up appropriate expectations)<br>• Improving knowledge on how to withdraw (practicalities)<br>• Developing psychological skills to manage the process:<br>o Managing psychological side effects of withdrawal<br>o Understanding helpful appraisals of symptoms<br>o Learning about the prevention of relapse, managing fear of recurrence<br>o Developing skills to manage life-stressors cognitively and behaviourally<br>*Social Cognitive Theory (SCT) and research will be broadly drawn on to ensure information/techniques are described and applied to align with evidence-based principles for increasing self-efficacy*	• **Internet intervention modules**<br>• (Telephone support)

For peer review only

**Physical opportunity**<br>*Opportunity afforded by the environment involving time recourses, locations, cues, physical affordance*	• Ability to access and get to GP appointments/pharmacy to collect reduced dose antidepressants	• **General practitioner (as a function of usual care)**<br>• **Telephone support/advice**
**Social opportunity**<br>*Opportunity afforded by interpersonal influences, social cues and cultural norms that influence the way we think about things*	• Close social network (family/friends) of patient may need to be supportive of the withdrawal process/attempt	• **Brief overview material developed for family members/friends**
**Reflective motivation**<br>*Reflective processes involving evaluations/beliefs about what is good and bad, and plans (self-conscious intentions)*	• Modification of beliefs about depression:<br>o Exploring the nature of depression in a way that aligns with behavioural/cognitive management<br>o Discussing impact of beliefs and expectations about chronicity<br>o Exploring effect of analogies with physical conditions (diabetes/asthma)<br>o Acknowledging complexity re our understanding of depression in an accessible manner<br>• Modification of beliefs about antidepressant medication:<br>o Addressing beliefs about addiction/dependency<br>o Exploring the serotonin hypothesis; evidence, balanced implications, rationale for behaviour/cognition to substitute medication	• **Internet intervention modules**<br>• **Internet intervention modules**

For peer review only

	• Foster motivation to withdraw through discussion of benefits, reduction of side effects, potential for increase in agency, potential for effective use of alternatives to pharmacological management<br><br>• Facilitate clear planning for the withdrawal process e.g. human contacts, management strategies, access to rapid/emergency support<br><br>*Inductive qualitative work (meta-synthesis and primary qualitative research) and theory will be used to inform this material*	• **General practitioner**<br><br>• **Telephone support/advice**
**Automatic motivation**<br>*Automatic processes involving emotional reactions, desires (wants and needs) impulses, inhibitions, drive states and reflex responses*	• Encourage awareness of automatic disruptive modes/thought process that may trigger or be triggered by symptoms<br><br>• Work on developing habitual healthier responses to symptom occurrences	• **Internet intervention modules**
**Behavourial diagnosis of the relevant COM-B components**	Although all areas of the COM-B model will need to be addressed to some extent, **psychological capability** and **reflective motivation** are likely to be the key targets for a supported digital intervention to help patients withdraw from antidepressant medication	

References:

1. Michie S, van Stralen MM, West R. The behaviour change wheel: a new method for characterising and designing behaviour change interventions. Implement Sci. 2011;6:42.
2. Michie SF, Atkins L, West R. The behaviour change wheel: a guide to designing interventions. London: Silverback Publishing; 2015

For peer review only

Appendix C – Theoretical Modelling

**Intervention module**	**Page**	**Content**	**Evidence:** Importance of barrier/facilitator content targets OR evidence for effectiveness of content	**BCW construct**	**BCW function**	**BCTs (Taxonomy V1)** Techniques broadly applied across content sections	**SCT construct** Constructs applied across content sections	**NPT construct** Constructs applied across content sections
**Reducing and stopping antidepressants**	Welcome							
	Why should I reduce and stop?	Foster a motivation to withdraw through discussion of benefits, reduction of side effects, potential for increase in agency, potential for effective use of alternatives to medication	*Bosman et al. (2016); Dickinson et al. (2010); Verbeek-Heida and Mathot (2006); Iden et al. (2011); Karp (1993); Knudsen et al. (2002); Eveleigh (2015); Gibson (2016); Schofield (2011).*	Reflexive motivation	Enablement; training; education	9.1 Credible source<br><br>9.2 Pros and cons<br><br>15.2. Persuasion about capability<br><br>13.2 Framing-reframing	Knowledge; social outcome expectations; physical outcome expectations; Self-efficacy (Somatic and emotional states)	Coherence: Individual specification<br><br>Cognitive participation: Initiation
	The downsides	Reflection on the side effects of antidepressants as a means to foster motivation to withdraw		Reflexive motivation	Enablement; training; education			

	When should I reduce and stop?	Highlighting that it is best to start withdrawal at a stable time in life		Psychological capability	Enablement; training; education			
	What to expect	Outline the discontinuation process: that the GP will provide a schedule, that this is flexible and that there may be side effects but there are ways to manage these and they are often short-lived.		Psychological capability	Enablement; training; education			
	Addressing concerns	Briefly acknowledges that many people have concerns about withdrawal but that there are techniques for dealing with this in AD-visor		Psychological capability	Enablement; training; education			
	How can my GP help?	Outline the role of the GP in discontinuation,	*Bosman et al. (2016); Dickenson et al.*	Physical capability	Enablement; training; education			

		when to go to the GP for support.	*2010; Grime & Pollock (2003); Verbeek-Heida and Mathot (2006); Eveleigh (2015); Gibson (2016); Leydon et al. (2007) ; Cartwright (2016)*					
	Planning ahead	Overview of the process: GP will give schedule and as one tapers, there is support in AD-visor that can be used		Reflexive motivation	Enablement; training; education			
	Support from family and friends	Highlight how friends and family members can play and important role	*Bosman et al. (2016); Cromartry (2011); Verbeek-Heida and Mathot (2006); Eveleigh (2015)*	Social opportunity	Enablement; training; education	3.1 Social support 3.3 Social support (emotional)		
**How to reduce antidepressants**	How to reduce	Practical information about tapering schedules		Physical capability	Enablement; training; education	4.1 Instructions on how to perform behaviour	Self-efficacy (Mastery experiences/vic	Coherence: Individual specification

For peer review only - http://bmjopen.bmj.com/site/about/guidelines.xhtml

	How to reduce (2)	Highlight that there is unlikely to be a need for liquid formulations or pill cutters but if needed, the GP can offer some guidance (perhaps via community pharmacist)		Physical capability	Environmental restructuring; Enablement; training; education	6.1 Demonstration of behaviour (modelling)	arious experiences).	
	When to reduce	Reiterate that there are ideal times to begin tapering, such as when no major life events are expected		Psychological capability	Enablement; training; education			
**Thinking about antidepressants**	What are antidepressants?	Briefly explains what antidepressants are used for. Highlights that while it was believed they work through increasing serotonin, we now know it is more complex than that.	*Bosman et al. (2016); Dickenson et al. 2010; Grime & Pollock (2003); Verbeek-Heida and Mathot (2006); Karp (1993); Knudsen et al. (2002); Eveleigh (2015); Gibson (2016); Cartwright*	Reflexive motivation	Enablement; training; education	13.2 Framing/reframing<br><br>15.2. Persuasion about capability	Social outcome expectations; Knowledge; physical outcome expectations	Coherence: Internalisation

For peer review only

	Can I stop taking them?	Key point: even though we don't know exactly *how* they work, we do know that many people can successfully discontinue	*(2016); Leydon et al. (2007).*	Reflexive motivation	Enablement; training; education			
	Other forms of 'antidepressant'	There are things other than medication which can improve mood. The relationship between brain and behaviour is highlighted through a study which shows that CBT can result in changes in the brain		Reflexive motivation	Enablement; training; education			
	How to antidepressants work	Highlights again that we don't know exactly how they work but we do know: ADs help some people and not others and many		Reflexive motivation	Enablement; training; education			

		people can successfully stop.						
**I'm worried about stopping**	I'm worried about stopping	Highlight that many people have concerns about stopping and this is understandable and does not mean you won't be able to discontinue	Bosman et al. (2016); Dickinson et al. (2010); Verbeek-Heida and Mathot (2006); Iden et al. (2011); Karp (1993); Knudsen et al. (2002); Eveleigh (2015); Gibson (2016); Schofield (2011); Leydon et al. (2007).	Psychological capability	Enablement; training; education	13.2 Framing/reframin g<br><br>15.2. Persuasion about capability	Knowledge, Self-efficacy (Mastery experiences vicarious experiences).<br><br>Social outcome expectations; Knowledge; physical outcome expectations	Cognitive participation: Initiation<br><br>Cognitive participation: Activation
	Successful stopping	Indicate that many people stop SD without problems, and those who are worried can overcome their concerns		Psychological capability	Enablement; training; education			
	Concerns about stopping	Patients will be given a selection of options to click on to read more about specific concerns		Psychological capability	Enablement; training; education			
	How will I cope if something big happens?	Reassure that AD-visor has guidance on managing stress in difficult		Psychological capability	Enablement; training; education			

For peer review only - http://bmjopen.bmj.com/site/about/guidelines.xhtml

		situations. Signpost to Moving Forward module.						
	What if I go back to how I was before?	Reassure that AD-visor has guidance on preventing relapse and signpost to Keeping Well module.		Psychological capability	Enablement; training; education			
	What if I have to start taking antidepressants again?	Reassure that hopefully this won't be necessary because they will learn how to prevent relapse, but if it is, they can try withdrawing again in future		Psychological capability	Enablement; training; education			
	How will I manage my responsibilities?	Guidance on planning activities and highlight the importance family support as well as the timing of the tapering process		Psychological capability	Enablement; training; education			

For peer review only

	Dealing with worries	Reflecting on the motivations to discontinue and weighing these up against concerns.		Reflexive motivation	Enablement; training; education			
**Keeping well**	Keeping well	Introduce to the idea of relapse prevention	Kuyken (2008); Allen (2009); Kuyken (2010); Fava (1998); Cromarty (2011); Otto (2010);	Psychological capability	Enablement; training; education	11.2 Reduce negative emotions<br><br>13.2 Framing/reframin g<br><br>6.1 Demonstration of behaviour<br><br>4.3 Reattribution	Knowledge, Goals Self-efficacy (Mastery experiences vicarious experiences).<br><br>Social outcome expectations; Knowledge; physical outcome expectation	Cognitive participation: Activation
	Automatic pilot	Define running on autopilot and explain negative automatic thoughts		Psychological capability	Enablement; training; education			
	The power of thoughts	Explain how the way we think impacts mood and teach cognitive defusion (thoughts are not facts)		Psychological capability	Enablement; training; education			
	Let it be	Defining the term 'acceptance' and why it is useful in dealing		Psychological capability	Enablement; training; education			

		with difficult thoughts and feelings						
	Recognising warning signs	Explaining and reflecting on what thoughts and physical sensations might be indicators of relapse		Psychological capability	Enablement; training; education			
	Recognising triggers	Reflecting on situations that might trigger a relapse		Psychological capability	Enablement; training; education			
	Recognising relapse	Writing down warning signs and triggers and saving these to view later		Psychological capability	Enablement; training; education			
	Responding differently	Highlight that you cannot change thoughts or the things that happen in life, but you have a choice how to respond to these. Responding in more helpful		Psychological capability	Enablement; training; education			

		ways can prevent relapse.						
	Preventing relapse	1. Take a breath<br>2. Make a decision on how to act<br>3. Take action		Psychological capability	Enablement; training; education			
**Living life with values and goals***	What are values	Defines values as like a compass point providing direction for our lives.	Swain et al. 2013; Powers et al. 2009.	Psychological capability	Enablement; training; education	11.2 Reduce negative emotions<br><br>13.2 Framing/reframin g<br><br>6.1 Demonstration of behaviour	Knowledge, Goals	Coherence: Internalisation
	What do I value?	Provides a space to write down what they value		Psychological capability	Enablement; training; education			
	Goals	Explaining the need to set goals in order to		Psychological capability	Enablement; training; education			

		act in line with our values				4.3 Reattribution		
	Setting goals	Guidance and space to write goals		Psychological capability	Enablement; training; education			
	Meeting goals	Reminds users to revisit this section to review their goals and see if they have met them		Psychological capability	Enablement; training; education			
**Dealing with withdrawal symptoms**	What are withdrawal symptoms?	Describes what they are and that they are a consequence of the brain and body adapting to the change in medication	*Bosman et al. (2016); Dickinson et al. (2010); Verbeek-Heida and Mathot (2006); Iden et al. (2011); Karp (1993); Knudsen et al. (2002); Eveleigh (2015); Gibson (2016); Schofield (2011); Leydon et al. (2007)*	Psychological capability Physical capability	Enablement; training; education	13.2 Framing/reframin g<br><br>6.1 Demonstration of behaviour<br><br>4.3 Reattribution	Social outcome expectations; Knowledge; physical outcome expectations	Cognitive participation: Activation
	Recognisin g withdrawal symptoms	This page highlights that there are different symptoms that might be physical or mental. Specific details of what symptoms may occur are not given.		Psychological capability Physical capability	Enablement; training; education			

	Thinking about withdrawal symptoms	Explains that the way we think about symptoms can change how much impact they have (e.g. if you mistake a withdrawal symptom for relapse, it may be harder for the symptom to pass).		Psychological capability Physical capability	Enablement; training; education			
	Knowing the difference	Details about the differences between withdrawal symptoms and relapse.		Psychological capability Physical capability	Enablement; training; education			
	Dealing with withdrawal symptoms	Mild symptoms can be tolerated and will pass, moderate symptoms can be treated by a doctor, and severe symptoms may indicate a slower taper is needed.		Psychological capability Physical capability	Enablement; training; education			

	Accepting withdrawal symptoms	Guidance on accepting/tolerating symptoms based on acceptance and commitment exercises used with chronic physical symptoms		Psychological capability Physical capability	Enablement; training; education			
**Moving forward**	Healthy Paths Through Stress intervention (Healthy Paths). See Geraghty et al. 2017 for full description	This module is based on an intervention aimed at managing life stresses. The modules have been developed as part of a separate project and their content will be incorporated into AD-visor. This section will include guidance on mindfulness practices and behavioural activation.	Muñoz et al. 2005; Geraghty et al. 2016.	Psychological capability	Enablement; training; education	11.2 Reduce negative emotions<br><br>13.2 Framing/reframing<br><br>6.1 Demonstration of behaviour<br><br>4.3 Reattribution	Knowledge, Goals Self-efficacy (Mastery experiences vicarious experiences).<br><br>Social outcome expectations; Knowledge; physical outcome expectations	Coherence: Individual specification<br><br>Coherence: Internalisation<br><br>Cognitive participation: Initiation<br><br>Cognitive participation: Activation

New references added:

Bosman, R. C., Huijbregts, K. M., Verhaak, P. F. M., Ruhé, H. G., Van Marwijk, H. W. J., Van Balkom, A. J. L. M., & Batelaan, N. M. (2016). Long-term antidepressant use: A qualitative study on perspectives of patients and GPs in primary care. *British Journal of General Practice*, *66*(651), e708–e719. https://doi.org/10.3399/bjgp16X686641

Cartwright, C., Gibson, K., & Read, J. (2016). Long-term antidepressant use : patient perspectives of benefits and adverse effects, 1401–1407. https://doi.org/10.2147/PPA.S110632

Cromarty, P., Jonsson, J., Moorhead, S., & Freeston, M. H. (2011). Cognitive Behaviour Therapy for Withdrawal from Antidepressant Medication: A Single Case Series. *Behavioural and Cognitive Psychotherapy*, *39*(01), 77–97. https://doi.org/10.1017/S1352465810000512

Dickinson, R., Knapp, P., House, A. O., Dimri, V., Zermansky, A., Petty, D., … Raynor, D. K. (2010). Long-term prescribing of antidepressants in the older population: A qualitative study. *British Journal of General Practice*, *60*(573), 257–259. https://doi.org/10.3399/bjgp10X483913

Eveleigh, R. H. (2015). *Inappropriate long-term antidepressant use in primary care: A challenge to change*. Radboud University Nijmegen. https://doi.org/10.1063/1.3033202

Fava, G. A., Rafanelli, C., Grandi, S., Conti, S., & Belluardo, P. (1998). Prevention of Recurrent Depression With Cognitive Behavioral Therapy. *Arch Gen Psychiatry*, *55*, 816–820.

Geraghty, A. W. A., Muñoz, R. F., Yardley, L., Mc Sharry, J., Little, P., & Moore, M. (2016). Developing an Unguided Internet-Delivered Intervention for Emotional Distress in Primary Care Patients: Applying Common Factor and Person-Based Approaches. *JMIR Ment Health*, *3*(4), e53.

Gibson, K., Cartwright, C., & Read, J. (2016). “In my life antidepressants have been..”: A qualitative analysis of users’ diverse experiences with antidepressants. *BMC Psychiatry*, *16*(1), 1–7. https://doi.org/10.1186/s12888-016-0844-3

Grime, K. P. J. (2002). Understanding Depression and its Treatment: GP, Patient and Pharmacist Perspectives, (September), 52–60. Retrieved from http://www.pharmacyresearchuk.org/waterway/wp-content/uploads/2012/11/Understanding_depression_and_its_treatment1.pdf

Iden, K. R., Hjørleifsson, S., & Ruths, S. (2011). Treatment decisions on antidepressants in nursing homes: A qualitative study. *Scandinavian Journal of Primary Health Care*, *29*(4), 252–256. https://doi.org/10.3109/02813432.2011.628240

Karp, D. A. (1993). Taking anti-depressant medications: Resistance, trial commitment, conversion, disenchantment. *Qualitative Sociology*, *16*(4), 337–359. https://doi.org/10.1007/BF00989969

Knudsen, P., Hansen, E. H., Traulsen, J. M., & Eskildsen, K. (2002). Changes in Self-Concept While Using SSRI Antidepressants. *Qualitative Health Research*, *12*(7), 932–944. https://doi.org/10.1177/104973202129120368

For peer review only

Kuyken, W., Byford, S., Watkins, E., Holden, E., White, K., King 's, B. B., … Teasdale, J. D. (2008). Mindfulness-Based Cognitive Therapy to Prevent Relapse in Recurrent Depression. *Journal of Consulting and Clinical Psychology*, *76*(6), 966–978. https://doi.org/10.1037/a0013786

Kuyken, W., Watkins, E., Holden, E., White, K., Taylor, R. S., Byford, S., … Dalgleish, T. (2010). How does mindfulness-based cognitive therapy work? *Behaviour Research and Therapy*, *48*, 1105–1112. https://doi.org/10.1016/j.brat.2010.08.003

Leydon, G. M., Rodgers, L., & Kendrick, T. (2007). A qualitative study of patient views on discontinuing long-term selective serotonin reuptake inhibitors. *Family Practice*, *24*(6), 570–575. https://doi.org/10.1093/fampra/cmm069

Muñoz, R. F., & Mendelson, T. (2005). Toward evidence-based interventions for diverse populations: The San Francisco General Hospital prevention and treatment manuals. *J Consult Clin Psychol*, *73*(5), 790-799.

Otto, M. W., McHugh, R. K., Simon, N. M., Farach, F. J., Worthington, J. J., & Pollack, M. H. (2010). Efficacy of CBT for benzodiazepine discontinuation in patients with panic disorder: Further evaluation. *Behaviour Research and Therapy*, *48*(8), 720–727. https://doi.org/10.1016/J.BRAT.2010.04.002

Powers, M. B., Zum Vorde Sive Vording, M. B., & Emmelkamp, P. M. (2009). Acceptance and commitment therapy: a meta-analytic review. *Psychother Psychosom*, *78*(2), 73-80.

Schofield, P., Crosland, A., Waheed, W., Aseem, S., Gask, L., Wallace, A., … Tylee, A. (2011). Patients' views of antidepressants: From first experiences to becoming expert. *British Journal of General Practice*, *61*(585), 142–148. https://doi.org/10.3399/bjgp11X567045

Swain, J., Hancock, K., Hainsworth, C., & Bowman, J. (2013). Acceptance and commitment therapy in the treatment of anxiety: a systematic review. *Clin Psychol Rev*, *33*(8), 965-978.

Verbeek-Heida, P. M., & Mathot, E. F. (2006). Better safe than sorry — why patients prefer to stop using selective serotonin reuptake inhibitor (SSRI) antidepressants but are afraid to do so: results of a qualitative study. *Chronic Illness*, *2*(2), 133–142. https://doi.org/10.1179/174592006X111003

For peer review only

For peer review only

Standards for Reporting Qualitative Research (SRQR)*

http://www.equator-network.org/reporting-guidelines/srqr/

Page/line no(s).

Title and abstract

**Title** - Concise description of the nature and topic of the study Identifying the study as qualitative or indicating the approach (e.g., ethnography, grounded theory) or data collection methods (e.g., interview, focus group) is recommended	Page 1
**Abstract** - Summary of key elements of the study using the abstract format of the intended publication; typically includes background, purpose, methods, results, and conclusions	Page 2

Introduction

**Problem formulation** - Description and significance of the problem/phenomenon studied; review of relevant theory and empirical work; problem statement	Page 4 line 4-30
**Purpose or research question** - Purpose of the study and specific objectives or questions	Page 5 line 1-10

Methods

**Qualitative approach and research paradigm** - Qualitative approach (e.g., ethnography, grounded theory, case study, phenomenology, narrative research) and guiding theory if appropriate; identifying the research paradigm (e.g., postpositivist, constructivist/ interpretivist) is also recommended; rationale**	Page 7, line 17-19
**Researcher characteristics and reflexivity** - Researchers' characteristics that may influence the research, including personal attributes, qualifications/experience, relationship with participants, assumptions, and/or presuppositions; potential or actual interaction between researchers' characteristics and the research questions, approach, methods, results, and/or transferability	Page 22 line 1-7
**Context** - Setting/site and salient contextual factors; rationale**	Page 8 line 1-17
**Sampling strategy** - How and why research participants, documents, or events were selected; criteria for deciding when no further sampling was necessary (e.g., sampling saturation); rationale**	Page 7 line 22-31<br>Page 8 line 14-17
**Ethical issues pertaining to human subjects** - Documentation of approval by an appropriate ethics review board and participant consent, or explanation for lack thereof; other confidentiality and data security issues	Page 7 line 19-20
**Data collection methods** - Types of data collected; details of data collection procedures including (as appropriate) start and stop dates of data collection and analysis, iterative process, triangulation of sources/methods, and modification of procedures in response to evolving study findings; rationale**	Page 8 line 1-38

For peer review only - http://bmjopen.bmj.com/site/about/guidelines.xhtml

**Data collection instruments and technologies** - Description of instruments (e.g., interview guides, questionnaires) and devices (e.g., audio recorders) used for data collection; if/how the instrument(s) changed over the course of the study	Page 8 line 1-17
**Units of study** - Number and relevant characteristics of participants, documents, or events included in the study; level of participation (could be reported in results)	Page 30 Table 3
**Data processing** - Methods for processing data prior to and during analysis, including transcription, data entry, data management and security, verification of data integrity, data coding, and anonymization/de-identification of excerpts	Page 8 line 1-17
**Data analysis** - Process by which inferences, themes, etc., were identified and developed, including the researchers involved in data analysis; usually references a specific paradigm or approach; rationale**	Page 8 line 20-33
**Techniques to enhance trustworthiness** - Techniques to enhance trustworthiness and credibility of data analysis (e.g., member checking, audit trail, triangulation); rationale**	Page 8 line 27-29

Results/findings

**Synthesis and interpretation** - Main findings (e.g., interpretations, inferences, and themes); might include development of a theory or model, or integration with prior research or theory	Page 13 line 27 to page19 line 22
**Links to empirical data** - Evidence (e.g., quotes, field notes, text excerpts, photographs) to substantiate analytic findings	Page 13 line 27 to page19 line 22

Discussion

**Integration with prior work, implications, transferability, and contribution(s) to the field -** Short summary of main findings; explanation of how findings and conclusions connect to, support, elaborate on, or challenge conclusions of earlier scholarship; discussion of scope of application/generalizability; identification of unique contribution(s) to scholarship in a discipline or field	Page 19 line 26 to Page 20 line 10
**Limitations** - Trustworthiness and limitations of findings	Page 20 line 18 to page 21 line 7

Other

**Conflicts of interest** - Potential sources of influence or perceived influence on study conduct and conclusions; how these were managed	Page 22 line 11-16
**Funding** - Sources of funding and other support; role of funders in data collection, interpretation, and reporting	Page 22 line 2-3

*The authors created the SRQR by searching the literature to identify guidelines, reporting standards, and critical appraisal criteria for qualitative research; reviewing the reference lists of retrieved sources; and contacting experts to gain feedback. The SRQR aims to improve the transparency of all aspects of qualitative research by providing clear standards for reporting qualitative research.

For peer review only - http://bmjopen.bmj.com/site/about/guidelines.xhtml

**The rationale should briefly discuss the justification for choosing that theory, approach, method, or technique rather than other options available, the assumptions and limitations implicit in those choices, and how those choices influence study conclusions and transferability. As appropriate, the rationale for several items might be discussed together.

Reference:
O'Brien BC, Harris IB, Beckman TJ, Reed DA, Cook DA. **Standards for reporting qualitative research: a synthesis of recommendations.** *Academic Medicine*, Vol. 89, No. 9 / Sept 2014 DOI: 10.1097/ACM.0000000000000388

For peer review only

For peer review only - http://bmjopen.bmj.com/site/about/guidelines.xhtml
